# Comparative Accuracy of Machine Learning and GBLUP for Predicting Genomic Estimated Breeding Values in Chickens

**DOI:** 10.3390/genes17030315

**Published:** 2026-03-12

**Authors:** Haoxiang Chai, Yuqi Yang, Dan Wang, Chao Ning, Xuguang Zhang, Wenwen Wang, Qin Zhang, Haigang Bao, Hui Tang

**Affiliations:** 1 Shandong Provincial Key Laboratory for Livestock Germplasm Innovation & Utilization, College of Animal Science and Technology, Shandong Agricultural University, Tai’an 271018, China; chxlh980@163.com (H.C.); wangd18@sdau.edu.cn (D.W.); ningchao@sdau.edu.cn (C.N.); zhangxuguang001@icloud.com (X.Z.); wangwenwen@sdau.edu.cn (W.W.); qzhang@sdau.edu.cn (Q.Z.); 2 National Engineering Laboratory for Animal Breeding, College of Animal Science and Technology, China Agricultural University, Beijing 100193, China; yqiyang2026@163.com

**Keywords:** machine learning, genomic selection, egg-laying chicken, GEBV, prediction, SNP density

## Abstract

Background: Machine learning (ML) holds great promise for genomic breeding value prediction in livestock and poultry, yet its application in layer breeding remains limited. Methods: In this study, we used whole-genome resequencing data from 834 Wenshui Luhua Green-Shelled (WLGS) laying hens to predict genomic breeding values for eight egg production and egg quality traits using multilayer perceptron (MLP), random forest (RF), and genomic best linear unbiased prediction (GBLUP). Model performance was evaluated via 10-fold cross-validation, and the effects of data type and single nucleotide polymorphism (SNP) density were examined. Results: Heritability analysis indicated moderate heritability for egg number (EN) at 0.327. Egg weight-related traits (EW-30W, EW-40W, and EHD-40W) exhibited high heritability (0.570–0.631), while eggshell strength (ESS-40W) and thickness (EST-40W) showed moderate heritability at 0.228 and 0.220, respectively. Model comparisons revealed that RF performed best for egg shape index (ESI-30W, 0.395) and most egg quality traits, whereas GBLUP yielded optimal results for egg weight traits, achieving prediction accuracies of 0.392 for EW-30W and 0.432 for EW-40W. Whole-genome resequencing data consistently outperformed 50K chip data across all models, with GBLUP improving EW-40W prediction accuracy by 24.9%. SNP density analysis further showed that GBLUP remained stable under low-density conditions, while MLP and RF progressively improved with increasing density, with RF demonstrating the most pronounced advantage at high densities. Conclusions: In summary, the GBLUP model is suitable for traits with high heritability and low-density marker scenarios, while the RF model demonstrates significant predictive advantages for egg production and specific egg quality traits under high-density conditions. This study provides scientific basis for model selection in the genomic selection program for laying hens.

## 1. Introduction

The Wenshui Luhua Green-Shelled (WLGS) laying hen is a distinct breed developed by crossing Wenshang Speckled Chickens with Green-Shelled Egg Chickens, characterized by its speckled plumage, green eggshells, and high egg-laying performance [1].

Traditional genomic selection (GS) methods, exemplified by genomic best linear unbiased prediction (GBLUP), are centered on the assumption of additive genetic effects and estimate individual breeding values by constructing a genomic relationship matrix [2,3]. This method assumes a linear relationship between genotype and phenotype, with marker effects following a normal distribution. It performs well in dissecting complex traits controlled by many small-effect additive loci and has been widely applied in the breeding of major livestock and poultry species, including dairy cattle, pigs, and chickens, with notable success [4,5,6]. The GBLUP method typically assumes a linear relationship between genotypes and phenotypes, incorporating only additive genetic effects in the analysis. However, in complex traits shaped by polygenic interactions and environmental influences, nonlinear genotype–phenotype relationships are common, substantially constraining the predictive capacity of GBLUP. This limitation becomes particularly pronounced when using whole-genome resequencing data, where the number of single nucleotide polymorphism (SNP) markers greatly exceeds the sample size. Under such high-dimensional conditions, the linear GBLUP model is prone to over-parameterization, increasing the risk of overfitting and thereby compromising model generalizability and predictive stability [7,8,9]. This limitation underscores the necessity of exploring new methods capable of capturing non-additive effects and nonlinear relationships in high-dimensional data.

In recent years, machine learning (ML) methods have been progressively introduced into livestock and poultry genomic prediction due to their flexibility in handling high-dimensional data and modeling nonlinear relationships [10]. Random Forest (RF), a representative ensemble learning method, offers several key advantages. By constructing multiple decision trees based on bootstrap sampling and random feature subsampling, RF effectively alleviates multicollinearity issues prevalent in high-dimensional single nucleotide polymorphism (SNP) data. The method is robust to noise and outliers, enabling stable performance without extensive feature engineering. Moreover, RF can automatically capture higher-order interactions and nonlinear relationships among SNPs, a capability essential for dissecting complex traits influenced by epistatic effects [11,12]. For instance, Mota et al. [13] reported that in beef cattle, RF achieved predictive performance comparable to, or even better than, traditional GBLUP for feed efficiency traits. Similarly, Safari et al. [14] demonstrated that RF effectively identifies key SNP markers associated with wool quality traits in sheep.

The Multilayer Perceptron (MLP), a classic feedforward neural network, offers several key advantages. It possesses strong nonlinear function approximation capabilities and can theoretically model arbitrarily complex nonlinear relationships. Moreover, through its multi-layer architecture, MLP enables hierarchical abstraction of input features, allowing it to automatically learn layered representations from SNPs to phenotypes. In addition, MLP can integrate dominance effects, epistatic effects, and their combined patterns, thereby providing a more comprehensive characterization of genetic effects [15,16]. In livestock breeding applications, Chen et al. [17] reported that MLP outperformed traditional linear models in predicting abdominal fat traits in broilers. Furthermore, Zhang et al. [18] achieved high-precision classification of purebred and crossbred pigs using deep learning, demonstrating the representational capacity of neural networks for analyzing high-dimensional SNP data.

In animal breeding, heritability, defined as the proportion of phenotypic variance attributable to additive genetic variance, is a key parameter that determines the rate of selection response and guides the design of breeding strategies. Traits with high heritability can be effectively improved through individual selection, whereas those with low heritability require more accurate prediction methods, such as pedigree-based or genomic selection, to achieve genetic gain. It is important to note that in the standard GBLUP model, residual variance includes both non-additive genetic effects, such as dominance and epistasis, and environmental variance. Due to model assumptions, however, these components cannot be disentangled. Consequently, accurately estimating heritability and recognizing the potential presence of non-additive effects within residual variation are essential for selecting appropriate breeding strategies and prediction models. In this context, the present study first estimates genetic parameters for target traits in WLGS laying hen and orders traits by heritability to provide a genetic basis for subsequent method comparisons.

However, research on the application of machine learning methods to genomic breeding in local chicken breeds remains relatively limited. In particular, for distinctive genetic resources such as the WLGS laying hen, systematic comparative studies evaluating the predictive performance of RF, MLP, and traditional GBLUP are still lacking. Most existing studies have focused on dairy cattle, beef cattle, and commercial laying hens, leaving machine learning-based genomic selection for the unique genetic architecture and complex traits of China’s local chicken breeds largely unexplored. Hence, this study aimed to evaluate the applicability of ML for genomic prediction in this breed. By comparing the predictive accuracy of RF, MLP, and GBLUP models across different SNP datasets, we seek to provide a methodological foundation for the genetic improvement of specialty chicken breeds.

## 2. Materials and Methods

### 2.1. Populations and Phenotypes

Hens were provided by Shandong Jinqiu Animal Husbandry Technology Co., Ltd. (Wenshang County, Jining City, Shandong Province, China). Experiments were performed at the company’s poultry breeding base in Wenshang County, Jining City, Shandong Province, China. All experimental procedures, animal housing, and management practices were approved by the Animal Care and Use Committee of the College of Animal Science and Technology, Shandong Agricultural University (Approval No. SDAUA-2024-030). Laboratory analyses for egg quality were performed at the Poultry Science Laboratory of the same institution.

A total of 834 WGSL laying Hens from the same hatch cohort were selected for the experiment. The birds were reared indoors in cages under management conditions consistent with the Lu Hua Chicken breeding standards. Prior to the trial, the poultry house was thoroughly disinfected and cleaned, with the ambient temperature maintained between 18 °C and 30 °C. A photoperiod of 16 h of light per day was maintained at an intensity of 20 lux; feed and water were provided ad libitum throughout the experiment. Vaccination programs and disease prevention initiatives are carried out under veterinary guidance. As a distinct local Chinese breed, WGSL laying hens currently lack standardized rearing protocols; therefore, this study adopted the NY/T 33-2004 standard as a reference [19]. A single-factor experimental design was implemented. Egg production data were recorded for the first three months following the onset of lay.

This study measured eight traits, including egg production (EN) and egg quality indicators such as egg weight at 30 weeks (EW-30W), egg shape index at 30 weeks (ESI-30W), egg weight at 40 weeks (EW-40W), egg horizontal diameter at 40 weeks (EHD-40W), eggshell strength at 40 weeks (EST-40W), egg shape index at 40 weeks (ESI-40W), and eggshell thickness at 40 weeks (EST-40W).Egg weight was measured using an electronic balance (Model FA1104B, Suzhou Yuheng Electronic Scale Co., Ltd., Suzhou, China) with an accuracy of 0.1 g. Egg length and width were measured with a vernier caliper, and the shape index was calculated. Shell thickness was determined using an eggshell thickness gauge (Model GSA-1020, ORKA Co., Ltd., Tokyo, Japan), and shell strength was tested with an eggshell strength tester (Model EGG-0503, Robotmation Co., Ltd., Shibuya, Japan).

### 2.2. Genotype Data

In this study, blood samples were collected from the wing veins of 834 female Wenshui Green-Shelled Laying Hens and preserved in heparin anticoagulant tubes. Whole-genome resequencing was conducted on the BGI MGI2000 platform using DNBSEQ technology [20]. After quality control, the average alignment rate reached 99.78%, with an individual average sequencing depth of 11.9×. Coverage analysis revealed that regions with coverage of at least 1×, 5×, 10×, and 15× accounted for 96.10%, 86.38%, 60.49%, and 31.43% of the genome, respectively. The raw sequencing reads were processed and subjected to quality control using PLINK 1.9. Variants were filtered according to the following criteria: missing genotype rate < 10%, minor allele frequency (MAF) > 0.05, and Hardy–Weinberg equilibrium (HWE) *p*-value > 1 × 10^−6^. Genotype imputation was subsequently conducted using Beaglev5.1.Quality parameters for sequencing data are provided in Supplementary Table S6.

To evaluate the influence of SNP density on genomic selection accuracy, we used PLINK 1.9 software to extract the loci corresponding to the Jingxin-1 50K chip from whole-genome resequencing data, thereby generating a raw chip dataset. Quality control was then applied by sequentially filtering out SNPs with a missing genotype rate exceeding 10%, a MAF below 0.05, or a HWE test *p*-value less than 0.000001. This process yielded a high-quality chip dataset suitable for comparative analyses with the whole-genome resequencing data [21,22].

### 2.3. Statistical Model

Based on whole-genome resequencing and 50K chip datasets, this study used multilayer perceptron, random forest, and GBLUP to predict genomic breeding values.

#### 2.3.1. GBLUP

For the GBLUP model, variance components and individual genomic estimated breeding values (GEBVs) were estimated using the GMAT v1.01 software [23] based on a single-trait GBLUP model. Additive genetic variance and residual variance were estimated via restricted maximum likelihood (REML) with an iterative convergence criterion set to 10^−6^. Individual GEBVs were then predicted using the estimated variance components. The GBLUP model is expressed as follows:

y=Xb+Zu+e

In the above formula, y is the vector of phenotypic observations, b is the vector of fixed effects, u is the vector of individual additive genetic effects, e is the vector of random residuals; X and Z are the design matrices corresponding to the fixed effects and the additive genetic effects, respectively.

$$u∼N(0,G\sigma_{g}^{2}),\;\;\;e∼N(0,I\sigma_{e}^{2})$$

where G refers to the genomic relationship matrix and I is the identity matrix, with $\sigma_{g}^{2}$ and $\sigma_{e}^{2}$ indicating the additive genetic variance and residual variance, respectively.

Heritability is calculated as the ratio of the additive genetic variance $\left(\sigma_{g}^{2}\right)$ to the phenotypic variance $\left(\sigma_{p}^{2}\right)$.

The formula for calculating heritability is as follows:

$$h^{2}=\frac{\sigma_{g}^{2}}{\sigma_{p}^{2}}$$

This represents the phenotypic variance, typically estimated through variance analysis using the following formula:

$$\sigma_{p}^{2}=\sigma_{g}^{2}+\sigma_{e}^{2}$$

Additive genetic variance and residual variance were estimated using the restricted maximum likelihood (REML) method in the GMAT v1.01 software [23] based on the aforementioned single-trait GBLUP model. Following estimation, heritability for each trait was calculated according to the formula.

#### 2.3.2. Multilayer Perceptron Model

The Multilayer perceptron (MLP) model captures complex nonlinear relationships between markers and traits through layered transformations, with network weights optimized via backpropagation. Implementation was based on the MLPRegressor from scikit-learn (v1.8.0) [24,25]. The preprocessing involved standardizing SNP markers to zero mean and unit variance, followed by dimensionality reduction via univariate filter-based feature selection (SelectKBest with f_regression) to retain the top-k SNPs [26]. Subsequently, hyperparameter tuning was performed using grid search combined with 10-fold cross-validation on the training set, with a focus on hidden_layer_sizes, learning_rate_init, learning_rate, and alpha. Early stopping was applied to prevent overfitting.

#### 2.3.3. Random Forest Model

The Random Forest (RF) model is an ensemble algorithm that integrates multiple decision trees for regression. Each tree is trained on a bootstrap sample of the data, and a random subset of features is considered at each split, promoting model diversity and generalization [27]. The model was implemented using the RandomForestRegressor from scikit-learn (v1.8.0). Feature selection followed the same univariate filter strategy as used for the MLP [28]. Key hyperparameters (n_estimators, max_depth, max_features) were optimized via grid search combined with 10-fold cross-validation on the training set. The final model was retrained on the full training set using the optimal parameters and evaluated on an independent test set.

### 2.4. Evaluation of Genome Prediction Accuracy

The accuracy of genomic breeding value prediction was quantified as the Pearson correlation coefficient (r) between the GEBVs and the corresponding observed phenotypic values. A repeated 10-fold CV scheme was employed: in each of 10 independent iterations, 10% of the samples were randomly retained as a validation set, with the remaining 90% serving as the reference (training) set. The final reported prediction accuracy is the mean Pearson correlation coefficient averaged across all 100 validation folds. In addition to accuracy, this study also assessed prediction bias, defined as the regression coefficient (b_1_) obtained by regressing observed phenotypes on predicted GEBVs across all validation samples. A b_1_ value of 1 indicates perfect unbiasedness; b_1_ < 1 reflects prediction inflation (overestimation of extreme values), and b_1_ > 1 reflects prediction shrinkage (regression toward the mean)

## 3. Results

### 3.1. Phenotypic Summary Statistics of WLGS Laying Hens

The mean and median values for most traits in Table 1 are very close, exhibiting a symmetrical normal distribution, making them suitable for genomic prediction using linear models.

### 3.2. Heritability of Eight Egg Production Traits and Egg Quality Traits

Heritability analysis revealed moderate heritability for egg production (ELR, 0.33), while egg quality traits generally showed high heritability. Among these, traits related to egg size and dimensions, specifically EW-30W, EW-40W, and EHD-40W, displayed the highest estimates (0.57–0.66). Conversely, eggshell strength traits (ESS-40W and EST-40W) exhibited moderate heritability, ranging from 0.22 to 0.37. The complete heritability estimates are presented in Table 2.

### 3.3. The Impact of Different Models on Prediction Accuracy

**Figure 1:**
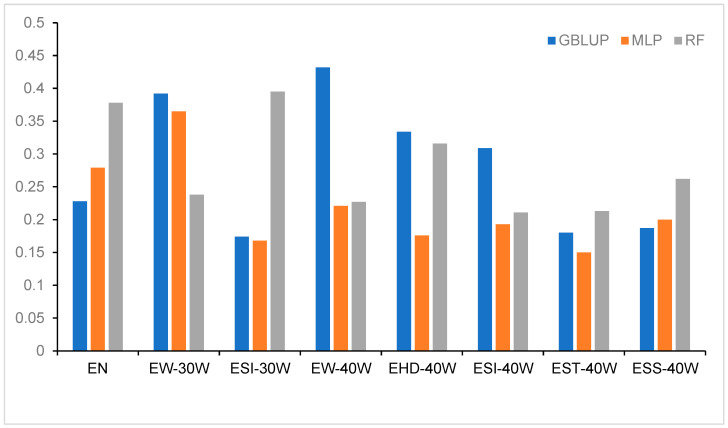
Prediction accuracy for eight traits using three methods based on whole-genome resequencing data and 10-fold cross-validation.

Figure 1 presents the cross-validation results of genomic estimated breeding value predictions using three distinct models, namely, RF, GBLUP, and MLP, based on low-depth sequencing data. For egg production and several egg quality traits, RF achieved the highest prediction accuracy, followed by GBLUP, while MLP exhibited the least favorable performance. Specifically, across the five egg quality traits (ESI-30W, EHD-40W, ESI-40W, EST-40W, and ESS-40W), the mean prediction accuracies for RF, GBLUP, and MLP were 0.279, 0.237, and 0.177, respectively. RF demonstrated superior predictive accuracy for traits including EN, ESI-30W, EST-40W, and ESS-40W. In the case of ESI-30W, RF attained an accuracy of 0.395, substantially outperforming GBLUP (0.174) and MLP (0.168). In contrast, for egg weight traits (EW-30W and EW-40W), GBLUP delivered the highest prediction accuracy, reaching 0.392 and 0.432, respectively.

This study further evaluates the prediction bias of each model, defined as the slope (b_1_) of the linear regression between observed phenotypic values and model predictions, as presented in Table 3. GBLUP exhibited bias values closest to 1 across all traits, ranging from 0.96 to 1.05, indicating good calibration. In contrast, RF and MLP showed some bias for specific traits: RF displayed prediction shrinkage for ESI-30W (b_1_ = 1.20) and ESS-40W (b_1_ = 1.17), while MLP showed prediction inflation for EW-30W (b_1_ = 0.92) and EW-40W (b_1_ = 0.89). These results suggest that although machine learning models enhance prediction accuracy, they may do so at the expense of unbiasedness. Therefore, bias correction methods should be considered in practical breeding applications.

### 3.4. Impact of Whole-Genome Resequencing Data and Chip Data on Prediction Accuracy

**Figure 2:**
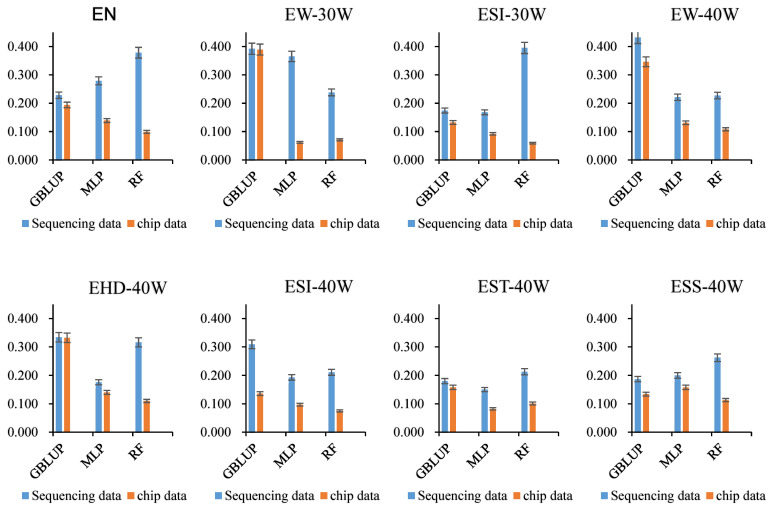
GEBV prediction using whole-genome resequencing data and 50K chip data, respectively, based on 10-fold CV.

Following quality control, 10,221,409 SNPs were retained from an initial 17,299,546. A comparison of prediction accuracy between whole-genome resequencing data and a simulated 50K chip dataset was conducted using GBLUP, MLP, and RF models (Figure 2). The resequencing data provided consistently higher accuracy whether the model applied was MLP, RF, or the traditional GBLUP. A prominent example was observed for trait EW-40W, where GBLUP achieved an accuracy of 0.432 with resequencing data, surpassing the chip-based accuracy of 0.346 by 24.9%.

### 3.5. Effect of Different Site Numbers on Model Prediction Accuracy

**Figure 3:**
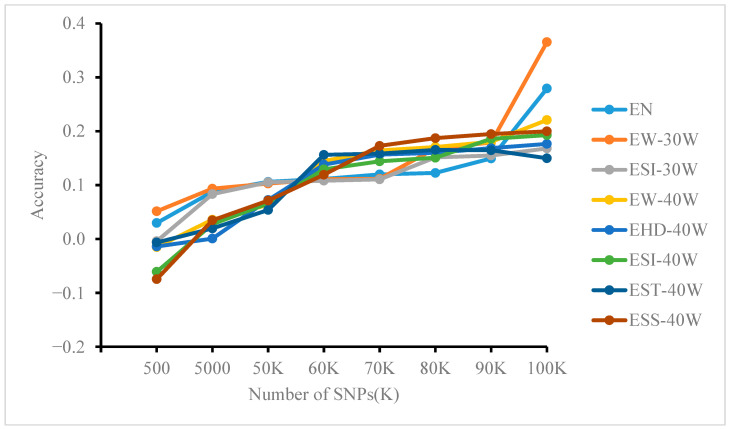
Effect of controlling SNP numbers via feature selection on the accuracy of MLP model predictions for genomic estimated breeding values.

**Figure 4:**
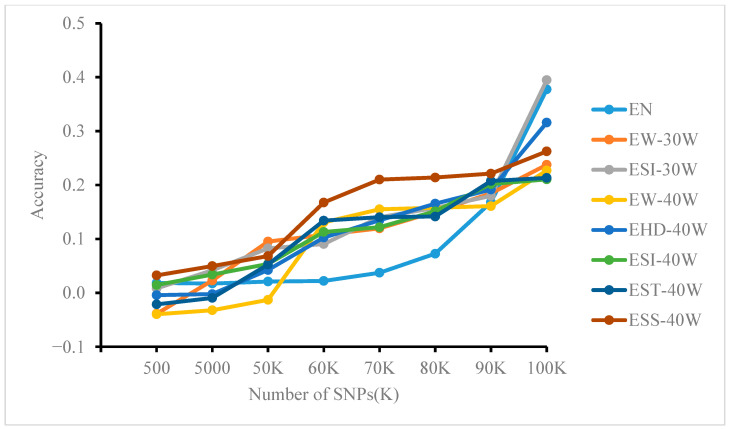
Effect of controlling SNP numbers via feature selection on the accuracy of RF model predictions for genomic estimated breeding values.

**Figure 5:**
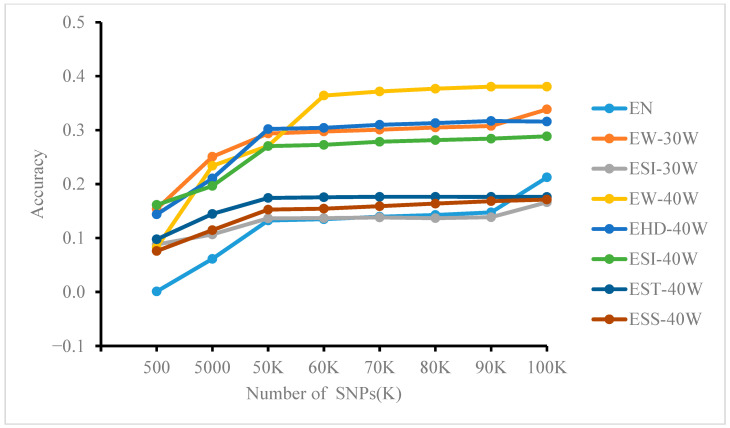
Effect of controlling SNP numbers via feature selection on the accuracy of predicting genomic breeding values using the GBLUP model.

Based on whole-genome resequencing data subsampled to different SNP densities (as described in Section 2.3.2). The prediction accuracy of the GBLUP, MLP, and RF models at varying numbers of loci is shown in Figure 3, Figure 4 and Figure 5. Significant differences were observed among methods and across traits. In general, GBLUP tended to exhibit higher accuracy at lower SNP densities. As density increased, however, the performance of MLP and RF improved progressively, with RF showing a particularly strong advantage. Notably, in the MLP model, accuracy growth for most traits (except EW-30W and EN) plateaued after approximately 60K SNPs. In contrast, RF accuracy increased markedly when SNP counts reached 90K. For GBLUP, little to no improvement was observed beyond about 70K SNPs.

Further analysis across SNP densities revealed distinct performance trends. At lower SNP densities (e.g., k = 500), GBLUP achieved the highest accuracy for most traits, while MLP and RF both showed accuracies below 0.1. As SNP density increased, the accuracy of all models improved. GBLUP reached 0.251 for EW-30W at k = 5000 and 0.302 for EHD-40W at k = 50K. Meanwhile, MLP and RF performance improved progressively. From k = 60K, RF outperformed GBLUP on ESS-40W (0.168). At densities of 70K–90K, RF and MLP surpassed GBLUP for multiple traits, including ESI-30W, ESS-40W, and ELR. By k = 100K, both MLP and RF outperformed GBLUP on most traits, with RF showing a clearer advantage. This pattern indicates that the predictive superiority of ML methods is strongly dependent on higher marker density, as their capacity to capture complex genetic architectures increases with more SNPs, eventually enabling them to outperform traditional methods.

## 4. Discussion

The results indicate that model predictive performance was closely associated with trait heritability. For high-heritability traits, specifically EW-30W, EW-40W, and EHD-40W, with heritability ranging from 0.57 to 0.63, GBLUP achieved higher predictive accuracy. In contrast, for traits with moderate to low heritability, MLP and RF substantially outperformed GBLUP. Specifically, compared with GBLUP, MLP improved prediction accuracy by 1.06% for ESI-30W, 16.6% for ESS-40W, 22.4% for EN, and 7.9% for EW-30W. RF showed even larger gains, with increases of 136.0% for ESI-30W, 21% for EST-40W, 53.1% for ESS-40W, and 65.8% for EN relative to GBLUP. These findings are consistent with those reported in Holstein bulls, supporting the view that model performance is highly dependent on the genetic architecture of the traits [29].

The advantages of machine learning methods over parametric approaches often depend on sample size, marker density, and the genetic architecture of the trait [30]. Thus, in practice, model selection should be guided by trait heritability: GBLUP remains advantageous for high-heritability traits, whereas machine learning models offer greater potential for traits with moderate to low heritability.

Although methods such as Support Vector Machine (SVM), Gradient Boosting Tree (GBDT), and Extreme Gradient Boosting (XGBoost) have been explored for genomic prediction, they often face practical challenges. These models are typically highly sensitive to hyperparameter tuning, computationally intensive, and prone to overfitting or instability when applied to high-dimensional SNP data with limited sample sizes [31]. Moreover, deeper neural network architectures generally require larger sample sizes and greater computational resources, and their advantages in medium-sized livestock and poultry populations remain inconclusive. In this context, RF and MLP offer a more favorable balance among model complexity, computational cost, and predictive performance, making them suitable representatives for systematically comparing ML with traditional GS models.

Our study confirmed that whole-genome resequencing data generally enhanced the accuracy of genomic prediction for economic traits in WLGS laying hens compared to traditional 50K chip data. In the MLP and RF models, resequencing increased the average prediction accuracy by approximately 0.13–0.17. The GBLUP model also showed improvements ranging from 14% to 40% for most traits, consistent with reports that even low-coverage sequence data can match or exceed the performance of high-density SNP chips [32,33]. Notably, however, the advantage of resequencing was minimal for traits EW-30W and EHD-40W, where GBLUP accuracy improved by only 0.83% and 0.67%, respectively. This limited gain aligns with observations in other poultry studies, where dramatic increases in SNP number sometimes yield only marginal accuracy improvements [34]

This inconsistency likely arose from multiple factors. Among these factors, the limited training sample size in this study (834 individuals) is likely a key constraint. A small training population restricts the full utilization of information from sequencing data, as it is difficult to comprehensively capture the effects of low-frequency alleles in small populations. Previous studies have shown that training population size is a critical determinant of genomic prediction accuracy. As sample size increases, models become more robust to noise and yield more precise estimates of rare variant effects [35]. Furthermore, larger samples reshape the goals of feature selection. In small-sample settings, feature selection primarily serves to reduce dimensionality and prevent overfitting, whereas large samples allow researchers to incorporate more markers, thereby enabling a more comprehensive dissection of the genetic architecture underlying complex traits [36]. It is important to emphasize that multi-omics data alone cannot substitute for the fundamental improvement in predictive capability conferred by sufficient sample size [37]. Second, stringent quality control may have removed some low-frequency or functionally relevant SNPs, thereby attenuating the predictive value of sequence data [38]. Furthermore, different algorithms vary inherently in modeling dense linkage-disequilibrium patterns, which can also lead to variable performance across traits [39]. SNP density and sample size interact such that when the sample size is limited, the marginal improvement in predictive accuracy from increasing marker density diminishes [40].Therefore, while whole-genome resequencing generally offers superior accuracy, its effectiveness depends on trait architecture, sample size, QC stringency, and the choice of prediction algorithm. In practical breeding programs, context-specific optimization is needed to balance sequencing costs with achievable gains in prediction accuracy.

It is worth noting that in ML-based genomic prediction, feature selection serves as a critical step in screening the most informative genetic markers from vast numbers of SNPs. The hundreds of thousands to millions of SNPs generated by whole-genome resequencing or high-density chips far exceed the size of conventional training samples. Employing all such markers as input features can lead to model overfitting and imposes a significant computational burden [41]. Therefore, a common practice is to preselect SNPs potentially associated with the trait, often based on genome-wide association study (GWAS) *p*-values [42]. Alternatively, feature sets can be optimized by incorporating marker effect estimates or weighted relationship matrices [43,44], or by directly employing ranked SNP lists [45], which may also help reduce subsequent genotyping expenses.

Our results demonstrate that the predictive performance of MLP, RF, and GBLUP models generally improved with increasing SNP counts across different selection thresholds. However, the rate of improvement gradually slowed as more SNPs were added [46,47,48]. This suggests that beyond a certain density, additional markers provide diminishing genetic information and may even introduce noise, thereby potentially compromising model generalization [49,50]. Therefore, constructing a prediction model with an appropriately sized SNP set can enhance both genomic prediction accuracy and computational efficiency, facilitating the practical implementation of genomic selection in Wenshui green-shelled chicken breeding. Future studies could explore dynamic feature selection or embedded screening methods [51,52], for example, L1 regularization-based (Lasso) compressed estimation, or attention mechanisms in deep learning, to better optimize the trade-off between marker composition and predictive performance. These methods are prone to overfitting or yield unreliable results in small-sample settings. However, with large samples, they can adaptively learn optimal marker combinations, thereby improving predictive accuracy. Moreover, through model interpretability analysis, they enable the identification of key biological pathways, allowing genomic prediction models to achieve both strong predictive performance and biological interpretability.

This study has several limitations. First, the training sample size was limited to 834 individuals, which may have constrained the full utilization of information from whole-genome sequencing data. Small samples are particularly prone to overfitting and inaccurate effect estimation when estimating low-frequency allele effects or capturing complex interaction patterns. Secondly, although this study systematically calculated the prediction bias for each model, some machine learning models, such as RF for ESI-30W (b_1_ = 1.20) and MLP for EW-30W (b_1_ = 0.92), exhibited varying degrees of bias, affecting the reliability of absolute breeding value estimates. Future research should consider integrating bias correction methods to further optimize model performance. Third, the hyperparameters for the RF and MLP models were set empirically rather than through systematic optimization, which may have limited their performance potential. Finally, the evaluation relied solely on cross-validation without independent external validation, leaving the generalizability and practical applicability of the models to be further tested. To address the issue of limited sample size, genotype imputation offers an effective solution. Using the 834 sequenced individuals from this study as a reference panel, imputation can be performed for additional individuals genotyped only with low-density chips, such as the 50K chip. This approach substantially increases the sample size of high-density genotype data while controlling sequencing costs, thereby potentially improving the accuracy and stability of genomic predictions. Future research should address these limitations by expanding sample size, incorporating bias assessment, optimizing model parameters, and conducting external validation.

## 5. Conclusions

Our findings indicate that the predictive advantages of different models in genomic selection for WLGS laying hens vary with specific conditions. RF performed best for egg production and certain egg quality traits, whereas GBLUP was more stable for high-heritability traits such as egg weight and under low SNP density conditions. Both MLP and RF showed substantially improved predictive performance at high marker densities. Furthermore, whole-genome resequencing data consistently improved prediction accuracy across all models compared to 50K chip data. Accurate prediction of key economic traits, including eggshell strength and egg production, substantially enhances the precision of selecting superior breeding stock. This improvement reduces egg breakage rates and increases laying performance, thereby supporting steady gains in farming profitability within a short timeframe. These results indicate that integrating high-density genomic data with flexible machine learning algorithms can effectively optimize genetic evaluation for complex traits, thereby providing valuable support for designing efficient and precise breeding strategies in layer chickens.

## Figures and Tables

**Table 1:** Descriptive statistics of phenotypic traits in Wenshui Luhua Green-Shelled laying hens.

Trait Category	Trait	Unit	Number of Individuals	Max	Min	Median	Average	Standard Deviation
Egg production	EN	eggs	814	80	17	60	57.346	11.802
Egg quality	EW-30W	g	789	58.901	34.876	45.933	46.148	3.252
	ESI-30W	-	772	1.471	1.172	1.300	1.302	0.044
	EW-40W	g	772	67.920	37.410	49.180	49.357	3.836
	EHD-40W	mm	772	47.520	36.910	40.763	40.787	1.246
	ESI-40W	-	772	1.520	1.111	1.310	1.314	0.058
	EST-40W	mm	772	0.388	0.223	0.307	0.307	0.267
	ESS-40W	N/mm^2^	772	51.410	13.060	38.310	37.529	7.761

EN: Egg number; EW-30W: Egg weight at 30 weeks; ESI-30W: Egg shape index at 30 weeks; EW-40W: Egg weight at 40 weeks; EHD-40W: Eggshell hardness at 40 weeks; ESI-40W: Egg shape index at 40 weeks; EST-40W: Eggshell thickness at 40 weeks; ESS-40W: Eggshell strength at 40 weeks.

**Table 2:** Genetic parameters for target traits in Wenshui Luhua Green-Shelled laying hens.

Trait Category	Trait	Unit	Phenotypic Variance	Additive Variance	Residual Variance	Overall Average	Standard Error	Heritability (h2)
Egg quality	EW-30W	g	10.569	6.671	3.925	46.193	0.073	0.631
	EW-40W	g	14.926	8.996	5.930	49.378	0.091	0.603
	EHD-40W	mm	1.598	0.911	0.687	40.790	0.031	0.570
	ESI-40W	-	0.003	0.001	0.002	1.314	0.002	0.333
	ESI-30W	-	0.003	0.001	0.002	1.300	0.001	0.319
	ESS-40W	N/mm^2^	61.559	14.021	47.538	37.427	0.251	0.228
	EST-40W	mm	7.094	1.561	5.533	30.681	0.086	0.220
Egg production	EN	eggs	135.027	44.096	90.931	57.323	0.336	0.327

EN: Egg number; EW-30W: Egg weight at 30 weeks; ESI-30W: Egg shape index at 30 weeks; EW-40W: Egg weight at 40 weeks; EHD-40W: Eggshell hardness at 40 weeks; ESI-40W: Egg shape index at 40 weeks; EST-40W: Eggshell thickness at 40 weeks; ESS-40W: Eggshell strength at 40 weeks.

**Table 3:** Prediction bias for eight traits across three models evaluated by 10-fold CV.

Trait Category	Trait	Unit	GBLUP	MLP	RF
Egg production	EN	eggs	0.98	1.10	1.06
Egg quality	EW-30W	g	1.02	0.92	0.88
	ESI-30W	-	1.05	0.96	1.20
	EW-40W	g	0.99	0.89	0.93
	EHD-40W	mm	1.03	0.94	1.14
	ESI-40W	-	1.01	0.97	1.08
	EST-40W	mm	0.97	0.93	0.96
	ESS-40W	N/mm^2^	0.96	1.09	1.17

EN: Egg number; EW-30W: Egg weight at 30 weeks; ESI-30W: Egg shape index at 30 weeks; EW-40W: Egg weight at 40 weeks; EHD-40W: Eggshell hardness at 40 weeks; ESI-40W: Egg shape index at 40 weeks; EST-40W: Eggshell thickness at 40 weeks; ESS-40W: Eggshell strength at 40 weeks.

## Data Availability

All data generated or analyzed during this study are included in this published article and its Supplementary Files.

## Supplementary Materials

The following supporting information can be downloaded at https://www.mdpi.com/article/10.3390/genes17030315/s1. Table S1: Descriptive statistics of some individual raw sequencing genotype data; Table S2: Some individual sequencing genotype matching data statistics; Table S3: Descriptive statistics of map rate, error rate, and sequencing depth of some individual sequencing data. Table S4. Genetic parameters and optimal prediction accuracy of three methods for different traits. Table S5. The number of SNPs in each chromosome before and after quality control of whole genome resequencing data in Wenshui green-shelled layers. Table S6. Sequencing quality metrics for 834 Wenshui Green-Shelled(WLGS) Laying Hens.

## Abbreviations

The following abbreviations are used in this manuscript:

ML	Machine learning
MLP	Multi-layer perceptron
RF	Random forest
GBLUP	Genomic best linear unbiased prediction
GEBVs	Genomic estimated breeding values
GS	Genomic selection
SVM	Support Vector Machines
GBDT	Gradient Boosting Trees
XGBoost	Extreme Gradient Boosting
SNP	Single nucleotide polymorphism
MAF	Minor allele frequency
HWE	Hardy–Weinberg equilibrium
GWAS	Genome-wide association study
