# Supplementary material for: Comparative Accuracy of Machine Learning and GBLUP for Predicting Genomic Estimated Breeding Values in Chickens
Source: Genes (Basel). 2026 Mar 12;17(3):315. doi: 10.3390/genes17030315 (PMC13026392; doi:10.3390/genes17030315)
Supplement: Supplementary file 1 [file genes-17-00315-s001.zip › genes-4165581-supplementary.pdf]

# Supplementary Materials

**Table S1.** Descriptive statistics of some individual raw sequencing genotype data.

| ID | reads num of raw data | bases num of raw data | Q20 | Q30 | GC |
|---|---|---|---|---|---|
| D2010027459 | 47,233,371 | 7,085,005,650 | 96.15% | 88.83% | 41.54% |
| D2010027460 | 47,547,853 | 7,132,177,950 | 96.32% | 89.34% | 41.43% |
| D2010027461 | 43,788,671 | 6,568,300,650 | 96.96% | 90.89% | 41.70% |
| D2010027462 | 42,484,387 | 6,372,658,050 | 97.04% | 91.10% | 41.48% |
| D2010027463 | 41,355,897 | 6,203,384,550 | 97.06% | 91.16% | 41.35% |
| D2010027464 | 39,365,742 | 5,904,861,300 | 97.16% | 91.33% | 41.63% |
| D2010027465 | 36,485,484 | 5,472,822,600 | 96.99% | 90.96% | 41.69% |
| D2010027466 | 37,449,342 | 5,617,401,300 | 97.05% | 91.01% | 41.31% |
| D2010027467 | 40,190,086 | 6,028,512,900 | 97.12% | 91.22% | 41.68% |
| D2010027468 | 42,699,896 | 6,404,984,400 | 97.20% | 91.39% | 41.67% |
| D2010027469 | 48,546,450 | 7,281,967,500 | 96.35% | 89.36% | 41.35% |
| D2010027470 | 49,411,844 | 7,411,776,600 | 96.43% | 89.59% | 41.57% |
| D2010027471 | 52,341,164 | 7,851,174,600 | 96.62% | 90.10% | 41.53% |
| D2010027472 | 43,898,587 | 6,584,788,050 | 96.05% | 88.55% | 41.21% |
| D2010027473 | 46,034,000 | 6,905,100,000 | 96.40% | 89.53% | 41.24% |
| D2010027474 | 46,909,561 | 7,036,434,150 | 96.51% | 89.86% | 41.66% |
| D2010027475 | 50,777,809 | 7,616,671,350 | 96.42% | 89.59% | 41.55% |
| D2010027476 | 51,424,701 | 7,713,705,150 | 96.59% | 90.05% | 41.63% |
| D2010027478 | 28,775,661 | 4,316,349,150 | 95.49% | 88.10% | 41.43% |
| D2010027479 | 28,118,408 | 4,217,761,200 | 95.46% | 87.98% | 41.18% |

**Table S2.** Some individual sequencing genotype matching data statistics

| ID | reads num of map data | bases num of map data | number of mapped paired reads | median insert size | mean mapping quality | GC |
|---|---|---|---|---|---|---|
| D2010027459 | 90,187,455 | 13,036,951,195 | 90,144,165 | 347 | 34.683 | 41.42% |
| D2010027460 | 91,377,254 | 13,208,394,769 | 91,336,938 | 324 | 34.8385 | 41.27% |
| D2010027461 | 86,086,221 | 12,566,556,301 | 86,035,796 | 328 | 34.5418 | 41.58% |
| D2010027462 | 83,587,675 | 12,212,896,619 | 83,534,022 | 322 | 34.5621 | 41.38% |
| D2010027463 | 81,451,065 | 11,914,793,646 | 81,399,489 | 330 | 34.629 | 41.24% |
| D2010027464 | 77,598,374 | 11,350,761,670 | 77,539,236 | 333 | 34.5273 | 41.51% |
| D2010027465 | 71,828,123 | 10,492,802,604 | 71,777,268 | 336 | 34.6352 | 41.57% |
| D2010027466 | 73,795,579 | 10,780,062,151 | 73,758,455 | 329 | 34.6031 | 41.19% |
| D2010027467 | 79,183,427 | 11,574,151,047 | 79,133,707 | 329 | 34.645 | 41.57% |
| D2010027468 | 84,116,081 | 12,306,691,350 | 84,062,215 | 325 | 34.6559 | 41.56% |
| D2010027469 | 93,230,509 | 13,463,083,236 | 93,185,587 | 333 | 34.6849 | 41.18% |
| D2010027470 | 95,096,037 | 13,789,525,167 | 95,047,186 | 332 | 34.7856 | 41.44% |
| D2010027471 | 101,166,987 | 14,693,218,876 | 101,119,043 | 334 | 34.7057 | 41.39% |
| D2010027472 | 84,009,866 | 12,134,705,706 | 83,970,458 | 339 | 35.035 | 41.08% |
| D2010027473 | 88,807,243 | 12,884,583,026 | 88,766,348 | 343 | 34.7461 | 41.13% |
| D2010027474 | 90,562,443 | 13,148,864,533 | 90,518,360 | 329 | 34.62 | 41.54% |
| D2010027475 | 97,740,534 | 14,150,858,457 | 97,696,612 | 329 | 34.9548 | 41.41% |
| D2010027476 | 99,463,488 | 14,422,622,051 | 99,419,953 | 327 | 34.8359 | 41.49% |
| D2010027478 | 54,216,752 | 7,812,239,146 | 54,189,819 | 339 | 34.4478 | 41.29% |
| D2010027479 | 53,533,398 | 7,701,283,164 | 53,504,175 | 346 | 34.6542 | 41.03% |

**Table S3.** Descriptive statistics of map rate, error rate, and sequencing depth of some individual sequencing data.

| ID | map rate | error rate | depth | Coverage (1X) | Coverage (5X) | Coverage (10X) | Coverage (15X) |
|---|---|---|---|---|---|---|---|
| D2010027459 | 99.82% | 1.12% | 12.2371X | 96.33% | 91.29% | 71.14% | 32.03% |
| D2010027460 | 99.83% | 1.12% | 12.398X | 96.29% | 91.32% | 71.72% | 33.61% |
| D2010027461 | 99.81% | 1.10% | 11.7955X | 96.25% | 90.71% | 68.03% | 27.86% |
| D2010027462 | 99.79% | 1.08% | 11.4636X | 96.25% | 90.27% | 65.67% | 25.43% |
| D2010027463 | 99.79% | 1.08% | 11.1838X | 96.13% | 89.81% | 63.73% | 23.08% |
| D2010027464 | 99.78% | 1.08% | 10.6543X | 96.21% | 89.26% | 59.53% | 18.54% |
| D2010027465 | 99.78% | 1.10% | 9.849X | 96.03% | 87.63% | 52.39% | 13.08% |
| D2010027466 | 99.81% | 1.09% | 10.1187X | 96.07% | 87.96% | 54.86% | 15.48% |
| D2010027467 | 99.80% | 1.09% | 10.864X | 96.17% | 89.56% | 61.29% | 20.20% |
| D2010027468 | 99.78% | 1.07% | 11.5516X | 96.25% | 90.51% | 66.52% | 25.98% |
| D2010027469 | 99.82% | 1.11% | 12.6371X | 96.32% | 91.45% | 72.89% | 35.96% |
| D2010027470 | 99.82% | 1.11% | 12.9435X | 96.38% | 91.89% | 74.68% | 38.05% |
| D2010027471 | 99.82% | 1.09% | 13.7917X | 96.47% | 92.58% | 78.41% | 45.40% |
| D2010027472 | 99.81% | 1.13% | 11.3902X | 96.14% | 89.91% | 65.41% | 25.68% |
| D2010027473 | 99.84% | 1.10% | 12.0941X | 96.24% | 90.94% | 70.34% | 31.63% |
| D2010027474 | 99.81% | 1.11% | 12.3421X | 96.45% | 91.61% | 71.57% | 32.23% |
| D2010027475 | 99.83% | 1.10% | 13.2826X | 96.38% | 92.12% | 76.29% | 41.21% |
| D2010027476 | 99.84% | 1.09% | 13.5377X | 96.46% | 92.36% | 77.42% | 43.34% |
| D2010027478 | 99.83% | 1.33% | 7.3329X | 95.38% | 77.32% | 26.14% | 2.89% |
| D2010027479 | 99.80% | 1.34% | 7.2288X | 95.27% | 76.55% | 25.21% | 2.72% |

**Table S4. Genetic parameters and optimal prediction accuracy of three methods for different traits**

| Trait | Heritability | GBLUP Best Accuracy (Density) | MLP Best Accuracy (Density) | RF Best Accuracy (Density) | Best Performing Method | Conditions for Outperforming GBLUP[1] |
|---|---|---|---|---|---|---|
| EN | 0.327 | 0.213 (100K) | 0.279 (100K) | 0.378 (100K) | RF | RF (≥90K), MLP (≥90K) outperform GBLUP |
| EW-30W | 0.631 | 0.339 (100K) | 0.365 (100K) | 0.238 (100K) | MLP | MLP outperforms GBLUP at 100K |
| ESI-30W | 0.319 | 0.166 (100K) | 0.168 (100K) | 0.395 (100K) | RF | RF (≥70K) outperforms GBLUP |
| EW-40W | 0.603 | 0.381 (100K) | 0.221 (100K) | 0.227 (100K) | GBLUP | GBLUP is optimal across all densities |
| EHD-40W | 0.570 | 0.317 (90K) | 0.176 (100K) | 0.316 (100K) | GBLUP | GBLUP slightly outperforms RF; RF approaches GBLUP at ≥90K |
| ESI-40W | 0.333 | 0.289 (100K) | 0.193 (100K) | 0.211 (100K) | GBLUP | GBLUP is optimal across all densities |
| EST-40W | 0.220 | 0.176 (≥60K) | 0.165 (80K/90K) | 0.213 (100K) | RF | RF (≥90K) outperforms GBLUP |
| ESS-40W | 0.228 | 0.171 (100K) | 0.200 (100K) | 0.262 (100K) | RF | RF (≥60K), MLP (≥70K) outperform GBLUP |

EN: Egg number; EW-30W: Egg weight at 30 weeks; ESI-30W: Egg shape index at 30 weeks; EW-40W: Egg weight at 40 weeks; EHD-40W: Eggshell hardness at 40 weeks; ESI-40W: Egg shape index at 40 weeks; EST-40W: Eggshell thickness at 40 weeks; ESS-40W: Eggshell strength at 40 weeks.

[1] Conditions under which the prediction accuracy of the method begins to exceed that of GBLUP at the corresponding density, based on the provided data points from 500 to 100K.

Table S5.The number of SNPs in each chromosome before and after quality control of whole genome resequencing data in Wenshui green-shelled layers.

| Chr | Before quality control | After quality control |
|---|---|---|
| Chr1 | 3480762 | 2162452 |
| Chr2 | 2569182 | 1628370 |
| Chr3 | 1877408 | 1207789 |
| Chr4 | 1605184 | 1023959 |
| Chr5 | 1033844 | 680689 |
| Chr6 | 688443 | 436897 |
| Chr7 | 637938 | 416837 |
| Chr8 | 486303 | 310988 |
| Chr9 | 422150 | 271370 |
| Chr10 | 362171 | 226591 |
| Chr11 | 310114 | 190791 |
| Chr12 | 371283 | 227502 |
| Chr13 | 337397 | 204671 |
| Chr14 | 298702 | 174379 |
| Chr15 | 211438 | 125254 |
| Chr16 | 116365 | 10331 |
| Chr17 | 178975 | 103752 |
| Chr18 | 222109 | 116356 |
| Chr19 | 169712 | 99603 |
| Chr20 | 237055 | 152294 |
| Chr21 | 120300 | 73152 |
| Chr22 | 63752 | 23717 |
| Chr23 | 119706 | 60492 |
| Chr24 | 124822 | 67868 |
| Chr25 | 87224 | 17095 |
| Chr26 | 113333 | 55444 |
| Chr27 | 224308 | 50821 |
| Chr28 | 102451 | 43064 |

| | | |
|---|---|---|
| **Chr30** | **35007** | **2105** |
| **Chr31** | **257886** | **12299** |
| **Chr32** | **12032** | **701** |
| **Chr33** | **422190** | **43776** |
| **All** | **17299546** | **10221409** |

Table S6. Sequencing quality metrics for 834 Wenshui Green-Shelled(WLGS) Laying Hens.

| ID | reads num of map data | bases num of map data | number of mapped paired reads |
|---|---|---|---|
| D2010027186 | 112,251,276 | 16,322,841,805 | 112,198,406 |
| D2010027187 | 104,806,421 | 15,356,445,538 | 104,738,613 |
| D2010027188 | 113,530,702 | 16,620,740,164 | 113,465,840 |
| D2010027189 | 108,124,874 | 15,824,410,610 | 108,059,566 |
| D2010027190 | 105,440,931 | 15,458,661,632 | 105,377,349 |
| D2010027191 | 113,245,926 | 16,621,608,842 | 113,185,305 |
| D2010027192 | 120,855,100 | 17,684,891,361 | 120,789,378 |
| D2010027193 | 107,526,773 | 15,694,402,118 | 107,460,687 |
| D2010027194 | 109,094,039 | 15,922,786,715 | 109,029,500 |
| D2010027195 | 100,501,208 | 14,681,784,680 | 100,439,703 |
| D2010027196 | 108,695,188 | 15,919,015,421 | 108,636,459 |
| D2010027197 | 97,700,823 | 14,288,296,371 | 97,634,557 |
| D2010027198 | 101,876,146 | 14,889,170,301 | 101,809,822 |
| D2010027199 | 109,153,557 | 15,941,666,670 | 109,101,843 |
| D2010027200 | 106,686,741 | 15,593,587,939 | 106,622,740 |
| D2010027201 | 117,782,763 | 17,204,871,014 | 117,709,100 |
| D2010027202 | 116,385,953 | 17,008,973,074 | 116,314,736 |
| D2010027203 | 102,692,376 | 15,026,478,954 | 102,617,231 |
| D2010027204 | 104,947,897 | 15,369,714,115 | 104,887,005 |
| D2010027205 | 98,714,250 | 14,414,550,331 | 98,652,738 |
| D2010027206 | 108,578,735 | 15,915,230,860 | 108,509,929 |
| D2010027207 | 105,307,509 | 15,442,791,209 | 105,243,098 |
| D2010027208 | 115,960,382 | 16,960,932,907 | 115,896,054 |
| D2010027209 | 115,815,933 | 16,946,607,831 | 115,752,436 |
| D2010027210 | 104,751,279 | 15,275,861,094 | 104,684,190 |
| D2010027211 | 107,135,049 | 15,652,880,382 | 107,075,799 |
| D2010027212 | 115,319,913 | 16,858,244,224 | 115,251,547 |
| D2010027213 | 96,838,222 | 14,133,090,825 | 96,778,350 |
| D2010027214 | 94,513,867 | 13,862,732,373 | 94,451,341 |
| D2010027215 | 100,167,158 | 14,663,217,359 | 100,106,676 |
| D2010027216 | 113,106,291 | 16,571,552,770 | 113,039,059 |
| D2010027217 | 105,947,069 | 15,442,376,247 | 105,889,628 |
| D2010027218 | 113,364,347 | 16,527,508,195 | 113,297,251 |
| D2010027219 | 103,495,874 | 15,137,692,110 | 103,429,673 |
| D2010027220 | 105,384,496 | 15,394,229,207 | 105,318,297 |
| D2010027221 | 102,891,757 | 15,018,739,821 | 102,821,754 |
| D2010027222 | 100,041,995 | 14,592,534,703 | 99,981,714 |
| D2010027223 | 112,828,512 | 16,512,096,891 | 112,763,409 |
| D2010027224 | 89,483,309 | 13,087,117,319 | 89,432,927 |
| D2010027225 | 110,743,640 | 16,202,225,941 | 110,672,172 |
| D2010027477 | 49,541,415 | 7,091,663,002 | 49,513,100 |
| D2010027488 | 61,228,354 | 8,943,881,380 | 61,187,057 |
| D2010027489 | 60,730,209 | 8,862,258,201 | 60,690,585 |
| D2010027490 | 77,370,101 | 11,309,254,420 | 77,321,284 |
| D2010027491 | 52,061,019 | 7,602,544,928 | 52,028,634 |
| D2010027492 | 65,630,221 | 9,599,696,574 | 65,583,263 |
| D2010027493 | 73,699,781 | 10,748,688,529 | 73,660,965 |
| D2010027494 | 81,233,829 | 11,859,605,560 | 81,189,002 |
| D2010027495 | 87,182,686 | 12,746,972,138 | 87,137,849 |
| D2010027496 | 78,997,231 | 11,532,254,966 | 78,953,573 |
| D2010027497 | 75,870,653 | 11,068,601,046 | 75,829,257 |

| | | | |
|---|---|---|---|
| D2010027498 | 81,516,169 | 11,894,829,055 | 81,474,541 |
| D2010027499 | 80,441,298 | 11,729,167,366 | 80,397,882 |
| D2010027500 | 90,166,135 | 13,159,972,044 | 90,116,341 |
| D2010027501 | 90,552,835 | 13,208,937,884 | 90,502,231 |
| D2010027502 | 70,385,322 | 10,248,473,181 | 70,347,180 |
| D2010027503 | 90,213,701 | 13,151,601,061 | 90,164,651 |
| D2010027504 | 78,878,205 | 11,510,912,953 | 78,834,996 |
| D2010027505 | 117,742,795 | 17,272,239,337 | 117,681,765 |
| D2010027506 | 132,148,916 | 19,395,056,619 | 132,086,829 |
| D2010027507 | 136,478,123 | 20,016,717,484 | 136,413,060 |
| D2010027508 | 125,794,377 | 18,429,987,933 | 125,728,818 |
| D2010027509 | 106,373,010 | 15,492,914,475 | 106,320,391 |
| D2010027510 | 119,968,776 | 17,552,606,504 | 119,911,445 |
| D2010027511 | 115,944,969 | 16,919,033,227 | 115,889,247 |
| D2010027512 | 101,794,441 | 14,886,826,351 | 101,742,938 |
| D2010027513 | 104,930,437 | 15,369,517,727 | 104,874,024 |
| D2010027514 | 127,739,900 | 18,745,992,406 | 127,670,144 |
| D2010027515 | 114,184,802 | 16,692,807,232 | 114,124,348 |
| D2010027516 | 110,922,265 | 16,205,820,168 | 110,863,292 |
| D2010027517 | 101,570,671 | 14,827,674,484 | 101,520,003 |
| D2010027518 | 122,803,736 | 17,976,533,382 | 122,737,360 |
| D2010027519 | 112,083,353 | 16,383,501,692 | 112,023,057 |
| D2010027520 | 102,231,696 | 14,946,506,910 | 102,180,315 |
| D2010027521 | 101,413,501 | 14,833,941,451 | 101,362,506 |
| D2010027522 | 118,802,896 | 17,390,332,914 | 118,739,105 |
| D2010027523 | 112,395,309 | 16,447,679,356 | 112,337,772 |
| D2010027524 | 122,628,820 | 17,970,738,870 | 122,565,164 |
| D2010027525 | 81,813,841 | 11,933,932,701 | 81,771,099 |
| D2010027526 | 82,035,870 | 11,977,875,546 | 81,994,044 |
| D2010027774 | 183,116,455 | 26,877,817,669 | 183,019,640 |
| D2010027775 | 196,562,210 | 28,880,242,087 | 196,463,149 |
| D2010027776 | 59,295,138 | 8,775,763,095 | 59,264,837 |
| D2010027777 | 62,069,792 | 9,189,883,240 | 62,035,097 |
| D2010027778 | 51,886,715 | 7,672,900,840 | 51,856,842 |
| D2010027779 | 54,561,103 | 8,074,582,626 | 54,528,717 |
| D2010027780 | 59,427,569 | 8,804,991,644 | 59,392,433 |
| D2010027781 | 68,927,331 | 10,216,370,895 | 68,890,949 |
| D2010027782 | 63,844,166 | 9,459,770,423 | 63,809,946 |
| D2010027783 | 68,844,422 | 10,188,345,352 | 68,802,543 |
| D2010027784 | 69,419,330 | 10,292,644,101 | 69,377,329 |
| D2010027785 | 60,364,212 | 8,947,211,857 | 60,325,903 |
| D2010027786 | 59,275,260 | 8,776,142,803 | 59,236,980 |
| D2010027787 | 42,514,249 | 6,288,779,743 | 42,487,262 |
| D2010027788 | 56,883,682 | 8,406,385,030 | 56,851,563 |
| D2010027789 | 81,640,654 | 12,100,560,741 | 81,594,638 |
| D2010027791 | 65,934,382 | 9,770,063,360 | 65,893,693 |
| D2010027792 | 80,719,076 | 11,953,104,482 | 80,672,593 |
| D2010027793 | 72,946,385 | 10,801,775,245 | 72,904,055 |
| D2010027794 | 67,381,511 | 9,974,790,381 | 67,343,773 |
| D2010027795 | 63,669,955 | 9,427,849,732 | 63,634,053 |
| D2010027796 | 59,171,615 | 8,758,387,007 | 59,137,352 |
| D2010027797 | 81,547,405 | 11,811,653,711 | 81,511,800 |

| | | | |
|---|---|---|---|
| D2010027798 | 76,048,603 | 10,955,370,874 | 76,015,216 |
| D2010027799 | 94,310,988 | 13,679,521,084 | 94,270,347 |
| D2010027800 | 96,395,720 | 13,989,322,290 | 96,355,123 |
| D2010027801 | 105,972,353 | 15,382,686,105 | 105,933,837 |
| D2010027802 | 87,949,122 | 12,722,818,759 | 87,913,555 |
| D2010027803 | 79,915,385 | 11,512,824,428 | 79,878,665 |
| D2010027804 | 83,904,449 | 12,112,322,199 | 83,866,128 |
| D2010027805 | 82,505,299 | 11,926,819,027 | 82,468,835 |
| D2010027806 | 93,607,351 | 13,541,303,215 | 93,568,426 |
| D2010027807 | 105,451,173 | 15,266,910,391 | 105,408,751 |
| D2010027808 | 98,713,572 | 14,254,385,442 | 98,674,544 |
| D2010027809 | 94,507,732 | 13,667,332,020 | 94,468,737 |
| D2010027810 | 83,698,009 | 12,092,206,195 | 83,665,448 |
| D2010027811 | 81,205,729 | 11,717,972,572 | 81,171,439 |
| D2010027812 | 73,614,531 | 10,617,950,311 | 73,581,731 |
| D2010027813 | 94,587,256 | 13,659,034,775 | 94,547,561 |
| D2010027814 | 83,490,022 | 11,984,044,061 | 83,454,082 |
| D2010027815 | 84,657,462 | 12,203,413,932 | 84,620,348 |
| D2010027816 | 92,945,970 | 13,426,407,479 | 92,904,875 |
| D2010027817 | 58,734,643 | 8,613,761,399 | 58,694,358 |
| D2010027818 | 45,186,088 | 6,625,715,916 | 45,142,711 |
| D2010027819 | 60,512,563 | 8,888,113,732 | 60,470,691 |
| D2010027820 | 43,266,466 | 6,326,175,856 | 43,231,885 |
| D2010027821 | 51,354,040 | 7,514,912,034 | 51,313,762 |
| D2010027822 | 56,286,390 | 8,250,564,124 | 56,241,033 |
| D2010027823 | 50,602,755 | 7,395,845,470 | 50,567,451 |
| D2010027824 | 62,531,927 | 9,148,280,191 | 62,491,640 |
| D2010027825 | 59,552,942 | 8,723,258,922 | 59,506,465 |
| D2010027826 | 62,011,444 | 9,089,050,763 | 61,974,854 |
| D2010027827 | 64,830,539 | 9,506,680,398 | 64,784,053 |
| D2010027828 | 52,088,426 | 7,631,291,528 | 52,050,656 |
| D2010027829 | 67,148,396 | 9,875,373,463 | 67,096,641 |
| D2010027830 | 57,831,240 | 8,466,568,160 | 57,786,976 |
| D2010027831 | 77,557,801 | 11,383,736,743 | 77,508,338 |
| D2010027832 | 56,733,957 | 8,312,881,818 | 56,697,971 |
| D2010027833 | 93,474,204 | 13,519,655,017 | 93,438,538 |
| D2010027834 | 99,396,634 | 14,443,655,360 | 99,357,436 |
| D2010027835 | 89,857,053 | 12,998,576,598 | 89,819,367 |
| D2010027836 | 88,825,282 | 12,862,711,226 | 88,788,800 |
| D2010027837 | 103,187,556 | 14,994,276,670 | 103,143,202 |
| D2010027838 | 121,201,499 | 17,629,282,761 | 121,149,415 |
| D2010027839 | 97,566,742 | 14,197,851,919 | 97,521,032 |
| D2010027840 | 114,928,691 | 16,704,683,750 | 114,876,564 |
| D2010027841 | 96,728,608 | 14,098,725,059 | 96,687,949 |
| D2010027842 | 88,181,577 | 12,847,072,296 | 88,145,689 |
| D2010027843 | 102,491,786 | 14,942,924,017 | 102,445,392 |
| D2010027844 | 92,807,107 | 13,529,297,111 | 92,766,014 |
| D2010027845 | 98,605,348 | 14,266,203,745 | 98,558,996 |
| D2010027846 | 108,259,563 | 15,671,061,335 | 108,208,610 |
| D2010027847 | 104,940,880 | 15,187,596,236 | 104,892,134 |
| D2010027848 | 112,083,355 | 16,230,550,749 | 112,026,957 |
| D2010027849 | 108,687,977 | 15,884,309,696 | 108,640,679 |

| | | | |
|---|---|---|---|
| D2010027850 | 75,863,684 | 11,027,550,877 | 75,828,597 |
| D2010027851 | 100,832,727 | 14,729,806,115 | 100,787,013 |
| D2010027852 | 94,869,149 | 13,851,191,320 | 94,829,396 |
| D2010027853 | 101,018,430 | 14,693,160,258 | 100,968,846 |
| D2010027854 | 104,282,888 | 15,133,787,027 | 104,229,005 |
| D2010027855 | 109,231,304 | 15,873,949,463 | 109,179,360 |
| D2010027856 | 106,013,198 | 15,403,226,782 | 105,964,145 |
| D2010027857 | 96,373,565 | 14,040,742,215 | 96,339,613 |
| D2010027858 | 89,583,290 | 13,068,896,755 | 89,545,536 |
| D2010027859 | 89,890,263 | 13,085,500,695 | 89,852,458 |
| D2010027860 | 80,822,067 | 11,781,100,160 | 80,789,897 |
| D2010027686 | 130,998,523 | 19,228,653,869 | 130,938,969 |
| D2010027687 | 147,070,894 | 21,637,882,879 | 146,999,769 |
| D2010027688 | 126,142,747 | 18,523,694,008 | 126,082,263 |
| D2010027689 | 138,618,055 | 20,372,036,448 | 138,554,160 |
| D2010027690 | 131,387,660 | 19,313,956,256 | 131,327,077 |
| D2010027691 | 135,307,974 | 19,892,247,735 | 135,245,604 |
| D2010027692 | 135,553,579 | 19,951,907,227 | 135,489,965 |
| D2010027693 | 137,409,419 | 20,218,811,930 | 137,343,279 |
| D2010027694 | 68,182,726 | 10,099,040,267 | 68,133,098 |
| D2010027695 | 65,021,726 | 9,626,458,782 | 64,972,652 |
| D2010027697 | 68,853,337 | 10,201,914,485 | 68,798,437 |
| D2010027698 | 46,401,435 | 6,871,768,828 | 46,367,244 |
| D2010027699 | 56,801,305 | 8,308,698,309 | 56,773,248 |
| D2010027700 | 81,628,496 | 12,003,867,912 | 81,588,117 |
| D2010027701 | 65,423,675 | 9,601,530,277 | 65,391,579 |
| D2010027702 | 71,249,090 | 10,449,755,780 | 71,214,895 |
| D2010027703 | 73,872,251 | 10,847,217,335 | 73,833,620 |
| D2010027704 | 64,925,019 | 9,533,632,835 | 64,891,385 |
| D2010027705 | 64,344,342 | 9,442,554,627 | 64,312,666 |
| D2010027706 | 50,740,093 | 7,439,984,885 | 50,712,125 |
| D2010027707 | 64,495,192 | 9,542,843,302 | 64,441,171 |
| D2010027708 | 71,656,070 | 10,607,844,944 | 71,600,930 |
| D2010027709 | 68,570,585 | 10,147,793,819 | 68,521,685 |
| D2010027710 | 66,738,519 | 9,879,092,901 | 66,687,226 |
| D2010027711 | 144,185,934 | 21,218,634,861 | 144,122,887 |
| D2010027712 | 132,954,981 | 19,574,324,411 | 132,878,056 |
| D2010027713 | 137,667,451 | 20,269,132,426 | 137,599,050 |
| D2010027714 | 130,825,990 | 19,242,298,316 | 130,753,797 |
| D2010027715 | 65,817,723 | 9,733,036,967 | 65,751,286 |
| D2010027716 | 65,619,317 | 9,701,867,395 | 65,556,865 |
| D2010027717 | 67,981,661 | 10,048,876,278 | 67,918,834 |
| D2010027718 | 60,493,413 | 8,928,564,900 | 60,431,010 |
| D2010027719 | 89,461,598 | 13,046,339,993 | 89,422,830 |
| D2010027720 | 80,243,877 | 11,681,934,079 | 80,207,028 |
| D2010027721 | 93,265,018 | 13,590,504,432 | 93,222,439 |
| D2010027722 | 79,467,411 | 11,587,384,229 | 79,431,590 |
| D2010027723 | 92,545,502 | 13,499,611,639 | 92,503,264 |
| D2010027724 | 91,322,952 | 13,278,763,369 | 91,289,055 |
| D2010027725 | 90,890,585 | 13,253,418,130 | 90,851,732 |
| D2010027726 | 88,745,983 | 12,911,113,031 | 88,704,485 |
| D2010027727 | 101,090,825 | 14,650,271,684 | 101,051,458 |

| | | | |
|---|---|---|---|
| D2010027728 | 90,515,530 | 13,136,684,759 | 90,479,442 |
| D2010027729 | 94,451,023 | 13,715,712,111 | 94,414,712 |
| D2010027730 | 82,661,700 | 11,947,200,811 | 82,628,496 |
| D2010027731 | 91,583,468 | 13,298,467,856 | 91,545,412 |
| D2010027732 | 95,856,216 | 13,915,560,275 | 95,816,399 |
| D2010027733 | 94,666,859 | 13,692,888,009 | 94,627,931 |
| D2010027734 | 103,801,871 | 15,075,828,018 | 103,760,788 |
| D2010027735 | 90,554,675 | 13,123,068,003 | 90,515,293 |
| D2010027736 | 92,473,683 | 13,375,989,571 | 92,433,313 |
| D2010027737 | 90,300,284 | 13,061,922,735 | 90,262,467 |
| D2010027738 | 79,344,987 | 11,459,797,314 | 79,310,286 |
| D2010027739 | 52,590,217 | 7,616,429,906 | 52,559,776 |
| D2010027740 | 51,251,016 | 7,433,338,481 | 51,226,811 |
| D2010027741 | 54,774,464 | 7,937,000,292 | 54,746,800 |
| D2010027742 | 47,610,256 | 6,877,550,650 | 47,589,368 |
| D2010027743 | 60,730,918 | 8,986,429,258 | 60,677,898 |
| D2010027744 | 55,299,246 | 8,181,385,952 | 55,238,760 |
| D2010027745 | 50,834,504 | 7,519,824,029 | 50,776,810 |
| D2010027746 | 52,426,398 | 7,752,779,374 | 52,365,344 |
| D2010027747 | 89,933,449 | 13,073,123,015 | 89,899,699 |
| D2010027748 | 105,085,338 | 15,296,680,995 | 105,039,488 |
| D2010027749 | 74,290,565 | 10,755,288,405 | 74,258,563 |
| D2010027750 | 101,190,912 | 14,726,542,688 | 101,148,877 |
| D2010027751 | 99,689,436 | 14,381,367,704 | 99,647,306 |
| D2010027752 | 83,974,688 | 12,080,774,797 | 83,937,231 |
| D2010027753 | 78,879,669 | 11,325,709,566 | 78,842,650 |
| D2010027754 | 105,531,475 | 15,266,253,252 | 105,482,213 |
| D2010027755 | 59,684,977 | 8,834,561,389 | 59,650,408 |
| D2010027756 | 63,132,844 | 9,340,463,528 | 63,100,924 |
| D2010027757 | 60,188,753 | 8,906,782,463 | 60,155,304 |
| D2010027758 | 59,771,061 | 8,839,888,995 | 59,737,236 |
| D2010027759 | 127,867,660 | 18,534,426,855 | 127,811,828 |
| D2010027760 | 70,579,925 | 10,064,093,332 | 70,547,799 |
| D2010027761 | 87,908,052 | 12,612,179,709 | 87,868,259 |
| D2010027762 | 82,808,099 | 11,881,649,220 | 82,771,929 |
| D2010027763 | 66,687,218 | 9,871,985,758 | 66,651,256 |
| D2010027764 | 73,056,397 | 10,806,538,653 | 73,013,626 |
| D2010027765 | 70,645,143 | 10,451,294,423 | 70,605,885 |
| D2010027766 | 70,476,331 | 10,424,774,077 | 70,438,746 |
| D2010027767 | 88,256,514 | 12,650,016,026 | 88,219,714 |
| D2010027768 | 98,056,716 | 14,167,549,300 | 98,013,536 |
| D2010027770 | 78,941,339 | 11,296,609,133 | 78,906,854 |
| D2010027771 | 82,814,524 | 11,926,874,533 | 82,776,142 |
| D2010027772 | 212,089,118 | 31,185,964,680 | 211,978,288 |
| D2010027773 | 203,844,624 | 29,990,817,205 | 203,730,796 |
| D2010027527 | 87,315,498 | 12,746,737,281 | 87,269,155 |
| D2010027528 | 109,151,643 | 15,838,389,259 | 109,098,344 |
| D2010027529 | 54,027,105 | 7,887,517,263 | 53,998,623 |
| D2010027530 | 61,933,411 | 9,028,301,103 | 61,899,967 |
| D2010027531 | 118,033,634 | 17,172,875,756 | 117,980,347 |
| D2010027532 | 80,552,166 | 11,785,322,020 | 80,504,795 |
| D2010027533 | 73,022,563 | 10,660,809,576 | 72,982,668 |

| | | | |
|---|---|---|---|
| D2010027534 | 80,988,877 | 11,759,626,155 | 80,952,207 |
| D2010027535 | 79,156,946 | 11,565,429,505 | 79,113,844 |
| D2010027536 | 85,903,039 | 12,522,467,235 | 85,855,494 |
| D2010027537 | 74,983,143 | 10,932,964,702 | 74,942,545 |
| D2010027538 | 89,044,922 | 12,813,457,242 | 89,008,699 |
| D2010027540 | 81,645,956 | 11,917,040,747 | 81,601,009 |
| D2010027541 | 66,370,754 | 9,678,183,567 | 66,333,616 |
| D2010027542 | 109,527,648 | 15,904,045,328 | 109,481,614 |
| D2010027543 | 61,110,061 | 8,927,881,819 | 61,076,673 |
| D2010027544 | 73,705,291 | 10,765,501,467 | 73,666,550 |
| D2010027545 | 72,077,606 | 10,525,324,193 | 72,038,127 |
| D2010027546 | 90,444,602 | 13,073,611,934 | 90,407,862 |
| D2010027547 | 77,754,927 | 11,352,782,622 | 77,712,500 |
| D2010027548 | 94,571,479 | 13,664,640,350 | 94,524,862 |
| D2010027549 | 98,651,470 | 14,291,539,993 | 98,605,143 |
| D2010027550 | 94,945,295 | 13,698,209,857 | 94,898,329 |
| D2010027551 | 99,900,252 | 14,461,520,001 | 99,851,751 |
| D2010027552 | 117,502,742 | 17,090,981,771 | 117,449,742 |
| D2010027553 | 77,601,383 | 11,188,192,858 | 77,561,776 |
| D2010027554 | 62,186,667 | 8,901,430,100 | 62,156,344 |
| D2010027555 | 70,265,034 | 10,030,410,777 | 70,234,323 |
| D2010027556 | 107,819,220 | 15,627,818,321 | 107,769,864 |
| D2010027557 | 83,911,379 | 12,043,551,032 | 83,873,882 |
| D2010027558 | 77,757,650 | 11,129,698,246 | 77,725,222 |
| D2010027559 | 105,050,698 | 15,264,579,175 | 105,001,365 |
| D2010027560 | 90,071,296 | 12,929,924,775 | 90,027,415 |
| D2010027561 | 95,766,562 | 13,888,687,843 | 95,724,486 |
| D2010027562 | 97,098,781 | 13,980,229,076 | 97,048,655 |
| D2010027563 | 94,502,266 | 13,579,976,332 | 94,453,897 |
| D2010027564 | 112,523,967 | 16,389,238,914 | 112,472,499 |
| D2010027565 | 86,941,755 | 12,470,564,678 | 86,896,419 |
| D2010027566 | 58,345,249 | 8,585,851,020 | 58,286,756 |
| D2010027567 | 109,239,439 | 15,874,169,636 | 109,191,310 |
| D2010027568 | 76,722,879 | 11,311,702,855 | 76,645,403 |
| D2010027569 | 56,540,587 | 8,334,460,293 | 56,488,682 |
| D2010027570 | 108,096,648 | 15,677,500,560 | 108,044,658 |
| D2010027571 | 67,738,862 | 9,978,047,695 | 67,659,557 |
| D2010027572 | 57,217,342 | 8,417,288,267 | 57,183,849 |
| D2010027573 | 62,209,080 | 9,186,149,703 | 62,172,158 |
| D2010027574 | 109,021,660 | 15,817,791,465 | 108,971,040 |
| D2010027575 | 71,969,013 | 10,627,088,967 | 71,930,441 |
| D2010027576 | 65,077,687 | 9,616,569,693 | 65,038,105 |
| D2010027577 | 102,807,174 | 15,028,435,390 | 102,768,972 |
| D2010027578 | 77,215,833 | 11,018,758,349 | 77,180,591 |
| D2010027579 | 91,475,757 | 13,106,009,980 | 91,432,728 |
| D2010027580 | 89,944,644 | 13,134,135,559 | 89,904,800 |
| D2010027581 | 92,222,616 | 13,233,898,229 | 92,180,942 |
| D2010027582 | 97,387,121 | 13,961,026,483 | 97,343,095 |
| D2010027583 | 98,347,927 | 14,371,222,459 | 98,307,127 |
| D2010027584 | 90,208,462 | 13,350,911,543 | 90,130,786 |
| D2010027585 | 84,077,985 | 12,252,855,928 | 84,040,779 |
| D2010027586 | 82,870,570 | 12,265,626,949 | 82,779,706 |

| | | | |
|---|---|---|---|
| D2010027587 | 79,238,595 | 11,728,360,607 | 79,144,650 |
| D2010027588 | 135,328,760 | 19,940,168,253 | 135,255,279 |
| D2010027589 | 74,641,425 | 11,039,170,336 | 74,554,960 |
| D2010027590 | 47,003,248 | 6,958,804,244 | 46,962,103 |
| D2010027591 | 51,477,667 | 7,621,755,761 | 51,430,791 |
| D2010027592 | 145,462,815 | 21,416,421,038 | 145,385,114 |
| D2010027593 | 42,398,090 | 6,276,505,244 | 42,363,192 |
| D2010027594 | 49,530,168 | 7,330,659,067 | 49,477,649 |
| D2010027595 | 68,632,624 | 10,165,244,830 | 68,595,851 |
| D2010027596 | 60,810,931 | 9,004,644,443 | 60,775,496 |
| D2010027597 | 153,986,757 | 22,669,892,358 | 153,910,879 |
| D2010027598 | 67,738,249 | 10,030,675,344 | 67,703,638 |
| D2010027599 | 67,582,534 | 10,018,424,385 | 67,545,805 |
| D2010027600 | 65,144,819 | 9,653,225,080 | 65,108,493 |
| D2010027601 | 128,743,149 | 18,905,216,739 | 128,673,324 |
| D2010027602 | 67,783,459 | 10,034,169,615 | 67,749,308 |
| D2010027603 | 68,611,720 | 10,164,823,211 | 68,575,682 |
| D2010027604 | 66,167,277 | 9,785,839,530 | 66,129,202 |
| D2010027605 | 136,848,755 | 20,151,924,331 | 136,786,189 |
| D2010027606 | 85,244,536 | 12,617,730,832 | 85,200,926 |
| D2010027607 | 62,079,682 | 9,176,800,329 | 62,046,442 |
| D2010027608 | 75,647,142 | 11,203,170,863 | 75,603,417 |
| D2010027609 | 132,446,734 | 19,494,326,114 | 132,370,449 |
| D2010027610 | 70,742,045 | 10,476,857,106 | 70,695,617 |
| D2010027611 | 105,469,897 | 15,319,154,000 | 105,425,403 |
| D2010027612 | 103,140,775 | 14,942,607,589 | 103,092,961 |
| D2010027613 | 139,710,182 | 20,579,880,101 | 139,637,871 |
| D2010027614 | 104,623,736 | 15,204,229,530 | 104,579,592 |
| D2010027615 | 99,685,550 | 14,467,804,873 | 99,641,142 |
| D2010027616 | 115,866,156 | 17,071,149,619 | 115,787,501 |
| D2010027617 | 102,665,563 | 14,906,086,841 | 102,616,669 |
| D2010027618 | 97,927,191 | 14,219,949,071 | 97,881,271 |
| D2010027619 | 132,188,549 | 19,479,134,679 | 132,112,900 |
| D2010027620 | 96,167,264 | 13,953,299,221 | 96,119,783 |
| D2010027621 | 92,374,601 | 13,380,219,874 | 92,329,337 |
| D2010027622 | 128,899,552 | 18,945,822,327 | 128,849,044 |
| D2010027623 | 97,197,592 | 14,087,749,668 | 97,154,302 |
| D2010027624 | 107,089,009 | 15,554,308,323 | 107,046,167 |
| D2010027625 | 139,505,384 | 20,537,209,031 | 139,432,226 |
| D2010027626 | 100,480,245 | 14,544,327,246 | 100,439,070 |
| D2010027627 | 93,873,729 | 13,558,910,364 | 93,837,566 |
| D2010027628 | 112,270,847 | 16,272,326,425 | 112,222,283 |
| D2010027629 | 130,699,598 | 19,236,926,909 | 130,627,541 |
| D2010027630 | 120,338,605 | 17,462,058,261 | 120,279,902 |
| D2010027631 | 141,945,721 | 20,890,261,841 | 141,871,400 |
| D2010027632 | 106,726,136 | 15,430,363,048 | 106,675,842 |
| D2010027633 | 105,190,705 | 15,217,171,314 | 105,143,322 |
| D2010027634 | 86,851,373 | 12,491,077,812 | 86,813,615 |
| D2010027635 | 126,178,725 | 18,588,780,538 | 126,094,156 |
| D2010027636 | 99,207,721 | 14,351,384,355 | 99,165,439 |
| D2010027637 | 83,264,494 | 12,011,908,756 | 83,228,565 |
| D2010027638 | 126,690,169 | 18,647,950,061 | 126,620,170 |

| | | | |
|---|---|---|---|
| D2010027639 | 94,509,598 | 13,642,140,592 | 94,467,796 |
| D2010027640 | 114,686,777 | 16,668,303,310 | 114,636,674 |
| D2010027641 | 111,516,570 | 16,377,261,335 | 111,456,023 |
| D2010027642 | 136,676,850 | 20,151,493,599 | 136,598,849 |
| D2010027643 | 133,209,476 | 19,604,922,738 | 133,134,094 |
| D2010027644 | 144,500,189 | 21,261,737,043 | 144,429,014 |
| D2010027645 | 137,670,181 | 20,251,289,100 | 137,600,947 |
| D2010027646 | 89,898,500 | 13,188,527,527 | 89,815,846 |
| D2010027647 | 93,376,852 | 13,699,087,569 | 93,311,458 |
| D2010027648 | 94,872,979 | 13,924,088,223 | 94,796,685 |
| D2010027649 | 81,044,231 | 11,891,012,099 | 80,976,248 |
| D2010027650 | 78,490,587 | 11,448,819,430 | 78,453,282 |
| D2010027651 | 81,345,016 | 11,832,870,910 | 81,304,856 |
| D2010027652 | 97,328,000 | 14,228,136,766 | 97,282,866 |
| D2010027653 | 87,639,543 | 12,784,898,004 | 87,604,164 |
| D2010027654 | 103,144,150 | 15,136,580,165 | 103,070,981 |
| D2010027655 | 97,847,258 | 14,363,592,536 | 97,767,204 |
| D2010027656 | 93,203,650 | 13,663,127,167 | 93,121,798 |
| D2010027657 | 84,562,082 | 12,383,812,419 | 84,481,195 |
| D2010027658 | 132,555,069 | 19,499,966,585 | 132,487,825 |
| D2010027659 | 172,558,540 | 25,423,185,817 | 172,471,035 |
| D2010027660 | 134,737,464 | 19,855,451,383 | 134,669,856 |
| D2010027661 | 145,483,143 | 21,423,251,746 | 145,398,883 |
| D2010027662 | 145,070,944 | 21,348,079,457 | 145,011,219 |
| D2010027663 | 133,184,346 | 19,591,155,426 | 133,119,448 |
| D2010027664 | 147,561,314 | 21,741,396,503 | 147,495,209 |
| D2010027665 | 128,255,162 | 18,869,806,235 | 128,194,686 |
| D2010027666 | 135,640,135 | 19,957,854,548 | 135,576,803 |
| D2010027667 | 134,807,288 | 19,811,809,842 | 134,745,644 |
| D2010027668 | 147,974,752 | 21,779,587,808 | 147,913,906 |
| D2010027669 | 135,180,988 | 19,866,563,232 | 135,118,403 |
| D2010027670 | 80,261,416 | 11,769,987,156 | 80,220,993 |
| D2010027671 | 44,867,293 | 6,549,305,223 | 44,844,306 |
| D2010027672 | 56,208,816 | 8,221,031,436 | 56,179,641 |
| D2010027673 | 54,035,754 | 7,905,120,684 | 54,006,124 |
| D2010027674 | 64,353,994 | 9,438,386,823 | 64,319,426 |
| D2010027675 | 60,307,006 | 8,832,594,364 | 60,274,661 |
| D2010027676 | 67,472,720 | 9,889,940,677 | 67,439,709 |
| D2010027677 | 60,439,610 | 8,837,242,712 | 60,407,997 |
| D2010027678 | 163,230,158 | 24,025,629,180 | 163,164,677 |
| D2010027679 | 149,448,190 | 22,026,224,462 | 149,387,365 |
| D2010027680 | 143,690,000 | 21,158,410,360 | 143,633,951 |
| D2010027681 | 133,789,302 | 19,691,699,060 | 133,732,966 |
| D2010027682 | 139,996,302 | 20,630,979,428 | 139,929,705 |
| D2010027683 | 150,175,780 | 22,162,258,937 | 150,110,305 |
| D2010027684 | 156,299,414 | 23,014,538,991 | 156,229,825 |
| D2010027685 | 139,514,794 | 20,530,053,550 | 139,453,200 |
| D2010027074 | 59213227 | 8,758,958,047 | 59177019 |
| D2010027075 | 54530972 | 8,060,670,087 | 54495897 |
| D2010027076 | 50601089 | 7,482,677,394 | 50569872 |
| D2010027077 | 61712586 | 9,121,494,006 | 61672826 |
| D2010027078 | 39128865 | 5,783,997,963 | 39103125 |

| | | | |
|---|---|---|---|
| D2010027079 | 59377807 | 8,774,687,864 | 59343519 |
| D2010027080 | 66594917 | 9,836,860,612 | 66555204 |
| D2010027081 | 69961603 | 10,343,932,084 | 69917474 |
| D2010027082 | 29460388 | 4,350,842,890 | 29442690 |
| D2010027083 | 29519364 | 4,372,188,393 | 29485862 |
| D2010027084 | 38574695 | 5,715,991,067 | 38541425 |
| D2010027085 | 35384099 | 5,239,883,060 | 35349685 |
| D2010027086 | 34569604 | 5,116,924,006 | 34537967 |
| D2010027087 | 38126710 | 5,645,610,852 | 38095981 |
| D2010027088 | 43926283 | 6,501,833,352 | 43889768 |
| D2010027089 | 40652531 | 6,014,499,434 | 40610674 |
| D2010027090 | 47126028 | 6,975,237,032 | 47090964 |
| D2010027091 | 44149945 | 6,536,931,944 | 44105817 |
| D2010027092 | 45634110 | 6,758,377,034 | 45596288 |
| D2010027093 | 38693473 | 5,729,991,234 | 38639660 |
| D2010027094 | 26505799 | 3,927,441,436 | 26479233 |
| D2010027095 | 26920070 | 3,974,302,244 | 26904161 |
| D2010027096 | 26565933 | 3,935,595,191 | 26542894 |
| D2010027097 | 28876899 | 4,277,495,888 | 28850140 |
| D2010027098 | 41142599 | 6,089,717,518 | 41101589 |
| D2010027099 | 40449972 | 5,993,694,819 | 40387987 |
| D2010027100 | 4040231 | 5,933,564,345 | 40002318 |
| D2010027101 | 37989681 | 5,623,325,276 | 37952054 |
| D2010027102 | 41286026 | 6,111,434,690 | 41243187 |
| D2010027103 | 4437659 | 6,514,488,427 | 43986687 |
| D2010027104 | 47764820 | 7,068,945,777 | 47724178 |
| D2010027105 | 44360705 | 6,565,083,813 | 44325745 |
| D2010027106 | 36822230 | 5,441,492,199 | 36785157 |
| D2010027107 | 35635142 | 5,268,962,871 | 35608647 |
| D2010027108 | 41481344 | 6,146,451,843 | 41445627 |
| D2010027109 | 31227060 | 4,616,712,317 | 31199708 |
| D2010027110 | 28116194 | 4,158,552,383 | 28090854 |
| D2010027111 | 40889424 | 6,059,901,249 | 40849589 |
| D2010027112 | 44716504 | 6,618,017,974 | 44679789 |
| D2010027113 | 41514795 | 6,140,172,084 | 41481408 |
| D2010027114 | 4929290 | 7,241,611,356 | 48988513 |
| D2010027115 | 43703268 | 6,457,823,467 | 43657337 |
| D2010027116 | 52662988 | 7,782,933,220 | 52603972 |
| D2010027117 | 42791136 | 6,316,197,290 | 42744430 |
| D2010027118 | 31746522 | 4,680,113,577 | 31715874 |
| D2010027119 | 41530493 | 6,132,950,459 | 41489755 |
| D2010027120 | 34229370 | 5,056,163,374 | 34200435 |
| D2010027121 | 42175590 | 6,232,983,677 | 42128709 |
| D2010027122 | 40941802 | 6,048,730,341 | 40915642 |
| D2010027123 | 40178694 | 5,936,360,101 | 40152397 |
| D2010027124 | 506823 | 7,400,346,618 | 49976840 |
| D2010027125 | 42513169 | 6,285,848,550 | 42485455 |
| D2010027126 | 37885273 | 5,587,729,673 | 37861613 |
| D2010027127 | 43219978 | 6,377,927,795 | 43194658 |
| D2010027128 | 51238174 | 7,570,924,009 | 51206293 |
| D2010027129 | 49235134 | 7,277,308,600 | 49203305 |
| D2010027130 | 40875120 | 6,050,791,621 | 40849850 |

| | | | |
|---|---|---|---|
| D2010027131 | 30183050 | 4,455,706,349 | 30162141 |
| D2010027132 | 4183772 | 6,081,678,717 | 41060119 |
| D2010027133 | 34797038 | 5,138,914,105 | 34774665 |
| D2010027134 | 34890399 | 5,152,922,081 | 34868405 |
| D2010027135 | 42510094 | 6,276,662,582 | 42482057 |
| D2010027136 | 46976851 | 6,955,468,495 | 46948391 |
| D2010027137 | 42572869 | 6,299,777,432 | 42546087 |
| D2010027138 | 56698630 | 8,391,692,113 | 56666930 |
| D2010027139 | 51100483 | 7,556,468,523 | 51067874 |
| D2010027140 | 49488553 | 7,320,537,299 | 49461210 |
| D2010027141 | 47299064 | 6,994,186,995 | 47267163 |
| D2010027142 | 39591140 | 5,864,505,989 | 39567973 |
| D2010027143 | 42221484 | 6,255,278,329 | 42193529 |
| D2010027144 | 43346651 | 6,422,372,509 | 43319207 |
| D2010027145 | 45296763 | 6,709,302,830 | 45270916 |
| D2010027146 | 4191333 | 6,082,650,830 | 41065466 |
| D2010027147 | 43305059 | 6,418,810,167 | 43278285 |
| D2010027148 | 37991527 | 5,620,741,328 | 37967921 |
| D2010027149 | 33943754 | 5,023,626,357 | 33922039 |
| D2010027150 | 42496416 | 6,288,583,228 | 42469957 |
| D2010027151 | 47571912 | 7,049,658,379 | 47543216 |
| D2010027152 | 4714053 | 6,967,637,044 | 46982766 |
| D2010027153 | 50745155 | 7,522,954,872 | 50713768 |
| D2010027154 | 39323674 | 5,814,596,507 | 39299540 |
| D2010027155 | 4048918 | 5,916,412,665 | 40020784 |
| D2010027156 | 45695009 | 6,764,282,435 | 45667356 |
| D2010027157 | 24158738 | 3,568,398,027 | 24144615 |
| D2010027158 | 22392958 | 3,299,850,251 | 22378214 |
| D2010027159 | 35184709 | 5,203,219,133 | 35161334 |
| D2010027160 | 33192644 | 4,907,816,279 | 33171010 |
| D2010027161 | 35504414 | 5,248,851,325 | 35480832 |
| D2010027162 | 48116839 | 7,123,629,874 | 48087515 |
| D2010027163 | 47407120 | 7,014,231,583 | 47377481 |
| D2010027164 | 47430464 | 7,020,635,877 | 47401774 |
| D2010027165 | 3751217 | 5,473,734,746 | 37027486 |
| D2010027166 | 32892190 | 4,866,631,629 | 32871797 |
| D2010027167 | 35856129 | 5,299,089,130 | 35833583 |
| D2010027168 | 36533508 | 5,401,933,432 | 36511491 |
| D2010027169 | 39974146 | 5,908,665,125 | 39946892 |
| D2010027170 | 41882345 | 6,160,984,394 | 41859792 |
| D2010027171 | 41187324 | 6,089,919,147 | 41135316 |
| D2010027172 | 41511035 | 6,140,134,676 | 41471345 |
| D2010027173 | 38401341 | 5,680,803,452 | 38359568 |
| D2010027174 | 39828605 | 5,882,802,157 | 39787526 |
| D2010027175 | 47482777 | 7,009,042,629 | 47438050 |
| D2010027176 | 59211852 | 8,754,391,095 | 59158823 |
| D2010027177 | 5410759 | 7,981,116,520 | 53948505 |
| D2010027178 | 48789953 | 7,217,202,914 | 48749346 |
| D2010027179 | 45350859 | 6,703,954,200 | 45300386 |
| D2010027180 | 48377930 | 7,154,248,578 | 48332374 |
| D2010027181 | 34308563 | 5,068,393,822 | 34257146 |
| D2010027182 | 42273937 | 6,241,150,779 | 42225456 |

| | | | |
|---|---|---|---|
| D2010027183 | 40951856 | 6,029,949,676 | 40927662 |
| D2010027184 | 49456273 | 7,300,856,145 | 49404003 |
| D2010027185 | 49446694 | 7,316,877,753 | 49391679 |
| D2010027226 | 87416258 | 12,749,632,406 | 87364513 |
| D2010027227 | 9053660 | 13,162,545,928 | 90001292 |
| D2010027228 | 98714438 | 14,427,583,456 | 98658311 |
| D2010027230 | 94682382 | 13,849,620,758 | 94622198 |
| D2010027231 | 87521936 | 12,803,363,237 | 87471961 |
| D2010027232 | 101151616 | 14,798,353,082 | 101094911 |
| D2010027233 | 99622918 | 14,550,238,968 | 99565444 |
| D2010027234 | 104227155 | 15,235,269,399 | 104167059 |
| D2010027235 | 94259734 | 13,716,507,317 | 94205375 |
| D2010027236 | 84607357 | 12,352,067,504 | 84559404 |
| D2010027237 | 94886803 | 13,871,674,986 | 94830771 |
| D2010027238 | 92124647 | 13,465,335,243 | 92068926 |
| D2010027239 | 85751574 | 12,539,094,405 | 85699048 |
| D2010027240 | 88929277 | 13,017,524,754 | 88877101 |
| D2010027241 | 89239141 | 13,044,728,157 | 89187820 |
| D2010027242 | 94542730 | 13,810,380,134 | 94490125 |
| D2010027243 | 88860513 | 12,959,113,845 | 88808742 |
| D2010027244 | 8174419 | 11,852,841,153 | 81027382 |
| D2010027245 | 78703798 | 11,505,824,643 | 78657981 |
| D2010027246 | 72593044 | 10,611,882,497 | 72550899 |
| D2010027247 | 74421633 | 10,763,745,057 | 74387231 |
| D2010027248 | 96333792 | 13,987,100,131 | 96288035 |
| D2010027249 | 95175065 | 13,762,388,685 | 95133673 |
| D2010027250 | 88696793 | 12,818,501,935 | 88657226 |
| D2010027251 | 8265496 | 11,961,147,653 | 82018304 |
| D2010027252 | 87955421 | 12,847,522,297 | 87903032 |
| D2010027253 | 91558836 | 13,368,866,734 | 91506820 |
| D2010027254 | 84812771 | 12,387,553,852 | 84762674 |
| D2010027255 | 91719526 | 13,278,089,932 | 91677033 |
| D2010027256 | 95986714 | 13,931,318,820 | 95944661 |
| D2010027257 | 83979719 | 12,161,598,389 | 83936243 |
| D2010027258 | 96786866 | 14,046,250,438 | 96743421 |
| D2010027259 | 98393902 | 14,363,925,762 | 98340217 |
| D2010027260 | 97951496 | 14,303,506,453 | 97894096 |
| D2010027261 | 89843165 | 13,113,872,882 | 89786365 |
| D2010027262 | 75553281 | 11,039,789,909 | 75511510 |
| D2010027263 | 85984052 | 12,419,833,847 | 85948418 |
| D2010027264 | 96758878 | 14,063,810,764 | 96712948 |
| D2010027265 | 90998763 | 13,193,949,692 | 90957884 |
| D2010027266 | 146727336 | 21,499,082,815 | 146678099 |
| D2010027267 | 812873 | 11,821,172,333 | 80955982 |
| D2010027268 | 75361588 | 11,011,626,649 | 75316872 |
| D2010027269 | 81440084 | 11,908,178,937 | 81391399 |
| D2010027270 | 72697636 | 10,622,234,532 | 72653603 |
| D2010027271 | 86550289 | 12,556,796,429 | 86510602 |
| D2010027272 | 86999967 | 12,609,039,041 | 86961667 |
| D2010027273 | 84143697 | 12,188,280,463 | 84104249 |
| D2010027274 | 93868420 | 13,594,827,292 | 93827300 |
| D2010027275 | 76458443 | 10,970,416,580 | 76425144 |

| | | | |
|---|---|---|---|
| D2010027276 | 72795988 | 10,496,259,459 | 72763022 |
| D2010027277 | 72974452 | 10,526,107,786 | 72938711 |
| D2010027278 | 66564773 | 9,579,272,551 | 66534040 |
| D2010027279 | 7296014 | 10,395,415,330 | 72062907 |
| D2010027280 | 89316752 | 12,871,457,619 | 89272613 |
| D2010027281 | 88462813 | 12,734,688,249 | 88422619 |
| D2010027282 | 79557099 | 11,415,287,582 | 79521566 |
| D2010027283 | 77587288 | 11,172,719,302 | 77550714 |
| D2010027284 | 79313114 | 11,450,912,795 | 79280686 |
| D2010027285 | 86525370 | 12,510,101,478 | 86485046 |
| D2010027286 | 80769415 | 11,662,613,940 | 80731504 |
| D2010027287 | 71114284 | 10,227,284,359 | 71080682 |
| D2010027288 | 767625 | 10,935,083,103 | 75972775 |
| D2010027289 | 78610524 | 11,344,299,163 | 78575213 |
| D2010027290 | 82688232 | 11,937,876,561 | 82648751 |
| D2010027291 | 74815151 | 10,731,950,873 | 74783041 |
| D2010027292 | 75193065 | 10,830,513,974 | 75158589 |
| D2010027293 | 76752597 | 11,072,614,815 | 76716861 |
| D2010027294 | 58667786 | 8,380,895,660 | 58639133 |
| D2010027295 | 85748072 | 12,348,378,154 | 85707911 |
| D2010027296 | 9181613 | 13,145,540,724 | 91041731 |
| D2010027297 | 9164513 | 13,092,474,655 | 91022388 |
| D2010027298 | 89815667 | 12,933,486,752 | 89774740 |
| D2010027299 | 85403071 | 12,274,920,927 | 85363133 |
| D2010027300 | 94910978 | 13,679,507,344 | 94867596 |
| D2010027301 | 94573790 | 13,594,270,869 | 94529082 |
| D2010027302 | 80928978 | 11,623,352,186 | 80891683 |
| D2010027303 | 92932142 | 13,339,285,842 | 92886217 |
| D2010027304 | 11019514 | 15,847,065,192 | 109972519 |
| D2010027305 | 121913543 | 17,580,773,240 | 121855313 |
| D2010027306 | 10784106 | 15,399,446,475 | 107035795 |
| D2010027307 | 100974822 | 14,568,357,635 | 100929010 |
| D2010027308 | 108157083 | 15,636,029,055 | 108107992 |
| D2010027309 | 95398843 | 13,713,907,194 | 95352670 |
| D2010027310 | 95361004 | 13,728,901,395 | 95318345 |
| D2010027311 | 83248503 | 12,010,289,131 | 83208609 |
| D2010027312 | 106718656 | 15,494,402,150 | 106668428 |
| D2010027313 | 72128633 | 10,425,873,822 | 72091346 |
| D2010027314 | 91157632 | 13,211,463,548 | 91111156 |
| D2010027315 | 91437929 | 13,131,494,936 | 91394978 |
| D2010027316 | 9145567 | 13,080,986,480 | 91004394 |
| D2010027317 | 10864894 | 15,583,348,853 | 108016245 |
| D2010027318 | 98614598 | 14,199,784,460 | 98565481 |
| D2010027319 | 86978389 | 12,465,492,814 | 86936813 |
| D2010027320 | 101875073 | 14,631,627,014 | 101828553 |
| D2010027321 | 92230408 | 13,230,002,340 | 92184312 |
| D2010027322 | 90920619 | 13,032,204,820 | 90876445 |
| D2010027323 | 102109326 | 14,641,006,137 | 102059422 |
| D2010027324 | 79791614 | 11,412,077,269 | 79754229 |
| D2010027325 | 103810196 | 14,915,020,105 | 103762380 |
| D2010027326 | 87220795 | 12,517,740,987 | 87178931 |
| D2010027327 | 86639993 | 12,464,824,464 | 86598901 |

| | | | |
|---|---|---|---|
| D2010027328 | 104747008 | 15,097,952,909 | 104697018 |
| D2010027329 | 97103115 | 13,976,251,838 | 97059074 |
| D2010027330 | 105507262 | 15,206,770,671 | 105458603 |
| D2010027331 | 92969157 | 13,304,731,562 | 92927171 |
| D2010027332 | 104137543 | 14,970,949,129 | 104090113 |
| D2010027333 | 98257306 | 14,095,891,473 | 98209052 |
| D2010027334 | 93678167 | 13,460,980,780 | 93635275 |
| D2010027335 | 91792575 | 13,223,248,802 | 91748565 |
| D2010027336 | 124178004 | 17,966,236,585 | 124116586 |
| D2010027337 | 101599695 | 14,624,008,332 | 101550876 |
| D2010027338 | 101977521 | 14,704,209,768 | 101931003 |
| D2010027339 | 88487599 | 12,704,559,657 | 88445512 |
| D2010027340 | 1041809 | 15,014,825,775 | 103951693 |
| D2010027341 | 89802202 | 12,905,189,339 | 89758916 |
| D2010027342 | 87461008 | 12,550,373,699 | 87419015 |
| D2010027343 | 83875465 | 12,047,125,945 | 83834209 |
| D2010027344 | 106851289 | 15,414,960,161 | 106801514 |
| D2010027345 | 100542507 | 14,472,309,807 | 100493969 |
| D2010027346 | 105883977 | 15,257,272,946 | 105833190 |
| D2010027347 | 108775642 | 15,684,121,997 | 108724715 |
| D2010027348 | 105649968 | 15,215,913,217 | 105599705 |
| D2010027349 | 113808820 | 16,395,034,411 | 113753815 |
| D2010027350 | 70391433 | 10,100,359,673 | 70358117 |
| D2010027351 | 70303101 | 10,017,309,256 | 70270317 |
| D2010027352 | 108372303 | 15,666,834,425 | 108322998 |
| D2010027353 | 94412724 | 13,589,597,067 | 94367022 |
| D2010027354 | 107796945 | 15,528,218,859 | 107747502 |
| D2010027356 | 96729870 | 13,910,248,107 | 96684033 |
| D2010027357 | 100214329 | 14,431,271,191 | 100168624 |
| D2010027358 | 100172233 | 14,441,978,466 | 100127933 |
| D2010027359 | 8260328 | 11,769,210,062 | 82021125 |
| D2010027360 | 85337586 | 12,249,866,114 | 85298901 |
| D2010027361 | 93543326 | 13,469,718,262 | 93499776 |
| D2010027362 | 92144902 | 13,239,094,761 | 92103994 |
| D2010027363 | 99326827 | 14,300,628,865 | 99283130 |
| D2010027364 | 92970971 | 13,385,482,979 | 92929913 |
| D2010027365 | 84495011 | 12,209,077,182 | 84456991 |
| D2010027366 | 8630152 | 12,402,108,097 | 85993341 |
| D2010027368 | 75940338 | 10,908,968,586 | 75905464 |
| D2010027369 | 82695073 | 11,937,502,892 | 82655621 |
| D2010027370 | 102844442 | 14,862,031,251 | 102799987 |
| D2010027371 | 91747941 | 13,250,970,591 | 91706510 |
| D2010027372 | 92349384 | 13,285,224,150 | 92309971 |
| D2010027373 | 82538248 | 11,895,959,694 | 82501952 |
| D2010027374 | 89814867 | 12,910,195,208 | 89772417 |
| D2010027375 | 86673247 | 12,512,975,908 | 86631453 |
| D2010027376 | 76580232 | 11,026,536,592 | 76542021 |
| D2010027377 | 79978589 | 11,482,274,220 | 79940430 |
| D2010027378 | 85709175 | 12,302,109,751 | 85671493 |
| D2010027379 | 95224363 | 13,718,172,700 | 95180902 |
| D2010027380 | 95563245 | 13,772,349,539 | 95521077 |
| D2010027381 | 8793476 | 12,511,822,847 | 87056433 |

| | | | |
|---|---|---|---|
| D2010027382 | 86513963 | 12,463,884,956 | 86474572 |
| D2010027383 | 88892403 | 12,817,029,404 | 88851808 |
| D2010027384 | 77200901 | 11,065,214,281 | 77165650 |
| D2010027385 | 85388413 | 12,291,314,787 | 85348221 |
| D2010027386 | 89129537 | 12,853,570,302 | 89089341 |
| D2010027387 | 84773337 | 12,165,110,158 | 84734353 |
| D2010027388 | 87649464 | 12,634,698,762 | 87610311 |
| D2010027389 | 86479361 | 12,421,534,111 | 86438182 |
| D2010027390 | 89289159 | 12,844,162,102 | 89245426 |
| D2010027391 | 92338180 | 13,283,454,078 | 92294795 |
| D2010027392 | 75585556 | 10,853,095,884 | 75550214 |
| D2010027393 | 80272819 | 11,572,458,301 | 80234948 |
| D2010027394 | 72330935 | 10,385,647,131 | 72297431 |
| D2010027395 | 8968145 | 12,838,751,320 | 89026188 |
| D2010027396 | 87176275 | 12,559,746,595 | 87139356 |
| D2010027397 | 83687251 | 11,979,870,212 | 83649906 |
| D2010027398 | 98366353 | 14,247,318,501 | 98321849 |
| D2010027399 | 95466494 | 13,790,278,824 | 95425141 |
| D2010027400 | 94705998 | 13,661,860,996 | 94664386 |
| D2010027401 | 91949397 | 13,248,219,151 | 91909156 |
| D2010027402 | 99598380 | 14,414,898,786 | 99551092 |
| D2010027403 | 77555693 | 11,205,873,756 | 77517492 |
| D2010027404 | 98665499 | 14,289,064,704 | 98619022 |
| D2010027405 | 8132747 | 11,624,608,428 | 80994762 |
| D2010027406 | 94881339 | 13,698,732,358 | 94840201 |
| D2010027407 | 95933420 | 13,891,949,018 | 95888867 |
| D2010027408 | 100282183 | 14,495,714,851 | 100235755 |
| D2010027409 | 84390557 | 12,132,981,048 | 84349293 |
| D2010027410 | 92342687 | 13,300,410,753 | 92300722 |
| D2010027411 | 95167582 | 13,682,218,940 | 95122841 |
| D2010027412 | 98536577 | 14,209,337,554 | 98491753 |
| D2010027413 | 105321000 | 15,253,254,353 | 105275376 |
| D2010027414 | 82369210 | 11,839,888,229 | 82334038 |
| D2010027415 | 105353751 | 15,269,004,635 | 105310068 |
| D2010027416 | 104302322 | 15,114,393,353 | 104257851 |
| D2010027417 | 95750142 | 13,827,644,883 | 95708455 |
| D2010027418 | 95465781 | 13,823,775,904 | 95423360 |
| D2010027419 | 97809309 | 14,126,873,937 | 97766391 |
| D2010027420 | 104676996 | 15,153,647,683 | 104634559 |
| D2010027421 | 89142584 | 12,759,191,400 | 89105270 |
| D2010027422 | 90357682 | 13,033,433,575 | 90317226 |
| D2010027423 | 86338777 | 12,372,612,752 | 86302828 |
| D2010027424 | 79432857 | 11,441,959,420 | 79398111 |
| D2010027425 | 90037768 | 12987226453 | 89994726 |
| D2010027426 | 110852029 | 16074823338 | 110802767 |
| D2010027427 | 101340848 | 14617309313 | 101295003 |
| D2010027428 | 97154369 | 14025221910 | 97110454 |
| D2010027429 | 87244163 | 12537636066 | 87206893 |
| D2010027430 | 102416124 | 14792399568 | 102369427 |
| D2010027431 | 100879873 | 14523301244 | 100835276 |
| D2010027432 | 89715713 | 12900376459 | 89675680 |
| D2010027433 | 95173968 | 13708954497 | 95127655 |

| | | | |
|---|---|---|---|
| D2010027434 | 91117850 | 13152944866 | 91073918 |
| D2010027435 | 92738568 | 13387280497 | 92695832 |
| D2010027436 | 105201648 | 15174732303 | 105151867 |
| D2010027437 | 102879799 | 14861025390 | 102833741 |
| D2010027438 | 104632641 | 15117666542 | 104584458 |
| D2010027439 | 106375826 | 15377562347 | 106325931 |
| D2010027440 | 73126894 | 10550174318 | 73095095 |
| D2010027441 | 76405386 | 10950120702 | 76372676 |
| D2010027442 | 102493739 | 14838126687 | 102449880 |
| D2010027443 | 85584560 | 12302746210 | 85543807 |
| D2010027444 | 104083447 | 15014238217 | 104037887 |
| D2010027445 | 100451790 | 14477003155 | 100406875 |
| D2010027446 | 87492074 | 12600094979 | 87451261 |
| D2010027447 | 87455603 | 12594964221 | 87415976 |
| D2010027448 | 82134730 | 11805481771 | 82098395 |
| D2010027449 | 122050591 | 17520249715 | 121992493 |
| D2010027450 | 129301150 | 18569358957 | 129242857 |
| D2010027451 | 130495383 | 18687960670 | 130432222 |
| D2010027452 | 130742330 | 18775127408 | 130682038 |
| D2010027453 | 83630055 | 12213200635 | 83587785 |
| D2010027454 | 77753780 | 11365123555 | 77697925 |
| D2010027455 | 73359321 | 10719413998 | 73313099 |
| D2010027456 | 72088703 | 10521951458 | 72041070 |
| D2010027457 | 89845143 | 13029261319 | 89801891 |
| D2010027458 | 96180874 | 13947838285 | 96133671 |
| D2010027487 | 65958484 | 9645994502 | 65914836 |
| D2010027890 | 116428468 | 16990360090 | 116377593 |
| D2010027891 | 120734520 | 17639698107 | 120689038 |
| D2010027892 | 105634121 | 15375390235 | 105586633 |
| D2010027893 | 180281116 | 26514362249 | 180185544 |
| D2010027894 | 195516466 | 28734856070 | 195415970 |
| D2010027895 | 205606127 | 30264879717 | 205503782 |
| D2010027896 | 200282024 | 29468670129 | 200178689 |
| D2010027898 | 114383405 | 16735901192 | 114341810 |
| D2010027899 | 97577285 | 14234178314 | 97535590 |
| D2010027900 | 120211314 | 17608459555 | 120165547 |
| D2010027901 | 83910464 | 12270642357 | 83877991 |
| D2010027902 | 100210138 | 14542833482 | 100162110 |
| D2010027903 | 115487852 | 16814409387 | 115435750 |
| D2010027904 | 106802698 | 15514447792 | 106749920 |
| D2010027905 | 122712584 | 17836119809 | 122653794 |
| D2010027906 | 123061623 | 18016621996 | 123013346 |
| D2010027907 | 100489698 | 14657516011 | 100448182 |
| D2010027908 | 113214399 | 16493150797 | 113169491 |
| D2010027909 | 90084985 | 13133968845 | 90046974 |
| D2010027910 | 60106385 | 8904952877 | 60073957 |
| D2010027911 | 71799452 | 10634951924 | 71763707 |
| D2010027912 | 48046343 | 7100565161 | 48020857 |
| D2010027913 | 52176509 | 7701958855 | 52143956 |
| D2010027914 | 205042119 | 29814366101 | 204945970 |
| D2010027915 | 175855121 | 25506618808 | 175773880 |
| D2010027459 | 90187455 | 13,036,951,195 | 90144165 |

| | | | |
|---|---|---|---|
| D2010027460 | 91377254 | 13,208,394,769 | 91336938 |
| D2010027461 | 86086221 | 12,566,556,301 | 86035796 |
| D2010027462 | 83587675 | 12,212,896,619 | 83534022 |
| D2010027463 | 81451065 | 11,914,793,646 | 81399489 |
| D2010027464 | 77598374 | 11,350,761,670 | 77539236 |
| D2010027465 | 71828123 | 10,492,802,604 | 71777268 |
| D2010027466 | 73795579 | 10,780,062,151 | 73758455 |
| D2010027467 | 79183427 | 11,574,151,047 | 79133707 |
| D2010027468 | 84116081 | 12,306,691,350 | 84062215 |
| D2010027469 | 93230509 | 13,463,083,236 | 93185587 |
| D2010027470 | 95096037 | 13,789,525,167 | 95047186 |
| D2010027471 | 101166987 | 14,693,218,876 | 101119043 |
| D2010027472 | 84009866 | 12,134,705,706 | 83970458 |
| D2010027473 | 88807243 | 12,884,583,026 | 88766348 |
| D2010027474 | 90562443 | 13,148,864,533 | 90518360 |
| D2010027475 | 97740534 | 14,150,858,457 | 97696612 |
| D2010027476 | 99463488 | 14,422,622,051 | 99419953 |
| D2010027478 | 54216752 | 7,812,239,146 | 54189819 |
| D2010027479 | 53533398 | 7,701,283,164 | 53504175 |
| D2010027480 | 44321277 | 6,347,952,449 | 44296650 |
| D2010027481 | 63023312 | 9,215,436,614 | 62980049 |
| D2010027482 | 67135522 | 9,784,543,808 | 67088846 |
| D2010027483 | 80906031 | 11,811,987,923 | 80855075 |
| D2010027484 | 73943942 | 10,754,528,740 | 73901359 |
| D2010027485 | 59467637 | 8,679,472,890 | 59429121 |
| D2010027486 | 72256537 | 10,588,704,586 | 72211259 |
| D2010027861 | 130835310 | 18,988,100,508 | 130770342 |
| D2010027862 | 144044304 | 21,000,891,574 | 143980537 |
| D2010027863 | 124983087 | 18,116,779,357 | 124925375 |
| D2010027864 | 115530024 | 16,731,768,239 | 115477588 |
| D2010027865 | 96252415 | 13,928,954,363 | 96216097 |
| D2010027866 | 105158689 | 15,244,238,066 | 105116119 |
| D2010027867 | 108875309 | 15,767,114,154 | 108830637 |
| D2010027868 | 84168539 | 12,126,532,018 | 84131322 |
| D2010027869 | 94236204 | 13,672,956,183 | 94194337 |
| D2010027870 | 105694582 | 15,336,267,293 | 105648494 |
| D2010027871 | 102353529 | 14,861,375,772 | 102305171 |
| D2010027872 | 104833363 | 15,233,943,351 | 104782111 |
| D2010027873 | 107229333 | 15,447,467,797 | 107182387 |
| D2010027874 | 97666420 | 14,048,011,280 | 97624457 |
| D2010027875 | 100103674 | 14,398,639,927 | 100059634 |
| D2010027876 | 75095097 | 10,795,322,395 | 75062157 |
| D2010027877 | 164907619 | 24,211,526,807 | 164818211 |
| D2010027878 | 215782481 | 31,765,524,192 | 215668198 |
| D2010027879 | 174137609 | 25,556,551,172 | 174038663 |
| D2010027880 | 208144917 | 30,582,535,219 | 208040065 |
| D2010027881 | 105194016 | 15,182,152,876 | 105147407 |
| D2010027882 | 86486718 | 12,417,833,494 | 86448162 |
| D2010027883 | 90267179 | 12,987,481,427 | 90224831 |
| D2010027884 | 71096010 | 10,178,470,339 | 71060966 |
| D2010027885 | 167521886 | 24,593,620,156 | 167430601 |
| D2010027886 | 214327865 | 31,514,168,117 | 214215896 |

| | | | |
|---|---|---|---|
| D2010027887 | 194377850 | 28,535,585,880 | 194281057 |
| D2010027888 | 199506081 | 29,303,801,780 | 199398952 |
| D2010027889 | 107552868 | 15,682,222,120 | 107510466 |

| median insert size | mean mapping quality | GC percentage | map rate | error rate |
|---|---|---|---|---|
| 306 | 34.785 | 41.25% | 99.77% | 0.98% |
| 326 | 34.656 | 41.40% | 99.77% | 1.01% |
| 321 | 34.663 | 41.31% | 99.79% | 1.00% |
| 325 | 34.741 | 41.34% | 99.77% | 1.01% |
| 335 | 34.912 | 41.39% | 99.77% | 1.02% |
| 331 | 34.676 | 41.16% | 99.79% | 1.01% |
| 332 | 34.955 | 41.37% | 99.78% | 1.02% |
| 325 | 34.629 | 41.48% | 99.77% | 1.02% |
| 346 | 34.683 | 41.31% | 99.78% | 1.04% |
| 358 | 34.903 | 41.09% | 99.78% | 1.04% |
| 339 | 34.795 | 41.26% | 99.79% | 1.01% |
| 353 | 34.613 | 41.28% | 99.78% | 1.02% |
| 346 | 34.785 | 41.35% | 99.76% | 1.03% |
| 328 | 34.840 | 40.97% | 99.79% | 1.02% |
| 350 | 34.855 | 41.31% | 99.78% | 1.03% |
| 330 | 34.684 | 41.29% | 99.75% | 1.01% |
| 330 | 34.871 | 41.28% | 99.78% | 1.03% |
| 342 | 34.773 | 41.24% | 99.77% | 1.02% |
| 340 | 34.894 | 41.28% | 99.79% | 1.01% |
| 338 | 34.646 | 41.37% | 99.73% | 1.04% |
| 343 | 34.787 | 41.34% | 99.75% | 1.02% |
| 333 | 34.664 | 41.47% | 99.76% | 1.01% |
| 332 | 34.668 | 41.33% | 99.79% | 1.02% |
| 331 | 34.857 | 41.32% | 99.79% | 1.01% |
| 333 | 34.807 | 41.34% | 99.77% | 1.02% |
| 344 | 34.777 | 41.46% | 99.76% | 1.04% |
| 338 | 34.631 | 41.36% | 99.78% | 1.02% |
| 346 | 34.935 | 41.23% | 99.78% | 1.04% |
| 347 | 35.012 | 41.56% | 99.73% | 1.01% |
| 344 | 34.829 | 41.35% | 99.77% | 1.03% |
| 344 | 34.791 | 41.36% | 99.74% | 1.01% |
| 331 | 34.793 | 41.21% | 99.80% | 1.01% |
| 343 | 34.898 | 41.40% | 99.73% | 1.04% |
| 354 | 34.927 | 41.35% | 99.75% | 1.03% |
| 350 | 34.704 | 41.27% | 99.78% | 1.04% |
| 345 | 34.674 | 41.55% | 99.75% | 1.04% |
| 341 | 34.787 | 41.04% | 99.78% | 1.03% |
| 347 | 34.788 | 41.22% | 99.78% | 1.02% |
| 350 | 34.783 | 41.38% | 99.78% | 1.03% |
| 343 | 34.501 | 41.44% | 99.73% | 1.01% |
| 343 | 34.584 | 41.11% | 99.80% | 1.34% |
| 348 | 34.545 | 41.29% | 99.79% | 1.08% |
| 342 | 34.670 | 41.02% | 99.79% | 1.07% |
| 338 | 34.567 | 41.26% | 99.80% | 1.06% |
| 353 | 34.495 | 41.48% | 99.78% | 1.09% |
| 346 | 34.555 | 41.54% | 99.76% | 1.07% |
| 340 | 34.588 | 41.46% | 99.78% | 1.10% |
| 331 | 34.480 | 41.57% | 99.80% | 1.10% |
| 337 | 34.667 | 41.41% | 99.82% | 1.09% |
| 353 | 34.862 | 41.11% | 99.78% | 1.10% |
| 340 | 34.649 | 41.38% | 99.78% | 1.12% |

| | | | | |
|---|---|---|---|---|
| 336 | 34.532 | 41.43% | 99.80% | 1.11% |
| 336 | 34.725 | 41.40% | 99.80% | 1.11% |
| 322 | 34.836 | 41.65% | 99.77% | 1.10% |
| 341 | 34.898 | 41.62% | 99.79% | 1.12% |
| 334 | 34.509 | 41.51% | 99.81% | 1.13% |
| 332 | 34.735 | 41.27% | 99.80% | 1.11% |
| 348 | 34.733 | 41.13% | 99.80% | 1.11% |
| 350 | 34.671 | 41.33% | 99.81% | 0.98% |
| 337 | 34.647 | 41.48% | 99.83% | 0.98% |
| 334 | 34.622 | 41.31% | 99.82% | 0.96% |
| 339 | 34.652 | 41.54% | 99.80% | 0.97% |
| 351 | 34.873 | 41.06% | 99.80% | 1.06% |
| 337 | 34.680 | 41.35% | 99.80% | 1.02% |
| 330 | 34.712 | 41.34% | 99.80% | 1.04% |
| 352 | 34.592 | 41.25% | 99.79% | 1.05% |
| 346 | 34.722 | 41.41% | 99.77% | 1.04% |
| 336 | 34.597 | 41.46% | 99.76% | 1.01% |
| 344 | 34.794 | 41.28% | 99.78% | 1.04% |
| 342 | 34.641 | 41.41% | 99.79% | 1.04% |
| 352 | 34.699 | 41.21% | 99.80% | 1.05% |
| 346 | 34.843 | 41.29% | 99.78% | 1.02% |
| 342 | 34.827 | 41.35% | 99.78% | 1.03% |
| 356 | 34.372 | 41.06% | 99.78% | 1.05% |
| 356 | 34.648 | 41.21% | 99.81% | 1.04% |
| 339 | 34.693 | 41.48% | 99.79% | 1.03% |
| 342 | 34.712 | 41.37% | 99.79% | 1.02% |
| 335 | 34.602 | 41.51% | 99.77% | 1.01% |
| 342 | 34.644 | 41.38% | 99.80% | 1.11% |
| 334 | 34.726 | 41.36% | 99.81% | 1.10% |
| 327 | 34.784 | 41.64% | 99.78% | 0.94% |
| 313 | 34.712 | 41.80% | 99.78% | 0.92% |
| 319 | 34.729 | 41.10% | 99.82% | 0.83% |
| 338 | 34.568 | 41.49% | 99.80% | 0.84% |
| 347 | 34.710 | 41.43% | 99.80% | 0.85% |
| 341 | 34.646 | 41.41% | 99.79% | 0.84% |
| 339 | 34.362 | 41.59% | 99.76% | 0.84% |
| 316 | 34.763 | 41.69% | 99.80% | 0.83% |
| 319 | 34.576 | 41.75% | 99.79% | 0.84% |
| 317 | 34.744 | 41.93% | 99.76% | 0.85% |
| 309 | 34.731 | 41.63% | 99.78% | 0.82% |
| 332 | 34.762 | 41.51% | 99.77% | 0.83% |
| 340 | 34.698 | 41.69% | 99.77% | 0.84% |
| 336 | 34.552 | 41.58% | 99.77% | 0.85% |
| 317 | 34.919 | 41.44% | 99.79% | 0.86% |
| 297 | 34.905 | 41.82% | 99.78% | 0.83% |
| 326 | 34.759 | 41.53% | 99.75% | 0.84% |
| 308 | 34.534 | 41.74% | 99.77% | 0.83% |
| 308 | 34.751 | 41.79% | 99.79% | 0.83% |
| 318 | 34.750 | 41.73% | 99.79% | 0.84% |
| 328 | 34.512 | 41.60% | 99.80% | 0.84% |
| 321 | 34.748 | 41.47% | 99.77% | 0.84% |
| 324 | 34.741 | 41.27% | 99.84% | 1.23% |

| | | | | |
|---|---|---|---|---|
| 327 | 34.739 | 41.24% | 99.84% | 1.27% |
| 309 | 34.685 | 41.16% | 99.84% | 1.21% |
| 305 | 34.678 | 41.54% | 99.83% | 1.20% |
| 291 | 34.615 | 41.06% | 99.86% | 1.21% |
| 308 | 34.425 | 41.37% | 99.84% | 1.27% |
| 316 | 34.622 | 41.32% | 99.82% | 1.30% |
| 309 | 34.497 | 41.37% | 99.83% | 1.29% |
| 312 | 34.772 | 41.52% | 99.83% | 1.28% |
| 301 | 34.739 | 41.32% | 99.83% | 1.27% |
| 297 | 34.668 | 41.65% | 99.83% | 1.26% |
| 292 | 34.634 | 41.59% | 99.83% | 1.29% |
| 300 | 34.629 | 41.46% | 99.83% | 1.28% |
| 307 | 34.902 | 41.46% | 99.84% | 1.29% |
| 316 | 34.628 | 41.54% | 99.83% | 1.30% |
| 322 | 34.621 | 41.39% | 99.83% | 1.30% |
| 309 | 34.453 | 41.34% | 99.84% | 1.29% |
| 307 | 34.486 | 41.19% | 99.83% | 1.33% |
| 329 | 34.638 | 41.10% | 99.85% | 1.30% |
| 316 | 34.568 | 41.40% | 99.83% | 1.28% |
| 295 | 34.913 | 41.30% | 99.73% | 1.07% |
| 312 | 34.502 | 41.12% | 99.70% | 1.10% |
| 306 | 34.882 | 41.15% | 99.72% | 1.06% |
| 306 | 34.581 | 41.06% | 99.68% | 1.13% |
| 326 | 34.605 | 41.15% | 99.71% | 1.13% |
| 311 | 34.508 | 41.39% | 99.69% | 1.11% |
| 321 | 34.687 | 41.44% | 99.75% | 1.15% |
| 310 | 34.579 | 41.57% | 99.75% | 1.12% |
| 289 | 34.711 | 41.49% | 99.73% | 1.10% |
| 294 | 34.792 | 41.50% | 99.76% | 1.10% |
| 295 | 34.590 | 41.48% | 99.76% | 1.09% |
| 296 | 34.466 | 41.29% | 99.76% | 1.11% |
| 277 | 34.759 | 41.79% | 99.70% | 1.04% |
| 294 | 34.401 | 41.68% | 99.72% | 1.11% |
| 289 | 34.654 | 41.77% | 99.66% | 1.06% |
| 281 | 34.663 | 41.29% | 99.77% | 1.07% |
| 297 | 34.579 | 41.56% | 99.84% | 1.27% |
| 290 | 34.743 | 41.65% | 99.83% | 1.22% |
| 295 | 34.485 | 41.60% | 99.83% | 1.27% |
| 293 | 34.460 | 41.55% | 99.82% | 1.26% |
| 284 | 34.595 | 41.10% | 99.82% | 1.11% |
| 277 | 34.554 | 41.61% | 99.81% | 1.10% |
| 287 | 34.584 | 41.87% | 99.81% | 1.11% |
| 287 | 34.621 | 41.92% | 99.81% | 1.10% |
| 307 | 34.782 | 41.81% | 99.82% | 1.08% |
| 312 | 34.621 | 41.38% | 99.83% | 1.08% |
| 311 | 34.527 | 41.57% | 99.81% | 1.07% |
| 322 | 34.574 | 41.70% | 99.80% | 1.07% |
| 321 | 34.478 | 41.27% | 99.82% | 1.17% |
| 308 | 34.639 | 41.38% | 99.81% | 1.16% |
| 314 | 34.590 | 41.56% | 99.81% | 1.16% |
| 307 | 34.704 | 41.83% | 99.77% | 1.15% |
| 297 | 34.674 | 41.84% | 99.81% | 1.05% |

| | | | | |
|---|---|---|---|---|
| 304 | 34.653 | 41.65% | 99.75% | 1.10% |
| 309 | 34.672 | 41.47% | 99.77% | 1.05% |
| 315 | 35.095 | 41.22% | 99.78% | 1.06% |
| 310 | 34.633 | 41.48% | 99.78% | 1.12% |
| 309 | 34.522 | 41.56% | 99.77% | 1.14% |
| 306 | 34.553 | 41.60% | 99.80% | 1.12% |
| 307 | 34.432 | 41.67% | 99.82% | 1.12% |
| 291 | 34.443 | 41.51% | 99.84% | 1.07% |
| 304 | 34.481 | 41.45% | 99.82% | 1.06% |
| 300 | 34.704 | 41.55% | 99.83% | 1.08% |
| 314 | 34.581 | 41.35% | 99.83% | 1.07% |
| 337 | 34.509 | 41.44% | 99.82% | 0.97% |
| 341 | 34.776 | 41.46% | 99.81% | 0.96% |
| 358 | 34.525 | 41.29% | 99.83% | 0.98% |
| 341 | 34.798 | 41.14% | 99.82% | 0.97% |
| 343 | 34.680 | 41.15% | 99.83% | 0.97% |
| 337 | 34.961 | 41.30% | 99.82% | 0.96% |
| 333 | 34.654 | 41.48% | 99.80% | 0.95% |
| 342 | 34.630 | 41.44% | 99.81% | 0.96% |
| 291 | 34.464 | 41.60% | 99.74% | 0.82% |
| 302 | 34.802 | 41.81% | 99.69% | 0.83% |
| 307 | 34.797 | 41.65% | 99.72% | 0.82% |
| 309 | 34.616 | 41.55% | 99.74% | 0.82% |
| 285 | 34.893 | 41.24% | 99.73% | 1.10% |
| 295 | 35.017 | 41.08% | 99.77% | 1.05% |
| 283 | 34.614 | 41.51% | 99.73% | 1.07% |
| 302 | 34.790 | 41.33% | 99.77% | 1.10% |
| 304 | 34.808 | 41.52% | 99.74% | 1.08% |
| 301 | 34.800 | 41.45% | 99.73% | 1.08% |
| 313 | 34.647 | 41.34% | 99.75% | 1.09% |
| 317 | 34.791 | 41.30% | 99.68% | 1.12% |
| 305 | 34.827 | 42.34% | 99.61% | 0.85% |
| 311 | 34.661 | 41.74% | 99.62% | 0.84% |
| 312 | 34.656 | 41.58% | 99.59% | 0.84% |
| 321 | 34.713 | 41.77% | 99.69% | 0.84% |
| 296 | 34.684 | 41.61% | 99.80% | 0.93% |
| 311 | 34.481 | 41.68% | 99.80% | 0.96% |
| 312 | 34.805 | 41.56% | 99.82% | 0.95% |
| 314 | 34.649 | 41.65% | 99.80% | 0.97% |
| 298 | 34.809 | 41.90% | 99.71% | 0.83% |
| 301 | 34.587 | 41.83% | 99.72% | 0.83% |
| 309 | 34.528 | 41.80% | 99.71% | 0.84% |
| 306 | 34.836 | 41.22% | 99.74% | 0.84% |
| 302 | 34.643 | 41.58% | 99.82% | 1.07% |
| 309 | 34.693 | 41.89% | 99.80% | 1.09% |
| 315 | 34.540 | 41.61% | 99.82% | 1.08% |
| 319 | 34.742 | 41.31% | 99.83% | 1.08% |
| 306 | 34.568 | 41.47% | 99.80% | 1.07% |
| 302 | 34.735 | 41.24% | 99.84% | 1.08% |
| 317 | 34.842 | 41.42% | 99.83% | 1.07% |
| 322 | 34.581 | 41.61% | 99.83% | 1.09% |
| 287 | 34.827 | 41.53% | 99.83% | 1.24% |

| | | | | |
|---|---|---|---|---|
| 290 | 34.633 | 41.48% | 99.84% | 1.24% |
| 300 | 34.610 | 41.55% | 99.84% | 1.23% |
| 298 | 34.532 | 41.19% | 99.84% | 1.28% |
| 298 | 34.829 | 41.47% | 99.83% | 1.23% |
| 288 | 34.747 | 41.67% | 99.80% | 1.23% |
| 300 | 34.598 | 41.60% | 99.82% | 1.27% |
| 291 | 34.686 | 41.59% | 99.83% | 1.23% |
| 291 | 34.560 | 41.55% | 99.72% | 1.25% |
| 313 | 34.543 | 41.46% | 99.80% | 1.29% |
| 313 | 34.501 | 41.37% | 99.82% | 1.28% |
| 308 | 34.620 | 41.33% | 99.77% | 1.29% |
| 309 | 34.398 | 41.87% | 99.68% | 1.07% |
| 303 | 34.498 | 41.47% | 99.60% | 1.07% |
| 324 | 34.554 | 41.36% | 99.60% | 1.06% |
| 309 | 34.422 | 41.03% | 99.66% | 1.07% |
| 310 | 34.828 | 41.88% | 99.72% | 0.84% |
| 327 | 34.664 | 41.69% | 99.65% | 0.83% |
| 332 | 34.694 | 41.49% | 99.68% | 0.84% |
| 327 | 34.625 | 41.56% | 99.65% | 0.84% |
| 297 | 34.816 | 41.18% | 99.84% | 1.18% |
| 302 | 34.530 | 41.47% | 99.79% | 1.18% |
| 315 | 34.525 | 41.63% | 99.83% | 1.25% |
| 302 | 34.589 | 41.67% | 99.81% | 1.18% |
| 309 | 34.558 | 41.64% | 99.83% | 1.31% |
| 319 | 34.780 | 41.35% | 99.83% | 1.33% |
| 339 | 34.558 | 41.31% | 99.83% | 1.34% |
| 315 | 34.637 | 41.43% | 99.80% | 1.29% |
| 329 | 34.862 | 41.86% | 99.76% | 0.85% |
| 327 | 34.789 | 41.76% | 99.79% | 0.85% |
| 335 | 34.597 | 41.84% | 99.78% | 0.85% |
| 332 | 34.510 | 41.84% | 99.67% | 0.86% |
| 299 | 34.613 | 41.63% | 99.82% | 1.26% |
| 320 | 34.519 | 41.42% | 99.83% | 1.38% |
| 340 | 34.466 | 41.21% | 99.83% | 1.34% |
| 338 | 34.689 | 41.14% | 99.84% | 1.34% |
| 336 | 34.831 | 41.60% | 99.76% | 0.85% |
| 322 | 34.696 | 41.79% | 99.76% | 0.85% |
| 332 | 34.518 | 41.78% | 99.76% | 0.85% |
| 329 | 34.967 | 41.76% | 99.77% | 0.85% |
| 309 | 34.542 | 41.27% | 99.83% | 1.34% |
| 310 | 34.710 | 41.32% | 99.82% | 1.28% |
| 321 | 34.784 | 41.20% | 99.83% | 1.35% |
| 320 | 34.661 | 41.35% | 99.82% | 1.32% |
| 309 | 34.905 | 41.82% | 99.77% | 0.93% |
| 314 | 34.783 | 41.77% | 99.75% | 0.91% |
| 332 | 34.685 | 41.24% | 99.76% | 1.08% |
| 310 | 34.722 | 41.52% | 99.78% | 1.14% |
| 346 | 34.724 | 41.14% | 99.81% | 1.11% |
| 344 | 34.681 | 41.13% | 99.80% | 1.12% |
| 307 | 34.701 | 41.56% | 99.81% | 1.11% |
| 339 | 34.621 | 41.47% | 99.78% | 1.08% |
| 343 | 34.634 | 41.06% | 99.80% | 1.09% |

| | | | | |
|---|---|---|---|---|
| 312 | 34.636 | 41.36% | 99.80% | 1.14% |
| 340 | 34.466 | 41.28% | 99.80% | 1.09% |
| 340 | 34.686 | 41.29% | 99.80% | 1.10% |
| 341 | 34.649 | 41.12% | 99.81% | 1.11% |
| 304 | 34.541 | 41.36% | 99.84% | 1.31% |
| 337 | 34.673 | 41.43% | 99.80% | 1.10% |
| 352 | 34.632 | 41.11% | 99.80% | 1.12% |
| 303 | 34.677 | 41.65% | 99.81% | 1.23% |
| 359 | 34.769 | 41.18% | 99.80% | 1.11% |
| 335 | 34.660 | 41.32% | 99.80% | 1.09% |
| 340 | 34.555 | 41.43% | 99.81% | 1.09% |
| 312 | 34.467 | 41.27% | 99.84% | 1.28% |
| 333 | 34.878 | 41.35% | 99.80% | 1.09% |
| 343 | 34.824 | 41.13% | 99.83% | 1.11% |
| 339 | 34.859 | 41.29% | 99.83% | 1.11% |
| 318 | 34.783 | 41.41% | 99.81% | 1.30% |
| 341 | 34.703 | 41.21% | 99.83% | 1.11% |
| 295 | 34.804 | 41.68% | 99.81% | 1.11% |
| 359 | 34.716 | 40.99% | 99.81% | 1.15% |
| 357 | 34.563 | 41.31% | 99.82% | 1.35% |
| 335 | 34.407 | 41.36% | 99.84% | 1.35% |
| 303 | 34.550 | 41.58% | 99.81% | 1.15% |
| 345 | 34.540 | 41.46% | 99.83% | 1.31% |
| 326 | 34.478 | 41.02% | 99.84% | 1.30% |
| 306 | 34.621 | 41.41% | 99.82% | 1.13% |
| 350 | 34.602 | 41.51% | 99.83% | 1.21% |
| 309 | 34.649 | 41.39% | 99.82% | 1.14% |
| 351 | 34.589 | 41.35% | 99.82% | 1.19% |
| 345 | 34.744 | 41.11% | 99.82% | 1.21% |
| 297 | 34.580 | 41.50% | 99.81% | 1.10% |
| 351 | 34.727 | 41.22% | 99.82% | 1.21% |
| 360 | 34.756 | 41.03% | 99.70% | 0.85% |
| 303 | 34.597 | 41.46% | 99.82% | 1.12% |
| 345 | 34.472 | 41.57% | 99.70% | 0.85% |
| 354 | 34.664 | 41.48% | 99.62% | 0.85% |
| 307 | 34.447 | 41.55% | 99.81% | 1.13% |
| 346 | 34.641 | 41.69% | 99.65% | 0.85% |
| 352 | 34.648 | 42.03% | 99.76% | 0.88% |
| 345 | 34.501 | 41.95% | 99.78% | 0.86% |
| 306 | 34.720 | 41.59% | 99.79% | 1.13% |
| 347 | 34.531 | 41.90% | 99.80% | 0.85% |
| 361 | 34.864 | 41.74% | 99.78% | 0.85% |
| 289 | 34.766 | 41.34% | 99.84% | 1.03% |
| 353 | 34.826 | 41.12% | 99.84% | 1.38% |
| 338 | 34.515 | 41.34% | 99.83% | 1.35% |
| 306 | 34.790 | 41.47% | 99.83% | 1.06% |
| 335 | 34.620 | 41.48% | 99.83% | 1.33% |
| 330 | 34.813 | 41.56% | 99.82% | 1.33% |
| 299 | 34.789 | 41.41% | 99.84% | 1.05% |
| 277 | 34.893 | 41.95% | 99.67% | 0.82% |
| 311 | 34.818 | 41.45% | 99.83% | 1.08% |
| 295 | 34.871 | 42.02% | 99.62% | 0.82% |

| | | | | |
|---|---|---|---|---|
| 297 | 34.780 | 41.74% | 99.64% | 0.83% |
| 310 | 35.021 | 41.89% | 99.80% | 0.95% |
| 310 | 34.678 | 41.80% | 99.59% | 0.83% |
| 292 | 34.643 | 41.28% | 99.71% | 0.82% |
| 301 | 34.735 | 41.72% | 99.68% | 0.83% |
| 312 | 34.713 | 41.61% | 99.80% | 0.94% |
| 307 | 34.471 | 41.86% | 99.68% | 0.84% |
| 299 | 34.860 | 41.89% | 99.63% | 0.83% |
| 291 | 34.700 | 42.02% | 99.78% | 0.84% |
| 300 | 34.677 | 41.81% | 99.76% | 0.84% |
| 313 | 34.487 | 41.64% | 99.81% | 0.94% |
| 302 | 34.766 | 41.98% | 99.79% | 0.84% |
| 309 | 34.998 | 41.71% | 99.78% | 0.83% |
| 298 | 34.888 | 41.86% | 99.77% | 0.84% |
| 324 | 34.647 | 41.62% | 99.81% | 0.97% |
| 297 | 34.706 | 41.76% | 99.78% | 0.84% |
| 299 | 34.778 | 41.77% | 99.78% | 0.83% |
| 303 | 34.796 | 41.85% | 99.62% | 0.83% |
| 302 | 34.537 | 41.65% | 99.82% | 0.94% |
| 274 | 34.631 | 42.06% | 99.78% | 0.83% |
| 287 | 34.597 | 41.78% | 99.69% | 0.85% |
| 292 | 34.686 | 41.74% | 99.60% | 0.83% |
| 309 | 34.581 | 41.63% | 99.80% | 0.95% |
| 291 | 34.816 | 41.76% | 99.25% | 0.83% |
| 287 | 34.710 | 41.64% | 99.81% | 1.23% |
| 289 | 34.562 | 41.73% | 99.79% | 1.26% |
| 306 | 34.733 | 41.73% | 99.81% | 0.95% |
| 286 | 34.642 | 41.78% | 99.82% | 1.22% |
| 294 | 34.844 | 41.57% | 99.81% | 1.24% |
| 314 | 34.765 | 41.74% | 99.79% | 0.94% |
| 292 | 34.509 | 41.63% | 99.81% | 1.13% |
| 305 | 34.721 | 41.25% | 99.81% | 1.13% |
| 308 | 34.554 | 41.54% | 99.80% | 0.94% |
| 305 | 34.662 | 41.15% | 99.79% | 1.14% |
| 305 | 34.491 | 41.34% | 99.76% | 1.16% |
| 296 | 34.579 | 41.65% | 99.82% | 0.94% |
| 301 | 34.598 | 41.56% | 99.81% | 1.26% |
| 297 | 34.752 | 41.49% | 99.83% | 1.23% |
| 308 | 34.589 | 41.72% | 99.82% | 0.94% |
| 299 | 34.653 | 41.53% | 99.83% | 1.26% |
| 300 | 34.601 | 41.54% | 99.84% | 1.28% |
| 303 | 34.642 | 41.45% | 99.83% | 1.15% |
| 313 | 34.756 | 41.73% | 99.79% | 0.94% |
| 317 | 34.690 | 41.52% | 99.81% | 1.14% |
| 308 | 34.591 | 41.48% | 99.81% | 0.95% |
| 321 | 34.655 | 41.34% | 99.82% | 1.17% |
| 319 | 34.597 | 41.41% | 99.82% | 1.16% |
| 332 | 34.546 | 41.21% | 99.84% | 1.33% |
| 310 | 34.715 | 41.41% | 99.80% | 0.94% |
| 310 | 34.576 | 41.52% | 99.83% | 1.28% |
| 322 | 34.440 | 41.65% | 99.82% | 1.30% |
| 320 | 34.665 | 41.57% | 99.80% | 0.95% |

| | | | | |
|---|---|---|---|---|
| 319 | 34.736 | 41.90% | 99.81% | 1.29% |
| 297 | 34.608 | 41.55% | 99.80% | 1.11% |
| 330 | 34.755 | 41.19% | 99.82% | 0.97% |
| 311 | 34.571 | 41.36% | 99.80% | 0.94% |
| 309 | 34.560 | 41.71% | 99.80% | 0.95% |
| 308 | 34.812 | 41.50% | 99.82% | 0.95% |
| 321 | 34.741 | 41.55% | 99.81% | 0.96% |
| 304 | 34.628 | 41.88% | 99.63% | 0.93% |
| 316 | 34.450 | 41.86% | 99.71% | 0.95% |
| 316 | 34.848 | 41.87% | 99.67% | 0.94% |
| 321 | 34.512 | 41.61% | 99.69% | 0.94% |
| 330 | 34.596 | 41.41% | 99.78% | 1.08% |
| 318 | 34.690 | 41.57% | 99.76% | 1.10% |
| 312 | 34.699 | 41.44% | 99.76% | 1.05% |
| 303 | 34.720 | 41.28% | 99.82% | 1.05% |
| 297 | 34.632 | 41.92% | 99.77% | 0.94% |
| 313 | 34.496 | 41.65% | 99.75% | 0.93% |
| 304 | 34.679 | 41.89% | 99.73% | 0.94% |
| 321 | 34.569 | 41.95% | 99.69% | 0.96% |
| 311 | 34.657 | 41.22% | 99.81% | 0.94% |
| 303 | 34.732 | 41.62% | 99.81% | 0.93% |
| 311 | 34.797 | 41.52% | 99.80% | 0.94% |
| 321 | 34.737 | 41.72% | 99.78% | 0.94% |
| 297 | 34.720 | 41.86% | 99.81% | 0.94% |
| 316 | 34.840 | 41.56% | 99.81% | 0.96% |
| 322 | 34.675 | 41.51% | 99.83% | 0.94% |
| 337 | 34.767 | 41.44% | 99.81% | 0.96% |
| 325 | 34.846 | 41.54% | 99.77% | 0.97% |
| 322 | 34.575 | 41.53% | 99.82% | 0.96% |
| 313 | 34.785 | 41.57% | 99.82% | 0.94% |
| 330 | 34.674 | 41.39% | 99.82% | 0.96% |
| 303 | 34.763 | 41.59% | 99.71% | 1.08% |
| 323 | 34.502 | 41.44% | 99.75% | 1.18% |
| 339 | 34.744 | 41.27% | 99.77% | 1.15% |
| 338 | 34.635 | 41.08% | 99.72% | 1.14% |
| 321 | 34.633 | 41.30% | 99.67% | 1.12% |
| 320 | 34.579 | 41.49% | 99.70% | 1.12% |
| 318 | 34.434 | 41.25% | 99.78% | 1.10% |
| 329 | 34.508 | 41.46% | 99.73% | 1.13% |
| 279 | 34.625 | 41.90% | 99.82% | 0.95% |
| 294 | 34.701 | 41.70% | 99.82% | 0.93% |
| 293 | 34.777 | 41.32% | 99.84% | 0.93% |
| 306 | 34.707 | 41.63% | 99.83% | 0.96% |
| 314 | 34.804 | 41.65% | 99.81% | 0.95% |
| 301 | 34.814 | 41.50% | 99.82% | 0.91% |
| 297 | 34.556 | 41.87% | 99.78% | 0.94% |
| 312 | 34.673 | 41.55% | 99.82% | 0.96% |
| 354 | 34.554 | 41.64% | 99.76% | 0.84% |
| 361 | 34.629 | 41.47% | 99.75% | 0.84% |
| 372 | 34.820 | 41.58% | 99.77% | 0.84% |
| 374 | 34.600 | 41.56% | 99.72% | 0.85% |
| 389 | 34.708 | 41.33% | 99.65% | 0.84% |

| | | | | |
|---|---|---|---|---|
| 380 | 34.712 | 41.34% | 99.74% | 0.85% |
| 369 | 34.491 | 41.68% | 99.73% | 0.85% |
| 360 | 34.374 | 41.26% | 99.74% | 0.84% |
| 355 | 34.517 | 41.52% | 99.72% | 0.82% |
| 369 | 34.647 | 41.43% | 99.70% | 0.82% |
| 371 | 34.578 | 41.48% | 99.75% | 0.81% |
| 378 | 34.593 | 41.39% | 99.72% | 0.81% |
| 388 | 34.477 | 41.65% | 99.72% | 0.82% |
| 382 | 34.424 | 41.31% | 99.75% | 0.82% |
| 375 | 34.243 | 41.61% | 99.72% | 0.82% |
| 359 | 34.470 | 41.54% | 99.71% | 0.82% |
| 358 | 34.362 | 41.56% | 99.75% | 0.82% |
| 367 | 34.670 | 41.38% | 99.71% | 0.82% |
| 370 | 34.604 | 41.64% | 99.75% | 0.82% |
| 372 | 34.445 | 41.59% | 99.68% | 0.82% |
| 379 | 34.263 | 41.43% | 99.70% | 0.81% |
| 361 | 34.420 | 41.38% | 99.72% | 0.83% |
| 385 | 34.569 | 41.44% | 99.73% | 0.82% |
| 366 | 34.392 | 41.65% | 99.69% | 0.82% |
| 366 | 34.589 | 41.38% | 99.74% | 0.82% |
| 360 | 34.455 | 41.39% | 99.68% | 0.81% |
| 361 | 34.225 | 41.50% | 99.73% | 0.81% |
| 374 | 34.588 | 41.24% | 99.72% | 0.82% |
| 388 | 34.442 | 41.40% | 99.71% | 0.82% |
| 365 | 34.484 | 41.60% | 99.41% | 0.83% |
| 371 | 34.438 | 41.54% | 99.74% | 0.82% |
| 369 | 34.420 | 41.60% | 99.75% | 0.83% |
| 384 | 34.505 | 41.43% | 99.72% | 0.82% |
| 404 | 34.263 | 41.36% | 99.63% | 0.83% |
| 369 | 34.773 | 41.38% | 99.74% | 0.81% |
| 407 | 34.558 | 41.28% | 99.74% | 0.83% |
| 433 | 34.250 | 41.21% | 99.73% | 0.83% |
| 366 | 34.203 | 41.29% | 99.68% | 0.81% |
| 392 | 34.533 | 41.46% | 99.70% | 0.82% |
| 352 | 34.512 | 41.37% | 99.68% | 0.81% |
| 386 | 34.639 | 41.39% | 99.74% | 0.85% |
| 396 | 34.649 | 41.56% | 99.71% | 0.85% |
| 378 | 34.679 | 41.35% | 99.67% | 0.85% |
| 404 | 34.510 | 41.53% | 99.68% | 0.86% |
| 406 | 34.654 | 41.38% | 99.71% | 0.86% |
| 403 | 34.553 | 41.39% | 99.71% | 0.85% |
| 404 | 34.510 | 41.59% | 99.40% | 0.85% |
| 385 | 34.588 | 41.67% | 99.64% | 0.85% |
| 383 | 34.378 | 41.69% | 99.72% | 0.86% |
| 397 | 34.597 | 41.46% | 99.73% | 0.85% |
| 378 | 34.433 | 41.53% | 99.77% | 0.84% |
| 405 | 34.300 | 41.48% | 99.74% | 0.84% |
| 424 | 34.630 | 41.17% | 99.77% | 0.86% |
| 405 | 34.669 | 41.26% | 99.78% | 0.85% |
| 387 | 34.681 | 41.44% | 99.74% | 0.86% |
| 379 | 34.449 | 41.55% | 99.50% | 0.85% |
| 392 | 34.714 | 41.46% | 99.76% | 0.82% |

| | | | | |
|---|---|---|---|---|
| 403 | 34.255 | 41.46% | 99.32% | 0.85% |
| 381 | 34.667 | 41.40% | 99.76% | 0.82% |
| 425 | 34.695 | 41.33% | 99.75% | 0.84% |
| 423 | 34.459 | 41.46% | 99.72% | 0.84% |
| 396 | 34.594 | 41.66% | 99.74% | 0.84% |
| 385 | 34.735 | 41.56% | 99.74% | 0.82% |
| 379 | 34.312 | 41.75% | 99.73% | 0.83% |
| 353 | 34.671 | 41.58% | 99.75% | 0.83% |
| 358 | 34.726 | 41.70% | 99.75% | 0.83% |
| 364 | 34.481 | 41.48% | 99.76% | 0.82% |
| 369 | 34.623 | 41.60% | 99.05% | 0.83% |
| 375 | 34.491 | 41.75% | 99.75% | 0.82% |
| 372 | 34.427 | 41.69% | 99.73% | 0.82% |
| 366 | 34.346 | 41.63% | 99.72% | 0.82% |
| 354 | 34.567 | 41.59% | 99.75% | 0.82% |
| 353 | 34.577 | 41.67% | 99.73% | 0.83% |
| 373 | 34.455 | 41.37% | 99.72% | 0.82% |
| 375 | 34.452 | 41.54% | 99.75% | 0.83% |
| 390 | 34.652 | 41.45% | 99.75% | 0.83% |
| 392 | 34.536 | 41.37% | 99.77% | 0.83% |
| 383 | 34.431 | 41.17% | 99.76% | 0.82% |
| 368 | 34.343 | 41.57% | 98.00% | 0.82% |
| 358 | 34.649 | 41.62% | 99.77% | 0.82% |
| 404 | 34.457 | 41.28% | 99.76% | 0.83% |
| 411 | 34.317 | 41.49% | 99.41% | 0.83% |
| 402 | 34.659 | 41.51% | 99.77% | 0.82% |
| 434 | 34.529 | 41.17% | 99.79% | 0.84% |
| 443 | 34.632 | 41.35% | 99.73% | 0.85% |
| 413 | 34.726 | 41.37% | 99.74% | 0.82% |
| 416 | 34.384 | 41.35% | 99.73% | 0.83% |
| 411 | 34.386 | 41.31% | 99.75% | 0.83% |
| 378 | 34.741 | 41.37% | 99.77% | 0.82% |
| 378 | 34.437 | 41.48% | 99.77% | 0.83% |
| 373 | 34.601 | 41.63% | 99.74% | 0.83% |
| 406 | 34.806 | 41.50% | 99.73% | 0.84% |
| 413 | 34.524 | 41.46% | 99.74% | 0.83% |
| 401 | 34.301 | 41.50% | 99.74% | 0.84% |
| 404 | 34.560 | 41.48% | 99.72% | 0.83% |
| 378 | 34.291 | 41.69% | 99.21% | 0.83% |
| 349 | 34.156 | 41.25% | 99.74% | 0.84% |
| 367 | 34.781 | 41.63% | 99.67% | 0.85% |
| 366 | 34.794 | 41.55% | 99.72% | 0.84% |
| 381 | 34.623 | 41.47% | 99.69% | 0.84% |
| 403 | 34.393 | 41.57% | 99.70% | 0.86% |
| 386 | 34.613 | 41.54% | 99.70% | 0.86% |
| 363 | 34.531 | 41.70% | 99.71% | 0.86% |
| 358 | 34.391 | 41.57% | 99.67% | 0.85% |
| 356 | 34.702 | 41.48% | 99.72% | 0.84% |
| 366 | 34.516 | 41.63% | 99.62% | 0.85% |
| 356 | 34.668 | 41.89% | 99.69% | 0.85% |
| 404 | 34.453 | 41.55% | 99.61% | 0.85% |
| 403 | 34.220 | 41.30% | 99.65% | 0.86% |

| | | | | |
|---|---|---|---|---|
| 374 | 34.422 | 41.49% | 99.68% | 0.86% |
| 364 | 34.637 | 41.62% | 98.64% | 0.85% |
| 358 | 34.561 | 41.45% | 99.60% | 0.84% |
| 345 | 34.722 | 41.63% | 99.76% | 0.95% |
| 341 | 34.755 | 41.62% | 99.77% | 0.95% |
| 338 | 34.698 | 41.77% | 99.79% | 0.95% |
| 346 | 34.532 | 41.48% | 99.76% | 0.95% |
| 346 | 34.794 | 41.49% | 99.78% | 0.96% |
| 343 | 34.875 | 41.53% | 99.79% | 0.95% |
| 343 | 34.766 | 41.65% | 99.77% | 0.96% |
| 339 | 34.889 | 41.65% | 99.75% | 0.95% |
| 360 | 34.904 | 41.45% | 99.78% | 0.95% |
| 343 | 34.436 | 41.77% | 99.79% | 0.96% |
| 348 | 34.599 | 41.58% | 99.77% | 0.95% |
| 354 | 34.688 | 41.33% | 99.75% | 0.95% |
| 349 | 34.629 | 41.51% | 99.76% | 0.95% |
| 343 | 34.601 | 41.42% | 99.78% | 0.95% |
| 345 | 34.694 | 41.59% | 99.77% | 0.95% |
| 338 | 34.632 | 41.67% | 99.78% | 0.94% |
| 332 | 34.984 | 41.61% | 99.76% | 0.95% |
| 342 | 34.736 | 41.37% | 99.76% | 0.94% |
| 346 | 34.653 | 41.49% | 99.77% | 0.95% |
| 352 | 34.891 | 41.45% | 99.77% | 0.96% |
| 357 | 35.110 | 41.06% | 99.84% | 1.13% |
| 337 | 34.581 | 41.34% | 99.82% | 1.09% |
| 334 | 34.570 | 41.19% | 99.76% | 1.11% |
| 337 | 34.721 | 41.26% | 99.63% | 1.12% |
| 354 | 34.639 | 41.43% | 99.65% | 0.95% |
| 357 | 34.720 | 41.51% | 99.62% | 0.95% |
| 342 | 34.470 | 41.51% | 99.72% | 0.95% |
| 352 | 34.694 | 41.39% | 99.77% | 0.95% |
| 342 | 35.053 | 41.10% | 99.65% | 1.10% |
| 338 | 34.830 | 41.12% | 99.84% | 1.09% |
| 351 | 34.616 | 41.21% | 99.63% | 1.11% |
| 344 | 34.889 | 41.19% | 99.83% | 1.09% |
| 346 | 34.650 | 41.42% | 99.77% | 0.95% |
| 346 | 34.658 | 41.39% | 99.78% | 0.96% |
| 363 | 34.598 | 41.61% | 99.75% | 0.96% |
| 349 | 34.612 | 41.44% | 99.79% | 0.95% |
| 328 | 34.915 | 40.81% | 99.83% | 1.11% |
| 348 | 34.802 | 41.18% | 99.81% | 1.08% |
| 343 | 34.882 | 41.21% | 99.83% | 1.10% |
| 269 | 35.128 | 41.35% | 99.81% | 0.98% |
| 354 | 34.550 | 41.56% | 99.77% | 0.95% |
| 358 | 34.558 | 41.28% | 99.79% | 0.96% |
| 339 | 34.600 | 41.46% | 99.78% | 0.95% |
| 347 | 34.757 | 41.53% | 99.77% | 0.96% |
| 340 | 34.656 | 41.26% | 99.84% | 1.10% |
| 337 | 34.788 | 41.06% | 99.83% | 1.10% |
| 344 | 34.811 | 41.09% | 99.83% | 1.11% |
| 333 | 34.807 | 41.29% | 99.82% | 1.08% |
| 339 | 34.856 | 40.94% | 99.84% | 1.21% |

| | | | | |
|---|---|---|---|---|
| 347 | 35.004 | 40.85% | 99.84% | 1.25% |
| 338 | 34.603 | 41.08% | 99.83% | 1.24% |
| 348 | 34.749 | 40.78% | 99.84% | 1.27% |
| 340 | 34.795 | 41.09% | 99.84% | 1.24% |
| 330 | 34.896 | 41.14% | 99.82% | 1.25% |
| 336 | 34.605 | 41.28% | 99.83% | 1.26% |
| 328 | 34.815 | 41.24% | 99.83% | 1.29% |
| 336 | 34.895 | 41.14% | 99.82% | 1.26% |
| 331 | 34.687 | 40.90% | 99.84% | 1.24% |
| 327 | 34.663 | 41.05% | 99.82% | 1.23% |
| 334 | 34.772 | 41.04% | 99.81% | 1.25% |
| 343 | 34.729 | 41.05% | 99.84% | 1.30% |
| 327 | 34.521 | 41.10% | 99.83% | 1.29% |
| 336 | 34.660 | 41.34% | 99.83% | 1.25% |
| 328 | 34.955 | 41.38% | 99.82% | 1.24% |
| 341 | 34.633 | 41.18% | 99.84% | 1.28% |
| 340 | 34.660 | 41.16% | 99.83% | 1.27% |
| 339 | 34.875 | 41.19% | 99.83% | 1.24% |
| 353 | 34.598 | 40.90% | 99.83% | 1.34% |
| 360 | 34.752 | 41.13% | 99.84% | 1.28% |
| 343 | 34.856 | 40.95% | 99.85% | 1.24% |
| 337 | 34.883 | 41.30% | 99.83% | 1.26% |
| 342 | 34.733 | 41.22% | 99.84% | 1.25% |
| 348 | 34.800 | 41.09% | 99.84% | 1.27% |
| 342 | 34.623 | 41.12% | 99.84% | 1.26% |
| 348 | 34.593 | 41.23% | 99.83% | 1.26% |
| 352 | 34.694 | 40.95% | 99.84% | 1.29% |
| 360 | 34.786 | 41.04% | 99.84% | 1.31% |
| 337 | 35.048 | 41.07% | 99.85% | 1.26% |
| 357 | 34.741 | 41.27% | 99.83% | 1.24% |
| 340 | 34.845 | 40.84% | 99.84% | 1.24% |
| 336 | 34.936 | 40.97% | 99.85% | 1.24% |
| 334 | 34.757 | 41.06% | 99.83% | 1.22% |
| 338 | 34.620 | 41.16% | 99.83% | 1.28% |
| 341 | 34.701 | 41.18% | 99.84% | 1.27% |
| 342 | 34.811 | 41.06% | 99.82% | 1.13% |
| 335 | 34.601 | 41.31% | 99.83% | 1.09% |
| 350 | 34.596 | 41.45% | 99.81% | 1.14% |
| 342 | 34.648 | 41.39% | 99.81% | 1.10% |
| 342 | 34.732 | 41.36% | 99.83% | 1.28% |
| 344 | 35.012 | 41.04% | 99.85% | 1.27% |
| 343 | 34.667 | 41.20% | 99.84% | 1.24% |
| 357 | 34.807 | 41.13% | 99.83% | 1.27% |
| 357 | 34.641 | 41.14% | 99.84% | 1.33% |
| 339 | 34.792 | 41.09% | 99.84% | 1.29% |
| 351 | 34.737 | 41.17% | 99.83% | 1.30% |
| 352 | 34.957 | 41.15% | 99.83% | 1.31% |
| 356 | 34.859 | 41.03% | 99.83% | 1.29% |
| 336 | 34.483 | 41.07% | 99.83% | 1.33% |
| 346 | 34.594 | 40.90% | 99.84% | 1.28% |
| 357 | 34.686 | 40.97% | 99.84% | 1.31% |
| 358 | 34.764 | 41.08% | 99.84% | 1.29% |

| | | | | |
|---|---|---|---|---|
| 341 | 34.701 | 41.03% | 99.83% | 1.27% |
| 351 | 34.778 | 40.94% | 99.84% | 1.27% |
| 340 | 35.004 | 41.00% | 99.84% | 1.25% |
| 342 | 34.752 | 40.92% | 99.84% | 1.32% |
| 340 | 34.790 | 41.14% | 99.84% | 1.28% |
| 336 | 34.511 | 41.17% | 99.83% | 1.30% |
| 345 | 34.573 | 41.06% | 99.84% | 1.29% |
| 354 | 34.735 | 41.21% | 99.83% | 1.27% |
| 338 | 34.759 | 41.38% | 99.82% | 1.22% |
| 345 | 34.919 | 41.09% | 99.84% | 1.26% |
| 336 | 34.690 | 41.14% | 99.84% | 1.25% |
| 343 | 34.898 | 41.14% | 99.84% | 1.29% |
| 345 | 34.741 | 41.23% | 99.83% | 1.25% |
| 351 | 34.823 | 40.92% | 99.83% | 1.27% |
| 357 | 34.750 | 40.99% | 99.84% | 1.31% |
| 362 | 34.592 | 40.99% | 99.84% | 1.30% |
| 341 | 34.798 | 41.14% | 99.84% | 1.26% |
| 353 | 34.675 | 41.16% | 99.83% | 1.27% |
| 347 | 34.834 | 41.31% | 99.83% | 1.25% |
| 334 | 34.828 | 41.25% | 99.84% | 1.27% |
| 336 | 34.666 | 41.38% | 99.83% | 1.28% |
| 337 | 35.043 | 41.33% | 99.83% | 1.26% |
| 352 | 34.365 | 41.04% | 99.84% | 1.32% |
| 351 | 34.621 | 40.99% | 99.84% | 1.39% |
| 345 | 34.897 | 41.30% | 99.84% | 1.23% |
| 337 | 34.836 | 41.37% | 99.81% | 1.28% |
| 342 | 34.697 | 41.16% | 99.84% | 1.26% |
| 346 | 34.811 | 41.19% | 99.84% | 1.28% |
| 333 | 34.422 | 41.24% | 99.84% | 1.27% |
| 339 | 34.571 | 41.09% | 99.85% | 1.26% |
| 346 | 34.666 | 41.15% | 99.84% | 1.31% |
| 348 | 34.540 | 41.11% | 99.85% | 1.31% |
| 336 | 34.856 | 41.13% | 99.83% | 1.27% |
| 344 | 34.607 | 41.22% | 99.84% | 1.29% |
| 334 | 34.793 | 41.19% | 99.84% | 1.26% |
| 336 | 34.931 | 40.97% | 99.85% | 1.23% |
| 332 | 34.539 | 41.33% | 99.79% | 1.23% |
| 338 | 34.411 | 41.03% | 99.85% | 1.26% |
| 345 | 34.460 | 41.13% | 99.84% | 1.29% |
| 345 | 34.431 | 41.35% | 99.83% | 1.25% |
| 331 | 34.922 | 41.02% | 99.85% | 1.23% |
| 337 | 34.672 | 41.26% | 99.84% | 1.24% |
| 329 | 34.615 | 41.05% | 99.86% | 1.26% |
| 338 | 34.773 | 40.99% | 99.85% | 1.24% |
| 343 | 34.659 | 41.13% | 99.83% | 1.27% |
| 341 | 34.694 | 41.16% | 99.83% | 1.24% |
| 355 | 34.706 | 41.00% | 99.83% | 1.27% |
| 346 | 34.834 | 41.14% | 99.84% | 1.30% |
| 330 | 34.956 | 40.88% | 99.84% | 1.28% |
| 339 | 34.776 | 41.21% | 99.84% | 1.25% |
| 340 | 34.744 | 41.02% | 99.84% | 1.24% |
| 340 | 34.515 | 41.03% | 99.85% | 1.28% |

| | | | | |
|---|---|---|---|---|
| 342 | 34.764 | 41.09% | 99.84% | 1.26% |
| 341 | 34.696 | 41.02% | 99.84% | 1.25% |
| 339 | 34.638 | 40.96% | 99.84% | 1.30% |
| 346 | 34.576 | 41.13% | 99.83% | 1.27% |
| 336 | 34.715 | 41.21% | 99.83% | 1.26% |
| 341 | 34.920 | 41.03% | 99.84% | 1.29% |
| 333 | 34.777 | 41.06% | 99.84% | 1.23% |
| 343 | 34.721 | 41.15% | 99.84% | 1.26% |
| 346 | 35.048 | 41.04% | 99.83% | 1.28% |
| 347 | 34.836 | 41.02% | 99.84% | 1.28% |
| 349 | 34.843 | 40.75% | 99.84% | 1.29% |
| 352 | 34.566 | 40.97% | 99.84% | 1.27% |
| 347 | 34.868 | 40.85% | 99.84% | 1.31% |
| 346 | 34.640 | 41.24% | 99.83% | 1.25% |
| 325 | 34.900 | 40.89% | 99.84% | 1.23% |
| 350 | 34.850 | 41.25% | 99.84% | 1.29% |
| 329 | 34.703 | 41.03% | 99.84% | 1.21% |
| 325 | 34.826 | 41.04% | 99.84% | 1.24% |
| 326 | 34.762 | 40.99% | 99.84% | 1.24% |
| 326 | 35.067 | 40.80% | 99.84% | 1.23% |
| 335 | 34.808 | 40.95% | 99.84% | 1.22% |
| 331 | 34.790 | 41.32% | 99.82% | 1.23% |
| 330 | 34.670 | 41.26% | 99.83% | 1.20% |
| 345 | 34.408 | 41.35% | 99.83% | 1.29% |
| 332 | 34.854 | 41.15% | 99.84% | 1.24% |
| 332 | 34.955 | 40.93% | 99.83% | 1.21% |
| 336 | 34.983 | 41.17% | 99.83% | 1.22% |
| 347 | 34.748 | 41.02% | 99.83% | 1.29% |
| 337 | 34.733 | 40.91% | 99.83% | 1.28% |
| 339 | 34.628 | 41.17% | 99.83% | 1.28% |
| 331 | 34.751 | 41.34% | 99.82% | 1.25% |
| 324 | 34.915 | 41.31% | 99.84% | 1.20% |
| 316 | 34.854 | 41.35% | 99.84% | 1.29% |
| 318 | 34.681 | 41.00% | 99.85% | 1.21% |
| 317 | 34.932 | 41.01% | 99.85% | 1.20% |
| 334 | 34.801 | 41.08% | 99.84% | 1.26% |
| 318 | 34.888 | 41.18% | 99.83% | 1.22% |
| 327 | 34.784 | 41.33% | 99.84% | 1.25% |
| 316 | 34.738 | 41.37% | 99.84% | 1.20% |
| 330 | 34.686 | 41.03% | 99.85% | 1.32% |
| 335 | 34.716 | 41.20% | 99.83% | 1.26% |
| 329 | 34.761 | 40.87% | 99.85% | 1.30% |
| 336 | 34.667 | 40.88% | 99.85% | 1.25% |
| 339 | 34.788 | 41.24% | 99.84% | 1.25% |
| 329 | 34.674 | 41.35% | 99.84% | 1.21% |
| 331 | 34.730 | 41.23% | 99.84% | 1.25% |
| 325 | 34.707 | 41.30% | 99.83% | 1.24% |
| 337 | 34.678 | 40.85% | 99.85% | 1.27% |
| 341 | 34.500 | 41.09% | 99.84% | 1.24% |
| 339 | 34.838 | 40.87% | 99.84% | 1.27% |
| 345 | 34.973 | 40.85% | 99.85% | 1.29% |
| 344 | 34.609 | 40.95% | 99.84% | 1.27% |

| | | | | |
|---|---|---|---|---|
| 344 | 34.726 | 40.93% | 99.84% | 1.24% |
| 344 | 34.692 | 41.09% | 99.84% | 1.23% |
| 338 | 34.585 | 41.35% | 99.83% | 1.23% |
| 328 | 34.800 | 41.18% | 99.84% | 1.22% |
| 323 | 34.630 | 41.27% | 99.83% | 1.23% |
| 329 | 34.809 | 41.39% | 99.82% | 1.23% |
| 334 | 34.618 | 40.99% | 99.84% | 1.25% |
| 330 | 34.617 | 41.16% | 99.83% | 1.31% |
| 337 | 34.702 | 41.24% | 99.85% | 1.22% |
| 341 | 34.823 | 41.14% | 99.83% | 1.28% |
| 328 | 34.753 | 41.26% | 99.84% | 1.23% |
| 333 | 34.794 | 41.34% | 99.84% | 1.25% |
| 337 | 34.901 | 41.17% | 99.84% | 1.27% |
| 344 | 34.619 | 40.98% | 99.84% | 1.27% |
| 338 | 34.745 | 41.10% | 99.84% | 1.29% |
| 344 | 34.519 | 40.94% | 99.83% | 1.32% |
| 332 | 34.736 | 41.19% | 99.83% | 1.32% |
| 337 | 34.526 | 41.29% | 99.82% | 1.33% |
| 334 | 34.474 | 41.40% | 99.83% | 1.32% |
| 319 | 34.530 | 41.33% | 99.79% | 1.06% |
| 327 | 34.410 | 41.55% | 99.77% | 1.09% |
| 334 | 34.672 | 41.27% | 99.80% | 1.10% |
| 333 | 34.377 | 41.31% | 99.79% | 1.10% |
| 337 | 34.698 | 41.40% | 99.83% | 1.10% |
| 342 | 34.800 | 41.13% | 99.81% | 1.12% |
| 332 | 34.426 | 41.40% | 99.79% | 1.07% |
| 311 | 34.579 | 41.57% | 99.82% | 1.07% |
| 292 | 34.792 | 41.39% | 99.83% | 1.04% |
| 314 | 34.840 | 41.38% | 99.82% | 1.08% |
| 319 | 34.639 | 41.49% | 99.79% | 0.92% |
| 310 | 34.946 | 41.69% | 99.79% | 0.92% |
| 294 | 34.695 | 41.91% | 99.76% | 0.90% |
| 309 | 34.928 | 41.91% | 99.78% | 0.91% |
| 281 | 34.968 | 41.71% | 99.84% | 1.02% |
| 302 | 34.696 | 41.71% | 99.81% | 1.07% |
| 283 | 34.836 | 41.64% | 99.83% | 1.02% |
| 294 | 34.814 | 41.45% | 99.83% | 1.04% |
| 303 | 34.608 | 41.41% | 99.80% | 1.13% |
| 299 | 34.521 | 41.71% | 99.82% | 1.09% |
| 297 | 34.608 | 41.42% | 99.80% | 1.12% |
| 297 | 34.751 | 41.52% | 99.81% | 1.11% |
| 280 | 34.646 | 41.71% | 99.83% | 1.03% |
| 298 | 34.659 | 41.66% | 99.82% | 1.06% |
| 286 | 34.716 | 41.81% | 99.81% | 1.04% |
| 305 | 34.722 | 41.36% | 99.82% | 1.06% |
| 306 | 34.930 | 41.71% | 99.78% | 0.83% |
| 280 | 34.710 | 41.58% | 99.81% | 0.83% |
| 349 | 34.768 | 41.50% | 99.81% | 0.85% |
| 337 | 34.384 | 41.81% | 99.77% | 0.86% |
| 306 | 34.766 | 41.51% | 99.81% | 1.11% |
| 294 | 34.655 | 41.18% | 99.82% | 1.13% |
| 347 | 34.683 | 41.42% | 99.82% | 1.12% |

| | | | | |
|---|---|---|---|---|
| 324 | 34.839 | 41.27% | 99.83% | 1.12% |
| 328 | 34.542 | 41.58% | 99.81% | 1.10% |
| 322 | 34.562 | 41.38% | 99.79% | 1.08% |
| 330 | 34.629 | 41.24% | 99.79% | 1.08% |
| 333 | 34.527 | 41.51% | 99.78% | 1.08% |
| 336 | 34.635 | 41.57% | 99.78% | 1.10% |
| 329 | 34.603 | 41.19% | 99.81% | 1.09% |
| 329 | 34.645 | 41.57% | 99.80% | 1.09% |
| 325 | 34.656 | 41.56% | 99.78% | 1.07% |
| 333 | 34.685 | 41.18% | 99.82% | 1.11% |
| 332 | 34.786 | 41.44% | 99.82% | 1.11% |
| 334 | 34.706 | 41.39% | 99.82% | 1.09% |
| 339 | 35.035 | 41.08% | 99.81% | 1.13% |
| 343 | 34.746 | 41.13% | 99.84% | 1.10% |
| 329 | 34.620 | 41.54% | 99.81% | 1.11% |
| 329 | 34.955 | 41.41% | 99.83% | 1.10% |
| 327 | 34.836 | 41.49% | 99.84% | 1.09% |
| 339 | 34.448 | 41.29% | 99.83% | 1.33% |
| 346 | 34.654 | 41.03% | 99.80% | 1.34% |
| 354 | 34.381 | 41.04% | 99.79% | 1.38% |
| 357 | 34.439 | 41.39% | 99.79% | 1.10% |
| 341 | 34.623 | 41.65% | 99.77% | 1.11% |
| 344 | 34.643 | 41.48% | 99.75% | 1.09% |
| 323 | 34.792 | 40.99% | 99.81% | 1.06% |
| 349 | 34.650 | 41.52% | 99.79% | 1.09% |
| 337 | 34.514 | 41.40% | 99.80% | 1.05% |
| 311 | 34.560 | 41.58% | 99.81% | 1.14% |
| 295 | 34.652 | 41.63% | 99.81% | 1.08% |
| 307 | 34.597 | 41.51% | 99.82% | 1.14% |
| 308 | 34.700 | 41.56% | 99.81% | 1.15% |
| 290 | 34.683 | 41.37% | 99.84% | 1.27% |
| 304 | 34.806 | 41.56% | 99.83% | 1.25% |
| 293 | 34.640 | 41.43% | 99.83% | 1.25% |
| 313 | 34.551 | 41.40% | 99.82% | 1.31% |
| 312 | 34.509 | 41.10% | 99.84% | 1.13% |
| 305 | 34.317 | 41.43% | 99.82% | 1.13% |
| 312 | 34.562 | 41.42% | 99.81% | 1.13% |
| 302 | 34.695 | 41.83% | 99.79% | 1.11% |
| 321 | 34.638 | 41.53% | 99.83% | 1.31% |
| 324 | 34.557 | 41.53% | 99.83% | 1.32% |
| 334 | 34.436 | 41.41% | 99.83% | 1.33% |
| 329 | 34.646 | 41.35% | 99.83% | 1.33% |
| 322 | 34.698 | 41.63% | 99.76% | 0.92% |
| 301 | 34.810 | 41.98% | 99.76% | 0.90% |
| 326 | 34.698 | 41.77% | 99.75% | 0.92% |
| 315 | 34.843 | 41.79% | 99.78% | 0.91% |
| 312 | 34.665 | 41.67% | 99.82% | 1.29% |
| 326 | 34.483 | 41.55% | 99.82% | 1.34% |
| 323 | 34.518 | 41.52% | 99.82% | 1.32% |
| 331 | 34.503 | 41.34% | 99.81% | 1.36% |
| 339 | 34.730 | 41.63% | 99.76% | 0.93% |
| 298 | 34.642 | 41.75% | 99.77% | 0.91% |

| | | | | |
|---|---|---|---|---|
| 326 | 34.577 | 41.63% | 99.78% | 0.92% |
| 320 | 34.779 | 41.77% | 99.76% | 0.92% |
| 298 | 34.719 | 41.39% | 99.83% | 1.07% |

| depth | coverage(1X) | coverage(5X) | coverage(10X) | coverage(15X) |
|---|---|---|---|---|
| 15.3214X | 96.61% | 93.44% | 82.54% | 56.00% |
| 14.4143X | 96.47% | 92.91% | 80.43% | 50.21% |
| 15.601X | 96.55% | 93.47% | 83.46% | 58.58% |
| 14.8535X | 96.44% | 93.07% | 81.58% | 53.49% |
| 14.5102X | 96.48% | 92.98% | 80.80% | 51.01% |
| 15.6018X | 96.46% | 93.31% | 83.39% | 59.10% |
| 16.5998X | 96.56% | 93.84% | 85.60% | 65.03% |
| 14.7315X | 96.56% | 93.21% | 81.38% | 52.27% |
| 14.9458X | 96.48% | 93.17% | 81.88% | 54.03% |
| 13.781X | 96.40% | 92.42% | 78.14% | 45.53% |
| 14.9423X | 96.48% | 93.15% | 81.94% | 54.39% |
| 13.4116X | 96.45% | 92.34% | 76.84% | 42.04% |
| 13.9756X | 96.46% | 92.66% | 79.01% | 46.69% |
| 14.9636X | 96.49% | 93.03% | 81.31% | 53.86% |
| 14.6368X | 96.53% | 93.11% | 81.17% | 51.93% |
| 16.1493X | 96.63% | 93.79% | 84.69% | 61.93% |
| 15.9654X | 96.57% | 93.63% | 84.25% | 60.89% |
| 14.1045X | 96.46% | 92.65% | 79.27% | 47.90% |
| 14.4267X | 96.49% | 92.93% | 80.45% | 50.32% |
| 13.5301X | 96.43% | 92.41% | 77.24% | 42.78% |
| 14.9388X | 96.48% | 93.20% | 82.03% | 54.26% |
| 14.4953X | 96.49% | 93.07% | 80.84% | 50.62% |
| 15.9203X | 96.53% | 93.56% | 84.20% | 60.94% |
| 15.9068X | 96.56% | 93.63% | 84.27% | 60.89% |
| 14.3386X | 96.49% | 92.79% | 80.04% | 49.62% |
| 14.6925X | 96.53% | 93.11% | 81.29% | 52.22% |
| 15.8239X | 96.58% | 93.66% | 84.16% | 60.18% |
| 13.266X | 96.36% | 92.07% | 76.05% | 40.91% |
| 13.0122X | 96.46% | 92.24% | 75.30% | 38.13% |
| 13.7636X | 96.41% | 92.58% | 78.44% | 45.13% |
| 15.5548X | 96.53% | 93.52% | 83.63% | 58.73% |
| 14.4949X | 96.54% | 92.91% | 80.15% | 50.21% |
| 15.5135X | 96.54% | 93.43% | 83.34% | 58.16% |
| 14.2089X | 96.51% | 92.88% | 79.87% | 48.63% |
| 14.4497X | 96.40% | 92.83% | 80.52% | 50.79% |
| 14.0973X | 96.53% | 92.91% | 79.59% | 47.48% |
| 13.6972X | 96.49% | 92.41% | 77.33% | 44.16% |
| 15.499X | 96.54% | 93.43% | 83.31% | 58.26% |
| 12.2842X | 96.33% | 91.39% | 71.55% | 32.41% |
| 15.2081X | 96.59% | 93.41% | 82.79% | 56.22% |
| 6.6566X | 95.07% | 72.37% | 19.31% | 1.69% |
| 8.3951X | 95.74% | 82.97% | 37.47% | 5.97% |
| 8.3185X | 95.81% | 82.20% | 36.43% | 6.07% |
| 10.6154X | 96.12% | 88.96% | 59.25% | 18.98% |
| 7.1361X | 95.50% | 76.34% | 23.45% | 2.35% |
| 9.0107X | 96.03% | 85.44% | 43.68% | 8.38% |
| 10.0892X | 96.05% | 88.16% | 54.91% | 14.83% |
| 11.132X | 96.18% | 89.89% | 63.28% | 22.29% |
| 11.9649X | 96.26% | 90.93% | 69.25% | 29.62% |
| 10.8247X | 96.09% | 89.19% | 61.30% | 20.80% |
| 10.3895X | 96.04% | 88.53% | 57.49% | 17.02% |

| | | | | |
|---|---|---|---|---|
| 11.165X | 96.21% | 89.94% | 63.78% | 22.88% |
| 11.0095X | 96.17% | 89.66% | 62.44% | 21.65% |
| 12.3525X | 96.33% | 91.37% | 71.37% | 32.39% |
| 12.3985X | 96.29% | 91.40% | 71.78% | 32.99% |
| 9.6197X | 95.94% | 86.90% | 50.33% | 12.01% |
| 12.3447X | 96.20% | 91.05% | 71.23% | 33.16% |
| 10.8047X | 95.96% | 89.02% | 61.19% | 20.70% |
| 16.2125X | 96.52% | 93.69% | 84.89% | 63.04% |
| 18.2051X | 96.65% | 94.38% | 87.96% | 72.92% |
| 18.7886X | 96.63% | 94.42% | 88.49% | 74.95% |
| 17.2992X | 96.65% | 94.18% | 86.85% | 68.71% |
| 14.5423X | 96.41% | 92.80% | 80.51% | 51.57% |
| 16.4757X | 96.59% | 93.81% | 85.33% | 64.25% |
| 15.881X | 96.58% | 93.67% | 84.19% | 60.50% |
| 13.9734X | 96.36% | 92.46% | 78.89% | 47.14% |
| 14.4265X | 96.51% | 93.05% | 80.74% | 50.46% |
| 17.5958X | 96.67% | 94.30% | 87.34% | 70.29% |
| 15.6686X | 96.51% | 93.43% | 83.69% | 59.68% |
| 15.2115X | 96.53% | 93.32% | 82.75% | 56.40% |
| 13.9179X | 96.41% | 92.43% | 78.53% | 46.58% |
| 16.8736X | 96.59% | 93.97% | 86.05% | 66.66% |
| 15.3783X | 96.60% | 93.55% | 83.25% | 57.20% |
| 14.0295X | 96.35% | 92.49% | 79.05% | 47.63% |
| 13.9238X | 96.43% | 92.52% | 78.68% | 46.54% |
| 16.3234X | 96.62% | 93.84% | 85.17% | 63.35% |
| 15.4385X | 96.61% | 93.55% | 83.36% | 57.62% |
| 16.8681X | 96.62% | 94.05% | 86.15% | 66.47% |
| 11.2017X | 96.22% | 89.93% | 63.89% | 23.09% |
| 11.243X | 96.19% | 89.99% | 64.25% | 23.62% |
| 25.2287X | 96.84% | 95.28% | 92.59% | 86.92% |
| 27.1083X | 96.90% | 95.48% | 93.34% | 88.59% |
| 8.2373X | 95.75% | 82.08% | 35.76% | 5.65% |
| 8.626X | 96.03% | 84.34% | 39.27% | 6.50% |
| 7.2021X | 95.68% | 76.90% | 23.64% | 2.36% |
| 7.5792X | 95.76% | 79.35% | 27.78% | 3.15% |
| 8.2648X | 95.92% | 82.97% | 35.07% | 4.98% |
| 9.5895X | 96.26% | 87.42% | 49.13% | 11.04% |
| 8.8794X | 96.19% | 85.33% | 41.51% | 7.37% |
| 9.5632X | 96.34% | 87.50% | 48.46% | 10.61% |
| 9.6611X | 96.27% | 87.56% | 49.85% | 11.53% |
| 8.3983X | 95.96% | 83.46% | 36.79% | 5.61% |
| 8.2377X | 96.08% | 82.93% | 34.56% | 4.89% |
| 5.9029X | 95.21% | 65.12% | 11.32% | 0.86% |
| 7.8906X | 95.83% | 80.73% | 31.13% | 4.14% |
| 11.3581X | 96.43% | 90.76% | 64.52% | 23.15% |
| 9.1706X | 96.17% | 86.29% | 45.10% | 8.82% |
| 11.2197X | 96.49% | 90.63% | 63.75% | 22.12% |
| 10.139X | 96.30% | 88.68% | 54.57% | 14.52% |
| 9.3628X | 96.23% | 86.75% | 46.82% | 9.86% |
| 8.8494X | 96.07% | 85.19% | 41.54% | 7.34% |
| 8.221X | 95.87% | 82.53% | 34.77% | 5.05% |
| 11.087X | 96.14% | 89.65% | 63.46% | 22.75% |

| | | | | |
|---|---|---|---|---|
| 10.2832X | 96.10% | 88.32% | 56.81% | 16.82% |
| 12.8402X | 96.25% | 91.50% | 73.91% | 37.81% |
| 13.131X | 96.36% | 92.06% | 75.64% | 39.60% |
| 14.4389X | 96.44% | 92.62% | 79.58% | 50.29% |
| 11.9422X | 96.35% | 90.91% | 68.95% | 29.37% |
| 10.8065X | 96.27% | 89.30% | 60.52% | 19.99% |
| 11.3692X | 96.24% | 90.09% | 65.04% | 24.61% |
| 11.195X | 96.32% | 90.09% | 63.91% | 22.95% |
| 12.7105X | 96.35% | 91.52% | 73.17% | 36.12% |
| 14.3302X | 96.57% | 92.94% | 80.04% | 49.10% |
| 13.3798X | 96.48% | 92.31% | 76.27% | 41.06% |
| 12.8288X | 96.44% | 91.78% | 73.71% | 36.68% |
| 11.3503X | 96.22% | 90.17% | 65.26% | 24.66% |
| 10.999X | 96.22% | 89.77% | 62.59% | 21.46% |
| 9.9665X | 96.13% | 87.79% | 53.18% | 13.85% |
| 12.821X | 96.36% | 91.69% | 73.84% | 37.06% |
| 11.2488X | 96.18% | 89.88% | 63.87% | 23.88% |
| 11.4547X | 96.15% | 90.05% | 66.02% | 26.22% |
| 12.6026X | 96.34% | 91.63% | 72.98% | 35.08% |
| 8.0853X | 95.69% | 80.85% | 33.79% | 5.36% |
| 6.2192X | 94.87% | 68.03% | 15.25% | 1.23% |
| 8.3428X | 95.65% | 82.27% | 36.83% | 6.23% |
| 5.938X | 94.58% | 64.78% | 13.10% | 1.04% |
| 7.0538X | 95.34% | 75.26% | 22.90% | 2.37% |
| 7.7444X | 95.57% | 79.76% | 30.25% | 3.95% |
| 6.9421X | 95.33% | 74.58% | 21.59% | 2.13% |
| 8.587X | 95.93% | 83.76% | 38.94% | 6.69% |
| 8.188X | 95.73% | 81.68% | 34.92% | 5.57% |
| 8.5314X | 95.79% | 83.28% | 38.59% | 6.76% |
| 8.9234X | 95.88% | 84.75% | 42.66% | 8.41% |
| 7.1631X | 95.37% | 75.68% | 23.95% | 2.70% |
| 9.2695X | 96.08% | 85.78% | 45.53% | 10.05% |
| 7.9471X | 95.76% | 80.68% | 31.83% | 4.52% |
| 10.6853X | 96.22% | 89.34% | 59.41% | 19.01% |
| 7.8028X | 95.63% | 79.15% | 30.49% | 4.55% |
| 12.6902X | 96.40% | 91.69% | 73.21% | 35.72% |
| 13.5575X | 96.51% | 92.47% | 77.10% | 42.48% |
| 12.201X | 96.41% | 91.21% | 70.19% | 31.04% |
| 12.0735X | 96.34% | 91.06% | 69.59% | 30.28% |
| 14.0743X | 96.48% | 92.57% | 78.20% | 46.37% |
| 16.5476X | 96.75% | 93.98% | 85.39% | 63.51% |
| 13.3267X | 96.56% | 92.36% | 76.11% | 40.38% |
| 15.6798X | 96.67% | 93.70% | 83.80% | 58.34% |
| 13.2337X | 96.55% | 92.41% | 76.11% | 39.90% |
| 12.0588X | 96.22% | 91.01% | 69.82% | 30.54% |
| 14.0261X | 96.60% | 92.93% | 79.29% | 46.61% |
| 12.6992X | 96.43% | 91.91% | 73.71% | 35.67% |
| 13.3909X | 96.32% | 92.02% | 76.66% | 42.34% |
| 14.7096X | 96.43% | 92.86% | 81.21% | 52.77% |
| 14.2558X | 96.53% | 92.83% | 79.99% | 48.76% |
| 15.2347X | 96.66% | 93.50% | 82.89% | 55.89% |
| 14.9097X | 96.65% | 93.48% | 82.00% | 52.86% |

| | | | | |
|---|---|---|---|---|
| 10.351X | 96.23% | 88.76% | 56.77% | 16.62% |
| 13.8261X | 96.52% | 92.68% | 78.47% | 45.28% |
| 13.0014X | 96.35% | 91.85% | 74.87% | 39.06% |
| 13.7917X | 96.47% | 92.54% | 78.44% | 45.24% |
| 14.2053X | 96.52% | 92.82% | 79.84% | 48.45% |
| 14.9X | 96.58% | 93.26% | 82.00% | 53.65% |
| 14.4582X | 96.51% | 92.96% | 80.69% | 50.36% |
| 13.1793X | 96.41% | 92.02% | 75.25% | 39.89% |
| 12.2671X | 96.37% | 91.37% | 70.92% | 32.04% |
| 12.2826X | 96.40% | 91.40% | 70.88% | 32.06% |
| 11.0583X | 96.12% | 89.71% | 63.14% | 22.50% |
| 18.0489X | 96.63% | 94.23% | 87.60% | 72.24% |
| 20.3103X | 96.73% | 94.77% | 89.96% | 79.77% |
| 17.3872X | 96.53% | 93.99% | 86.74% | 69.48% |
| 19.1221X | 96.58% | 94.32% | 88.54% | 75.94% |
| 18.1289X | 96.50% | 94.11% | 87.58% | 72.53% |
| 18.6718X | 96.66% | 94.41% | 88.34% | 74.55% |
| 18.7278X | 96.64% | 94.44% | 88.56% | 75.08% |
| 18.9783X | 96.67% | 94.52% | 88.82% | 76.06% |
| 9.4794X | 96.26% | 86.91% | 47.79% | 10.85% |
| 9.0358X | 96.23% | 85.67% | 42.90% | 8.25% |
| 9.576X | 96.25% | 87.38% | 48.98% | 11.10% |
| 6.4502X | 95.42% | 70.38% | 16.05% | 1.38% |
| 7.7989X | 95.55% | 79.28% | 30.82% | 4.52% |
| 11.2674X | 95.97% | 89.44% | 63.94% | 25.12% |
| 9.0124X | 95.84% | 84.81% | 43.41% | 9.07% |
| 9.8086X | 96.01% | 87.29% | 51.89% | 13.48% |
| 10.1817X | 96.10% | 88.35% | 55.45% | 15.64% |
| 8.9487X | 95.91% | 84.86% | 43.08% | 8.61% |
| 8.8632X | 95.89% | 84.64% | 42.22% | 8.04% |
| 6.9835X | 95.35% | 74.84% | 22.13% | 2.22% |
| 8.9573X | 96.36% | 85.15% | 40.49% | 7.54% |
| 9.957X | 96.37% | 88.32% | 52.37% | 13.09% |
| 9.5252X | 96.37% | 87.34% | 48.14% | 10.62% |
| 9.273X | 96.33% | 86.72% | 45.71% | 9.21% |
| 19.9168X | 96.85% | 94.90% | 89.86% | 78.25% |
| 18.3733X | 96.68% | 94.41% | 88.16% | 73.39% |
| 19.0255X | 96.71% | 94.56% | 88.84% | 75.75% |
| 18.0617X | 96.69% | 94.41% | 87.87% | 72.18% |
| 9.1359X | 96.29% | 86.14% | 43.95% | 8.63% |
| 9.1066X | 96.25% | 85.94% | 43.85% | 8.61% |
| 9.4323X | 96.25% | 87.11% | 47.54% | 10.14% |
| 8.3808X | 95.92% | 82.88% | 37.22% | 6.18% |
| 12.2459X | 96.43% | 91.45% | 70.88% | 31.60% |
| 10.9652X | 96.45% | 90.17% | 61.62% | 20.23% |
| 12.7567X | 96.39% | 91.86% | 73.91% | 36.16% |
| 10.8764X | 96.15% | 89.58% | 61.76% | 20.76% |
| 12.6713X | 96.37% | 91.71% | 73.37% | 35.65% |
| 12.464X | 96.32% | 91.34% | 71.62% | 33.88% |
| 12.4403X | 96.40% | 91.58% | 72.30% | 33.66% |
| 12.119X | 96.34% | 91.20% | 70.41% | 30.78% |
| 13.7514X | 96.45% | 92.37% | 77.67% | 44.56% |

| | | | | |
|---|---|---|---|---|
| 12.3307X | 96.29% | 91.17% | 71.01% | 32.69% |
| 12.8742X | 96.40% | 91.79% | 74.15% | 37.27% |
| 11.2142X | 96.09% | 89.54% | 63.81% | 24.05% |
| 12.4825X | 96.38% | 91.50% | 72.07% | 33.78% |
| 13.0618X | 96.53% | 92.16% | 74.77% | 37.96% |
| 12.8528X | 96.43% | 91.82% | 74.01% | 36.85% |
| 14.1509X | 96.49% | 92.77% | 79.43% | 47.82% |
| 12.3179X | 96.31% | 91.07% | 70.91% | 32.68% |
| 12.5553X | 96.28% | 91.45% | 72.66% | 34.81% |
| 12.2605X | 96.34% | 91.23% | 71.06% | 32.30% |
| 10.7567X | 96.15% | 89.10% | 60.47% | 20.02% |
| 7.1491X | 95.61% | 75.16% | 22.79% | 2.64% |
| 6.9773X | 95.25% | 74.57% | 22.31% | 2.34% |
| 7.45X | 95.40% | 77.96% | 27.23% | 3.23% |
| 6.4556X | 94.85% | 70.00% | 17.70% | 1.63% |
| 8.4351X | 96.06% | 83.28% | 36.65% | 5.94% |
| 7.6794X | 95.98% | 79.89% | 28.58% | 3.48% |
| 7.0584X | 95.70% | 75.87% | 22.16% | 2.15% |
| 7.2771X | 95.82% | 77.42% | 24.28% | 2.51% |
| 12.271X | 96.33% | 91.11% | 70.31% | 32.30% |
| 14.3582X | 96.52% | 92.87% | 80.11% | 49.58% |
| 10.0954X | 96.10% | 88.22% | 54.93% | 14.84% |
| 13.823X | 96.46% | 92.63% | 78.43% | 45.06% |
| 13.499X | 96.48% | 92.34% | 77.06% | 42.45% |
| 11.3396X | 96.20% | 89.94% | 64.96% | 24.62% |
| 10.6308X | 96.10% | 88.93% | 59.71% | 18.98% |
| 14.3296X | 96.42% | 92.79% | 80.11% | 49.58% |
| 8.2925X | 96.00% | 82.76% | 35.20% | 5.31% |
| 8.7674X | 96.03% | 84.80% | 40.76% | 7.19% |
| 8.3603X | 96.03% | 83.17% | 36.01% | 5.51% |
| 8.2975X | 95.94% | 82.81% | 35.41% | 5.33% |
| 17.3972X | 96.73% | 94.19% | 86.84% | 68.79% |
| 9.4466X | 96.06% | 86.44% | 48.22% | 11.02% |
| 11.8384X | 96.18% | 90.57% | 68.40% | 28.89% |
| 11.1527X | 96.11% | 89.61% | 63.80% | 23.48% |
| 9.2663X | 96.09% | 86.51% | 46.44% | 9.52% |
| 10.1435X | 96.26% | 88.68% | 54.76% | 14.51% |
| 9.8101X | 96.28% | 88.07% | 51.48% | 12.18% |
| 9.7852X | 96.26% | 87.95% | 51.21% | 12.10% |
| 11.8739X | 96.35% | 90.68% | 68.13% | 28.85% |
| 13.2983X | 96.43% | 92.11% | 76.00% | 40.90% |
| 10.6035X | 96.09% | 88.84% | 59.30% | 19.10% |
| 11.1951X | 96.20% | 89.84% | 64.07% | 23.45% |
| 29.2726X | 96.92% | 95.61% | 93.87% | 89.92% |
| 28.1507X | 97.00% | 95.67% | 93.74% | 89.35% |
| 11.9647X | 96.24% | 90.80% | 69.12% | 29.88% |
| 14.8666X | 96.55% | 93.18% | 81.89% | 53.72% |
| 7.4036X | 95.52% | 77.70% | 26.39% | 3.04% |
| 8.4744X | 95.69% | 83.05% | 38.43% | 6.50% |
| 16.1192X | 96.56% | 93.65% | 84.79% | 62.17% |
| 11.0622X | 96.31% | 90.02% | 62.76% | 21.52% |
| 10.0067X | 95.93% | 87.49% | 54.01% | 15.01% |

| | | | | |
|---|---|---|---|---|
| 11.0381X | 96.17% | 89.73% | 62.93% | 22.03% |
| 10.8558X | 96.20% | 89.45% | 61.09% | 20.31% |
| 11.7542X | 96.21% | 90.59% | 67.79% | 28.01% |
| 10.2622X | 95.97% | 88.04% | 56.32% | 16.63% |
| 12.0273X | 96.30% | 90.93% | 69.28% | 30.21% |
| 11.1859X | 96.13% | 89.88% | 63.96% | 23.12% |
| 9.0844X | 95.77% | 85.19% | 45.11% | 9.25% |
| 14.9283X | 96.56% | 93.36% | 82.08% | 53.60% |
| 8.3801X | 95.75% | 82.88% | 37.42% | 5.94% |
| 10.105X | 96.08% | 88.02% | 54.70% | 15.29% |
| 9.8795X | 96.02% | 87.59% | 52.74% | 13.61% |
| 12.2715X | 96.28% | 91.07% | 70.94% | 32.62% |
| 10.6562X | 96.11% | 89.14% | 59.69% | 18.94% |
| 12.8262X | 96.26% | 91.52% | 73.93% | 37.76% |
| 13.4147X | 96.37% | 92.20% | 76.79% | 42.46% |
| 12.8578X | 96.34% | 91.79% | 74.24% | 37.25% |
| 13.5742X | 96.39% | 92.25% | 77.35% | 43.88% |
| 16.0424X | 96.60% | 93.70% | 84.59% | 61.42% |
| 10.5017X | 96% of | 88.48% | 58.72% | 18.67% |
| 8.3553X | 95.79% | 82.92% | 37.03% | 5.81% |
| 9.415X | 95.94% | 86.32% | 48.34% | 11.02% |
| 14.669X | 96.47% | 92.94% | 81.08% | 51.91% |
| 11.3046X | 96.24% | 90.23% | 65.01% | 24.02% |
| 10.4468X | 96.13% | 88.30% | 57.31% | 18.19% |
| 14.328X | 96.48% | 92.78% | 80.08% | 49.58% |
| 12.1366X | 96.26% | 91.09% | 70.74% | 31.29% |
| 13.0365X | 96.28% | 91.78% | 75.10% | 39.14% |
| 13.1225X | 96.36% | 91.98% | 75.72% | 39.97% |
| 12.7468X | 96.21% | 91.28% | 73.63% | 37.24% |
| 15.3837X | 96.47% | 93.32% | 83.04% | 57.05% |
| 11.7054X | 96.20% | 90.41% | 67.89% | 27.94% |
| 8.0591X | 95.89% | 81.47% | 33.28% | 4.81% |
| 14.9002X | 96.52% | 93.08% | 81.68% | 53.68% |
| 10.6177X | 96.44% | 89.67% | 59.10% | 17.82% |
| 7.8231X | 95.92% | 80.86% | 30.42% | 3.74% |
| 14.7156X | 96.56% | 93.08% | 81.30% | 52.23% |
| 9.3658X | 96.28% | 86.96% | 47.11% | 9.91% |
| 7.9008X | 96.04% | 81.07% | 30.69% | 4.01% |
| 8.6225X | 96.07% | 84.17% | 39.05% | 6.67% |
| 14.8473X | 96.59% | 93.20% | 81.76% | 53.31% |
| 9.9751X | 96.29% | 88.30% | 53.08% | 13.51% |
| 9.0265X | 96.12% | 85.85% | 43.74% | 8.24% |
| 14.1064X | 96.52% | 92.78% | 78.96% | 47.18% |
| 10.3427X | 96.01% | 88.20% | 57.39% | 17.43% |
| 12.3019X | 96.35% | 91.15% | 71.20% | 32.63% |
| 12.3283X | 96.40% | 91.48% | 71.51% | 32.64% |
| 12.4219X | 96.40% | 91.39% | 71.91% | 33.52% |
| 13.1044X | 96.49% | 92.10% | 75.38% | 39.15% |
| 13.4895X | 96.35% | 92.26% | 76.88% | 42.64% |
| 12.5318X | 96.55% | 91.85% | 71.47% | 33.16% |
| 11.5011X | 96.25% | 90.47% | 66.24% | 25.68% |
| 11.5131X | 96.52% | 90.88% | 65.25% | 24.66% |

| | | | | |
|---|---|---|---|---|
| 11.0088X | 96.40% | 90.11% | 61.71% | 20.87% |
| 18.7167X | 96.76% | 94.70% | 88.77% | 74.64% |
| 10.3619X | 96.40% | 89.16% | 56.37% | 15.91% |
| 6.5318X | 95.44% | 70.20% | 17.50% | 1.75% |
| 7.1541X | 95.74% | 75.61% | 22.93% | 2.58% |
| 20.1024X | 96.80% | 94.84% | 89.86% | 78.99% |
| 5.8914X | 95.30% | 64.37% | 11.42% | 0.96% |
| 6.8809X | 95.77% | 73.69% | 20.11% | 2.11% |
| 9.5416X | 96.21% | 86.99% | 47.99% | 10.99% |
| 8.4522X | 96.02% | 83.21% | 36.75% | 6.03% |
| 21.279X | 96.73% | 94.92% | 90.69% | 81.84% |
| 9.4152X | 96.26% | 86.70% | 46.73% | 10.25% |
| 9.4037X | 96.23% | 86.83% | 47.16% | 10.20% |
| 9.061X | 96.13% | 85.63% | 43.40% | 8.49% |
| 17.7453X | 96.69% | 94.35% | 87.57% | 71.05% |
| 9.4185X | 96.10% | 86.66% | 47.28% | 10.41% |
| 9.5412X | 96.13% | 87.13% | 48.63% | 11.09% |
| 9.1854X | 96.23% | 86.12% | 44.64% | 9.06% |
| 18.9155X | 96.67% | 94.53% | 88.71% | 75.24% |
| 11.8436X | 96.49% | 91.10% | 67.03% | 27.17% |
| 8.6138X | 96.01% | 83.71% | 38.50% | 6.81% |
| 10.5158X | 96.32% | 89.16% | 57.50% | 17.42% |
| 18.2983X | 96.66% | 94.44% | 88.17% | 73.22% |
| 9.8341X | 96.22% | 87.69% | 51.12% | 12.86% |
| 14.3792X | 96.56% | 92.94% | 80.04% | 49.22% |
| 14.0258X | 96.54% | 92.71% | 78.76% | 46.41% |
| 19.3172X | 96.71% | 94.71% | 89.27% | 76.72% |
| 14.2714X | 96.54% | 92.91% | 79.67% | 48.23% |
| 13.5801X | 96.49% | 92.44% | 77.16% | 42.76% |
| 16.0238X | 96.60% | 93.90% | 84.84% | 61.28% |
| 13.9915X | 96.51% | 92.68% | 78.81% | 46.30% |
| 13.3475X | 96.35% | 92.10% | 76.25% | 41.48% |
| 18.284X | 96.65% | 94.40% | 88.10% | 73.18% |
| 13.0972X | 96.34% | 91.79% | 74.91% | 39.56% |
| 12.5593X | 96.34% | 91.37% | 72.45% | 34.81% |
| 17.7834X | 96.68% | 94.34% | 87.41% | 70.38% |
| 13.2234X | 96.42% | 92.16% | 75.79% | 39.99% |
| 14.6X | 96.53% | 93.07% | 80.95% | 51.36% |
| 19.2771X | 96.77% | 94.71% | 89.18% | 76.60% |
| 13.652X | 96.39% | 92.36% | 77.43% | 43.75% |
| 12.727X | 96.45% | 91.69% | 73.15% | 35.72% |
| 15.2739X | 96.44% | 93.11% | 82.65% | 56.65% |
| 18.0566X | 96.74% | 94.50% | 88.00% | 72.25% |
| 16.3907X | 96.62% | 93.76% | 85.30% | 64.00% |
| 19.6085X | 96.73% | 94.66% | 89.41% | 77.66% |
| 14.4836X | 96.42% | 92.73% | 80.49% | 51.01% |
| 14.2835X | 96.44% | 92.69% | 79.85% | 49.20% |
| 11.7247X | 96.15% | 90.36% | 67.81% | 28.15% |
| 17.4483X | 96.61% | 94.11% | 86.88% | 69.38% |
| 13.4709X | 96.41% | 92.18% | 76.91% | 42.55% |
| 11.2749X | 96.17% | 90.13% | 64.75% | 23.53% |
| 17.5038X | 96.71% | 94.30% | 87.13% | 69.53% |

| | | | | |
|---|---|---|---|---|
| 12.8051X | 96.44% | 91.89% | 73.87% | 36.35% |
| 15.6456X | 96.54% | 93.44% | 83.70% | 58.88% |
| 15.3724X | 96.44% | 93.17% | 82.83% | 57.58% |
| 18.9151X | 96.65% | 94.47% | 88.63% | 75.38% |
| 18.4021X | 96.74% | 94.51% | 88.21% | 73.24% |
| 19.9572X | 96.70% | 94.65% | 89.58% | 78.62% |
| 19.0088X | 96.73% | 94.59% | 88.87% | 75.83% |
| 12.3793X | 96.43% | 91.64% | 71.31% | 32.30% |
| 12.8586X | 96.35% | 92.09% | 74.13% | 36.39% |
| 13.0698X | 96.47% | 92.32% | 75.18% | 38.33% |
| 11.1614X | 96.23% | 90.12% | 63.54% | 22.47% |
| 10.7464X | 96.22% | 89.55% | 60.80% | 19.33% |
| 11.1069X | 96.33% | 90.10% | 63.30% | 21.93% |
| 13.3552X | 96.42% | 92.33% | 76.75% | 41.70% |
| 12.0005X | 96.33% | 90.92% | 68.90% | 29.78% |
| 14.2079X | 96.52% | 93.00% | 79.75% | 47.89% |
| 13.4823X | 96.46% | 92.55% | 77.11% | 42.12% |
| 12.8248X | 96.49% | 92.13% | 73.78% | 35.86% |
| 11.624X | 96.41% | 91.00% | 66.85% | 25.73% |
| 18.3035X | 96.67% | 94.36% | 87.91% | 72.72% |
| 23.8633X | 96.85% | 95.26% | 92.11% | 85.59% |
| 18.6372X | 96.68% | 94.51% | 88.52% | 74.67% |
| 20.1088X | 96.79% | 94.88% | 89.98% | 79.23% |
| 20.0383X | 96.80% | 94.90% | 89.92% | 78.72% |
| 18.3891X | 96.69% | 94.42% | 88.16% | 73.48% |
| 20.4075X | 96.75% | 94.81% | 90.07% | 79.93% |
| 17.7121X | 96.64% | 94.29% | 87.43% | 70.98% |
| 18.7333X | 96.68% | 94.50% | 88.55% | 74.92% |
| 18.5963X | 96.71% | 94.48% | 88.40% | 74.27% |
| 20.4433X | 96.76% | 94.80% | 90.00% | 79.79% |
| 18.6477X | 96.64% | 94.39% | 88.36% | 74.74% |
| 11.0478X | 96.22% | 89.93% | 62.72% | 21.85% |
| 6.1475X | 94.93% | 67.67% | 14.20% | 1.08% |
| 7.7166X | 95.55% | 79.69% | 30.06% | 3.82% |
| 7.4201X | 95.37% | 77.79% | 27.09% | 3.11% |
| 8.8593X | 95.73% | 84.72% | 42.55% | 8.03% |
| 8.2907X | 95.73% | 82.45% | 36.13% | 5.63% |
| 9.2831X | 95.83% | 85.85% | 47.20% | 10.57% |
| 8.295X | 95.84% | 82.63% | 36.03% | 5.53% |
| 22.5515X | 96.86% | 95.18% | 91.44% | 83.64% |
| 20.6748X | 96.75% | 94.86% | 90.19% | 80.14% |
| 19.8602X | 96.64% | 94.55% | 89.41% | 77.89% |
| 18.4835X | 96.68% | 94.43% | 88.18% | 73.65% |
| 19.3652X | 96.70% | 94.66% | 89.26% | 77.04% |
| 20.8025X | 96.68% | 94.77% | 90.25% | 80.55% |
| 21.6025X | 96.79% | 95.04% | 90.88% | 82.14% |
| 19.2704X | 96.66% | 94.54% | 88.99% | 76.55% |
| 8.2216X | 95.93% | 82.54% | 35.06% | 5.30% |
| 7.5661X | 95.83% | 79.05% | 27.58% | 3.30% |
| 7.0236X | 95.74% | 75.73% | 21.76% | 2.10% |
| 8.5618X | 96.07% | 84.15% | 38.47% | 6.19% |
| 5.4291X | 94.77% | 59.82% | 8.28% | 0.57% |

| | | | | |
|---|---|---|---|---|
| 8.2363X | 96.03% | 82.66% | 35.10% | 5.18% |
| 9.2333X | 96.28% | 86.65% | 45.50% | 9.01% |
| 9.7093X | 96.16% | 87.48% | 51.02% | 12.36% |
| 4.0839X | 93.05% | 38.57% | 2.47% | 0.31% |
| 4.1039X | 93.10% | 39.50% | 2.36% | 0.26% |
| 5.3653X | 94.84% | 58.77% | 7.83% | 0.56% |
| 4.9184X | 94.31% | 52.66% | 5.37% | 0.39% |
| 4.803X | 94.29% | 50.88% | 4.67% | 0.40% |
| 5.2992X | 94.73% | 58.23% | 7.40% | 0.49% |
| 6.1029X | 95.40% | 67.67% | 12.97% | 0.95% |
| 5.6455X | 95.07% | 62.32% | 9.53% | 0.70% |
| 6.5473X | 95.60% | 71.59% | 16.96% | 1.46% |
| 6.1359X | 95.28% | 67.87% | 13.52% | 1.01% |
| 6.3437X | 95.47% | 70.09% | 15.13% | 1.19% |
| 5.3784X | 94.92% | 58.94% | 7.73% | 0.56% |
| 3.6865X | 92.15% | 32.44% | 1.37% | 0.20% |
| 3.7305X | 92.17% | 32.92% | 1.57% | 0.24% |
| 3.6941X | 92.24% | 32.58% | 1.39% | 0.21% |
| 4.0151X | 93.02% | 37.83% | 2.12% | 0.28% |
| 5.7161X | 95.09% | 63.13% | 10.16% | 0.72% |
| 5.626X | 95.02% | 62.19% | 9.59% | 0.67% |
| 5.5695X | 95.03% | 61.34% | 9.12% | 0.67% |
| 5.2783X | 94.64% | 57.92% | 7.43% | 0.49% |
| 5.7365X | 95.04% | 63.79% | 10.25% | 0.69% |
| 6.1148X | 95.41% | 67.50% | 12.96% | 1.00% |
| 6.6352X | 95.56% | 72.64% | 17.82% | 1.52% |
| 6.1623X | 95.43% | 68.20% | 13.51% | 1.05% |
| 5.1076X | 94.50% | 55.40% | 6.25% | 0.45% |
| 4.9457X | 94.39% | 53.14% | 5.34% | 0.40% |
| 5.7693X | 95.13% | 63.90% | 10.58% | 0.75% |
| 4.3335X | 93.51% | 43.73% | 2.92% | 0.27% |
| 3.9034X | 92.63% | 36.49% | 1.74% | 0.21% |
| 5.6881X | 94.96% | 63.17% | 9.94% | 0.66% |
| 6.212X | 95.38% | 68.86% | 14.08% | 1.03% |
| 5.7634X | 95.09% | 63.19% | 10.82% | 0.82% |
| 6.7973X | 95.59% | 74.06% | 19.62% | 1.71% |
| 6.0616X | 95.40% | 67.31% | 12.77% | 0.94% |
| 7.3054X | 95.79% | 77.74% | 24.92% | 2.61% |
| 5.9287X | 95.25% | 65.92% | 11.58% | 0.81% |
| 4.393X | 93.57% | 44.42% | 3.28% | 0.29% |
| 5.7567X | 95.17% | 63.91% | 10.47% | 0.70% |
| 4.7459X | 94.31% | 50.19% | 4.40% | 0.36% |
| 5.8506X | 95.31% | 64.78% | 10.91% | 0.79% |
| 5.6776X | 95.24% | 62.90% | 9.59% | 0.69% |
| 5.5721X | 94.99% | 61.71% | 9.04% | 0.62% |
| 6.9463X | 95.77% | 75.26% | 20.85% | 1.90% |
| 5.9002X | 95.20% | 65.75% | 11.40% | 0.76% |
| 5.2449X | 94.57% | 57.61% | 7.17% | 0.48% |
| 5.9866X | 95.14% | 66.34% | 12.44% | 0.90% |
| 7.1064X | 95.72% | 76.38% | 22.62% | 2.20% |
| 6.8308X | 95.67% | 74.33% | 19.66% | 1.74% |
| 5.6795X | 95.08% | 62.97% | 9.72% | 0.69% |

| | | | | |
|---|---|---|---|---|
| 4.1823X | 93.27% | 41.00% | 2.43% | 0.26% |
| 5.7085X | 95.14% | 63.15% | 10.01% | 0.72% |
| 4.8236X | 94.22% | 51.33% | 4.84% | 0.37% |
| 4.8368X | 94.33% | 51.58% | 4.81% | 0.39% |
| 5.8916X | 95.27% | 65.19% | 11.22% | 0.82% |
| 6.5287X | 95.58% | 71.87% | 16.78% | 1.34% |
| 5.9133X | 95.36% | 65.41% | 11.22% | 0.84% |
| 7.8768X | 95.96% | 81.05% | 30.65% | 3.90% |
| 7.0928X | 95.89% | 76.10% | 21.93% | 2.17% |
| 6.8714X | 95.63% | 74.53% | 20.19% | 1.85% |
| 6.5651X | 95.56% | 71.99% | 17.01% | 1.41% |
| 5.5047X | 95.08% | 60.62% | 8.39% | 0.64% |
| 5.8715X | 95.36% | 64.97% | 10.95% | 0.83% |
| 6.0283X | 95.32% | 66.78% | 12.30% | 0.90% |
| 6.2977X | 95.52% | 69.40% | 14.63% | 1.17% |
| 5.7095X | 95.18% | 62.78% | 9.89% | 0.76% |
| 6.025X | 95.25% | 66.83% | 12.50% | 0.91% |
| 5.2759X | 94.77% | 57.53% | 7.24% | 0.54% |
| 4.7154X | 94.18% | 49.73% | 4.26% | 0.36% |
| 5.9027X | 95.13% | 65.49% | 11.37% | 0.80% |
| 6.6171X | 95.20% | 72.36% | 18.26% | 1.52% |
| 6.5401X | 95.60% | 71.83% | 16.71% | 1.36% |
| 7.0614X | 95.73% | 75.93% | 22.23% | 2.19% |
| 5.4578X | 94.90% | 60.19% | 8.30% | 0.57% |
| 5.5534X | 94.99% | 61.37% | 8.86% | 0.63% |
| 6.3493X | 95.47% | 70.20% | 15.11% | 1.12% |
| 3.3495X | 90.93% | 26.68% | 0.88% | 0.17% |
| 3.0974X | 90.00% | 22.36% | 0.61% | 0.15% |
| 4.884X | 94.42% | 52.22% | 5.06% | 0.39% |
| 4.6067X | 94.01% | 48.07% | 3.84% | 0.34% |
| 4.9268X | 94.41% | 52.81% | 5.29% | 0.41% |
| 6.6866X | 95.59% | 73.07% | 18.50% | 1.57% |
| 6.5839X | 95.54% | 72.28% | 17.30% | 1.42% |
| 6.5899X | 95.64% | 72.21% | 17.06% | 1.42% |
| 5.1379X | 94.69% | 55.93% | 6.23% | 0.46% |
| 4.568X | 93.97% | 47.50% | 3.66% | 0.31% |
| 4.974X | 94.55% | 53.49% | 5.52% | 0.41% |
| 5.0705X | 94.60% | 55.03% | 5.98% | 0.44% |
| 5.5461X | 95.13% | 61.11% | 8.68% | 0.65% |
| 5.783X | 94.92% | 62.85% | 11.67% | 0.91% |
| 5.7163X | 95.21% | 62.96% | 9.99% | 0.75% |
| 5.7634X | 95.19% | 63.52% | 10.40% | 0.79% |
| 5.3323X | 94.83% | 58.47% | 7.51% | 0.52% |
| 5.5219X | 95.02% | 60.98% | 8.67% | 0.60% |
| 6.579X | 95.61% | 71.96% | 17.27% | 1.47% |
| 8.2173X | 96.14% | 82.76% | 34.21% | 4.89% |
| 7.4914X | 96.04% | 78.92% | 26.34% | 2.93% |
| 6.7744X | 95.69% | 73.54% | 19.25% | 1.76% |
| 6.2926X | 95.47% | 69.35% | 14.66% | 1.17% |
| 6.7153X | 95.74% | 73.14% | 18.31% | 1.64% |
| 4.7574X | 94.32% | 50.18% | 4.44% | 0.37% |
| 5.8582X | 95.20% | 64.92% | 11.08% | 0.78% |

| | | | | |
|---|---|---|---|---|
| 5.66X | 95.19% | 62.01% | 9.74% | 0.75% |
| 6.8529X | 95.73% | 74.13% | 19.82% | 1.86% |
| 6.868X | 95.70% | 74.29% | 20.29% | 1.91% |
| 11.9674 X | 96.33% | 91.24% | 69.39% | 29.10% |
| 12.355X | 96.31% | 91.46% | 71.52% | 32.60% |
| 13.5424 X | 96.55% | 92.66% | 77.37% | 42.50% |
| 12.9999 X | 96.38% | 92.14% | 75.00% | 38.11% |
| 12.0178 X | 96.23% | 91.06% | 69.89% | 30.11% |
| 13.8904 X | 96.42% | 92.72% | 78.86% | 45.92% |
| 13.6575 X | 96.50% | 92.66% | 77.91% | 43.60% |
| 14.3005 X | 96.48% | 92.86% | 80.03% | 49.16% |
| 12.8749 X | 96.40% | 91.91% | 74.25% | 37.47% |
| 11.5942 X | 96.34% | 90.73% | 66.78% | 25.97% |
| 13.0206 X | 96.45% | 92.17% | 75.22% | 38.44% |
| 12.6392 X | 96.37% | 91.74% | 73.29% | 35.46% |
| 11.7698 X | 96.27% | 90.98% | 68.20% | 27.69% |
| 12.2188 X | 96.26% | 91.31% | 70.94% | 31.81% |
| 12.2444 X | 96.35% | 91.48% | 71.07% | 31.60% |
| 12.963X | 96.46% | 92.16% | 74.77% | 37.71% |
| 12.164X | 96.43% | 91.48% | 70.29% | 30.72% |
| 11.1256 X | 96.29% | 90.12% | 63.53% | 22.47% |
| 10.7999 X | 96.19% | 89.66% | 61.21% | 20.05% |
| 9.9608X | 96.11% | 88.05% | 53.52% | 13.81% |
| 10.1033 X | 95.97% | 87.91% | 55.61% | 15.66% |
| 13.1289 X | 96.40% | 91.98% | 75.60% | 39.94% |
| 12.918X | 96.19% | 91.53% | 74.41% | 38.56% |
| 12.032X | 96.26% | 90.84% | 69.78% | 30.71% |
| 11.2273 X | 96.27% | 90.16% | 64.34% | 23.50% |
| 12.0593 X | 96.34% | 91.18% | 69.92% | 30.19% |
| 12.5486 X | 96.43% | 91.72% | 72.58% | 34.32% |
| 11.6275 X | 96.25% | 90.61% | 67.26% | 26.75% |
| 12.4634 X | 96.34% | 91.30% | 72.00% | 34.33% |
| 13.0766 X | 96.27% | 91.65% | 75.14% | 40.16% |
| 11.4154 X | 96.15% | 90.08% | 65.96% | 25.66% |
| 13.1844 X | 96.36% | 91.92% | 75.70% | 40.72% |
| 13.4826 X | 96.34% | 92.13% | 77.01% | 43.15% |
| 13.4259 X | 96.35% | 92.30% | 76.91% | 42.29% |
| 12.3093 X | 96.26% | 91.41% | 71.57% | 32.57% |

| | | | | |
|---|---|---|---|---|
| 10.3624 X | 96.20% | 88.78% | 57.20% | 16.72% |
| 11.6578 X | 96.10% | 89.86% | 66.44% | 28.69% |
| 13.2009 X | 96.38% | 92.10% | 75.98% | 40.81% |
| 12.3844 X | 96.32% | 91.30% | 71.78% | 33.57% |
| 20.18X | 96.67% | 94.68% | 89.67% | 78.40% |
| 11.0959 X | 96.28% | 90.10% | 63.33% | 22.09% |
| 10.336X | 96.11% | 88.68% | 57.17% | 16.66% |
| 11.1776 X | 96.20% | 90.17% | 63.87% | 22.72% |
| 9.9705X | 96.16% | 88.11% | 53.27% | 13.62% |
| 11.7864 X | 96.20% | 90.63% | 68.40% | 28.67% |
| 11.8354 X | 96.23% | 90.51% | 68.16% | 29.20% |
| 11.4405 X | 96.14% | 89.99% | 65.78% | 25.91% |
| 12.7607 X | 96.36% | 91.80% | 73.79% | 36.60% |
| 10.2973 X | 96.06% | 88.00% | 56.36% | 17.47% |

| | | | | |
|---|---|---|---|---|
| 9.8523X | 95.84% | 86.93% | 52.93% | 14.50% |
| 9.8803X | 95.97% | 87.21% | 52.96% | 14.27% |
| 8.9915X | 95.63% | 84.37% | 44.58% | 9.63% |
| 9.7576X | 95.97% | 87.07% | 51.95% | 13.43% |
| 12.0817 X | 96.26% | 90.85% | 69.84% | 31.17% |
| 11.9534 X | 96.28% | 90.82% | 69.25% | 29.94% |
| 10.7149 X | 96.09% | 88.93% | 60.37% | 20.17% |
| 10.4872 X | 96.12% | 88.65% | 58.50% | 18.36% |
| 10.7483 X | 95.94% | 88.67% | 60.57% | 21.06% |
| 11.7425 X | 96.10% | 90.26% | 67.71% | 28.57% |
| 10.9471 X | 96.15% | 89.38% | 62.00% | 21.82% |
| 9.5998X | 95.86% | 86.61% | 50.67% | 12.47% |
| 10.2642 X | 96.08% | 88.15% | 56.24% | 16.79% |
| 10.6483 X | 96.18% | 89.17% | 60.02% | 19.14% |
| 11.2054 X | 96.28% | 90.09% | 64.18% | 23.27% |
| 10.0735 X | 95.94% | 87.67% | 54.95% | 15.62% |
| 10.166X | 96.08% | 88.05% | 55.72% | 15.98% |
| 10.3933 X | 96.10% | 88.51% | 57.63% | 17.61% |
| 7.8667X | 95.48% | 80.01% | 32.39% | 4.62% |
| 11.5907 X | 96.20% | 90.33% | 67.00% | 27.08% |
| 12.339X | 96.20% | 90.88% | 71.12% | 34.21% |
| 12.2892 X | 96.35% | 91.27% | 71.23% | 32.69% |
| 12.14X | 96.27% | 91.02% | 70.33% | 31.56% |
| 11.5218 X | 96.13% | 90.06% | 66.41% | 26.75% |
| 12.8402 X | 96.26% | 91.57% | 74.01% | 37.87% |
| 12.7602 X | 96.29% | 91.55% | 73.47% | 36.80% |
| 10.9102 X | 96.04% | 89.00% | 61.93% | 22.23% |
| 12.5209 X | 96.19% | 91.06% | 72.37% | 35.54% |
| 14.8748 X | 96.40% | 92.78% | 81.09% | 54.11% |
| 16.5021 X | 96.57% | 93.73% | 85.19% | 64.51% |
| 14.4546 X | 96.40% | 92.43% | 79.29% | 50.55% |
| 13.6745 X | 96.24% | 91.98% | 77.25% | 45.17% |
| 14.6767 X | 96.41% | 92.80% | 80.78% | 52.66% |
| 12.8725 X | 96.26% | 91.49% | 73.95% | 37.97% |
| 12.8866 X | 96.31% | 91.64% | 74.27% | 38.19% |
| 11.2734 X | 96.19% | 89.82% | 64.20% | 24.25% |
| 14.5437 X | 96.49% | 92.95% | 80.83% | 51.51% |
| 9.7862X | 96.07% | 87.60% | 52.18% | 12.83% |

| | | | | |
|---|---|---|---|---|
| 12.4009 X | 96.32% | 91.52% | 72.15% | 33.32% |
| 12.3258 X | 96.31% | 91.27% | 71.51% | 33.01% |
| 12.2784 X | 96.17% | 90.80% | 70.85% | 33.40% |
| 14.6272 X | 96.41% | 92.79% | 80.77% | 52.27% |
| 13.3286 X | 96.28% | 91.89% | 76.26% | 42.25% |
| 11.7007 X | 96.12% | 90.29% | 67.87% | 28.47% |
| 13.7339 X | 96.29% | 92.06% | 77.53% | 45.41% |
| 12.4183 X | 96.24% | 91.13% | 71.87% | 34.29% |
| 12.2326 X | 96.26% | 90.99% | 70.97% | 32.83% |
| 13.7427 X | 96.25% | 92.02% | 77.58% | 45.65% |
| 10.7119 X | 96.03% | 88.78% | 60.17% | 20.40% |
| 13.9999 X | 96.31% | 92.10% | 78.22% | 47.73% |
| 11.7497 X | 96.09% | 90.18% | 67.87% | 29.02% |
| 11.7X | 96.14% | 90.36% | 67.79% | 28.28% |

| | | | | |
|---|---|---|---|---|
| 14.1716 X | 96.35% | 92.37% | 79.05% | 48.93% |
| 13.1187 X | 96.25% | 91.56% | 74.96% | 40.58% |
| 14.2738 X | 96.39% | 92.51% | 79.38% | 49.55% |
| 12.4884 X | 96.19% | 90.98% | 71.65% | 35.26% |
| 14.0524 X | 96.36% | 92.31% | 78.77% | 47.94% |
| 13.231X | 96.30% | 91.91% | 75.65% | 41.01% |
| 12.6351 X | 96.19% | 91.09% | 72.72% | 36.42% |
| 12.4119 X | 96.24% | 91.23% | 71.93% | 34.06% |
| 16.8639 X | 96.65% | 93.93% | 85.95% | 66.56% |
| 13.7268 X | 96.37% | 92.12% | 77.49% | 45.29% |
| 13.802X | 96.40% | 92.28% | 77.92% | 45.85% |
| 11.9251 X | 96.23% | 90.56% | 68.85% | 30.06% |
| 14.0936 X | 96.34% | 92.47% | 79.29% | 48.42% |
| 12.1134 X | 96.20% | 90.58% | 69.66% | 32.04% |
| 11.7803 X | 96.03% | 90.14% | 68.03% | 29.34% |
| 11.308X | 96.04% | 89.57% | 64.96% | 25.32% |
| 14.4692 X | 96.45% | 92.68% | 80.19% | 51.08% |
| 13.5844 X | 96.37% | 92.12% | 77.24% | 44.16% |
| 14.3212 X | 96.50% | 92.80% | 80.02% | 49.75% |
| 14.7218 X | 96.44% | 92.85% | 81.11% | 52.97% |
| 14.2823 X | 96.41% | 92.60% | 79.80% | 49.61% |
| 15.3891 X | 96.49% | 93.21% | 82.90% | 57.68% |
| 9.4807X | 95.84% | 86.26% | 49.38% | 11.78% |
| 9.4027X | 95.83% | 85.89% | 48.51% | 11.51% |
| 14.7056 X | 96.42% | 92.92% | 81.25% | 53.01% |
| 12.7558 X | 96.45% | 91.66% | 73.19% | 36.10% |
| 14.5755 X | 96.38% | 92.66% | 80.50% | 51.96% |
| 13.0568 X | 96.21% | 91.64% | 75.09% | 39.97% |
| 13.5458 X | 96.34% | 92.10% | 77.09% | 43.70% |
| 13.5559 X | 96.22% | 91.91% | 77.06% | 44.25% |
| 11.0471 X | 96.04% | 89.45% | 63.13% | 22.73% |
| 11.4983 X | 96.08% | 89.91% | 66.35% | 26.72% |
| 12.6433 X | 96.29% | 91.43% | 72.85% | 35.91% |
| 12.4268 X | 96.20% | 91.06% | 71.87% | 34.42% |
| 13.4232 X | 96.34% | 92.06% | 76.59% | 42.68% |
| 12.5642 X | 96.16% | 91.00% | 72.09% | 35.73% |
| 11.46X | 96.18% | 90.18% | 66.29% | 25.75% |

| | | | | |
|---|---|---|---|---|
| 11.6412 X | 96.07% | 90.06% | 67.22% | 27.99% |
| 10.2396 X | 95.93% | 87.97% | 56.73% | 16.77% |
| 11.2051 X | 96.15% | 89.89% | 64.52% | 23.74% |
| 13.9502 X | 96.29% | 92.16% | 78.35% | 47.48% |
| 12.438X | 96.24% | 91.22% | 72.16% | 34.36% |
| 12.4701 X | 96.16% | 90.93% | 71.89% | 35.37% |
| 11.1661 X | 96.06% | 89.40% | 63.74% | 24.12% |
| 12.1181 X | 96.25% | 90.87% | 70.23% | 31.66% |
| 11.7452 X | 96.19% | 90.42% | 68.00% | 28.43% |
| 10.35X | 96.03% | 88.35% | 57.64% | 17.47% |
| 10.7778 X | 96.04% | 89.04% | 61.27% | 20.64% |
| 11.5473 X | 96.15% | 89.97% | 65.94% | 27.25% |
| 12.8765 X | 96.28% | 91.61% | 74.45% | 38.30% |
| 12.9273 X | 96.23% | 91.52% | 74.39% | 38.92% |
| 11.7442 X | 96.08% | 90.18% | 67.70% | 28.94% |

| | | | | |
|---|---|---|---|---|
| 11.6992 X | 96.13% | 90.22% | 67.71% | 28.31% |
| 12.0306 X | 96.10% | 90.53% | 69.52% | 31.25% |
| 10.3863 X | 95.92% | 88.01% | 57.61% | 18.16% |
| 11.5372 X | 96.17% | 90.22% | 66.71% | 26.73% |
| 12.0649 X | 96.22% | 90.83% | 70.04% | 31.13% |
| 11.4187 X | 96.04% | 89.80% | 65.79% | 26.14% |
| 11.8595 X | 96.16% | 90.48% | 68.61% | 29.70% |
| 11.6594 X | 96.14% | 90.24% | 67.25% | 27.80% |
| 12.0561 X | 96.12% | 90.60% | 69.85% | 31.49% |
| 12.4684 X | 96.14% | 90.98% | 71.96% | 35.03% |
| 10.1872 X | 95.78% | 87.30% | 56.06% | 17.27% |
| 10.8624 X | 95.99% | 89.00% | 61.90% | 21.67% |
| 9.7484X | 95.72% | 86.56% | 52.15% | 14.04% |
| 12.051X | 96.25% | 90.96% | 70.09% | 31.00% |
| 11.7891 X | 96.17% | 90.22% | 67.31% | 29.10% |
| 11.2448 X | 96.13% | 89.72% | 64.37% | 24.37% |
| 13.3732 X | 96.25% | 91.82% | 76.21% | 42.55% |
| 12.9442 X | 96.18% | 91.42% | 74.24% | 38.86% |
| 12.8236 X | 96.20% | 91.29% | 73.66% | 38.01% |
| 12.4354 X | 96.23% | 90.88% | 70.90% | 34.52% |
| 13.5305 X | 96.26% | 91.92% | 76.67% | 43.86% |
| 10.5183 X | 96.19% | 88.98% | 58.67% | 17.92% |
| 13.4124 X | 96.37% | 92.14% | 76.77% | 42.48% |
| 10.9114 X | 96.15% | 89.54% | 62.20% | 21.32% |
| 12.8582 X | 96.24% | 91.49% | 74.13% | 38.12% |
| 13.0396 X | 96.27% | 91.60% | 74.56% | 39.57% |
| 13.6063 X | 96.40% | 92.29% | 77.50% | 44.20% |
| 11.3886 X | 96.02% | 89.78% | 65.68% | 25.82% |
| 12.4844 X | 96.12% | 90.91% | 71.92% | 35.25% |
| 12.8427 X | 96.29% | 91.57% | 74.05% | 37.74% |
| 13.3375 X | 96.37% | 92.09% | 76.47% | 41.80% |
| 14.3174 X | 96.40% | 92.68% | 80.05% | 49.86% |
| 11.1135 X | 96.14% | 89.69% | 63.60% | 23.10% |
| 14.3322 X | 96.27% | 92.32% | 79.47% | 50.18% |
| 14.1871 X | 96.25% | 92.27% | 79.02% | 48.99% |

| | | | | |
|---|---|---|---|---|
| 12.9793 X | 96.19% | 91.45% | 74.70% | 39.43% |
| 12.9756 X | 96.28% | 91.66% | 74.70% | 39.06% |
| 13.2601 X | 96.32% | 91.99% | 76.19% | 41.30% |
| 14.2239 X | 96.44% | 92.69% | 79.76% | 49.01% |
| 11.9764 X | 96.09% | 90.36% | 68.98% | 31.01% |
| 12.2338 X | 96.13% | 90.84% | 70.99% | 32.90% |
| 11.6135 X | 95.97% | 89.75% | 66.54% | 28.31% |
| 10.7399 X | 95.87% | 88.54% | 60.72% | 21.18% |
| 12.1904X | 96.18% | 90.96% | 70.86% | 32.24% |
| 15.0886X | 96.40% | 93.06% | 82.35% | 56.05% |
| 13.7205X | 96.29% | 92.12% | 77.83% | 45.39% |
| 13.1647X | 96.28% | 91.88% | 75.73% | 40.51% |
| 11.7684X | 96.00% | 89.87% | 67.64% | 29.65% |
| 13.8848X | 96.28% | 92.21% | 78.36% | 46.86% |
| 13.6322X | 96.24% | 91.79% | 76.83% | 44.89% |
| 12.1089X | 95.98% | 90.18% | 69.75% | 32.61% |
| 12.8678X | 96.19% | 91.25% | 73.75% | 38.54% |

| | | | | |
|---|---|---|---|---|
| 12.3459X | 96.16% | 90.82% | 71.14% | 34.01% |
| 12.5659X | 96.29% | 91.28% | 72.59% | 35.52% |
| 14.2437X | 96.47% | 92.77% | 79.92% | 49.29% |
| 13.9492X | 96.41% | 92.42% | 78.53% | 46.89% |
| 14.1901X | 96.43% | 92.62% | 79.49% | 48.69% |
| 14.4341X | 96.47% | 92.88% | 80.59% | 50.64% |
| 9.9029X | 95.95% | 87.26% | 53.27% | 14.50% |
| 10.2783X | 96.05% | 88.19% | 56.56% | 16.79% |
| 13.9277X | 96.38% | 92.44% | 78.75% | 46.96% |
| 11.5479X | 96.15% | 90.14% | 66.62% | 26.77% |
| 14.093X | 96.45% | 92.59% | 79.17% | 47.92% |
| 13.5888X | 96.49% | 92.30% | 77.53% | 44.05% |
| 11.827X | 96.09% | 90.38% | 68.50% | 29.27% |
| 11.8222X | 96.05% | 90.24% | 68.39% | 29.59% |
| 11.0812X | 96.13% | 89.50% | 63.26% | 23.01% |
| 16.4453X | 96.50% | 93.39% | 84.52% | 63.92% |
| 17.43X | 96.61% | 93.88% | 86.42% | 68.99% |
| 17.5414X | 96.67% | 94.02% | 86.72% | 69.48% |
| 17.6232X | 96.66% | 94.09% | 87.01% | 70.32% |
| 11.4639X | 96.28% | 90.35% | 65.44% | 25.34% |
| 10.6678X | 96.11% | 89.17% | 59.71% | 18.81% |
| 10.0617X | 95.99% | 87.82% | 54.48% | 14.90% |
| 9.8764X | 95.97% | 87.38% | 52.67% | 13.67% |
| 12.2299X | 96.36% | 91.34% | 71.12% | 31.98% |
| 13.0921X | 96.31% | 91.84% | 75.35% | 39.94% |
| 9.0542X | 95.85% | 85.24% | 44.45% | 8.95% |
| 15.9479X | 96.60% | 93.76% | 84.59% | 61.10% |
| 16.5574X | 96.61% | 93.84% | 85.42% | 64.48% |
| 14.432X | 96.45% | 92.89% | 80.47% | 50.46% |
| 24.8876X | 96.79% | 95.20% | 92.40% | 86.51% |
| 26.9718X | 96.87% | 95.43% | 93.25% | 88.46% |
| 28.408X | 96.97% | 95.62% | 93.68% | 89.40% |
| 27.6606X | 96.91% | 95.55% | 93.47% | 88.85% |
| 15.7091X | 96.63% | 93.60% | 83.68% | 58.77% |
| 13.3608X | 96.50% | 92.41% | 76.50% | 41.12% |
| 16.5281X | 96.64% | 93.91% | 85.41% | 64.10% |
| 11.5178X | 96.31% | 90.47% | 65.99% | 25.72% |
| 13.6506X | 96.50% | 92.45% | 77.57% | 43.65% |
| 15.7828X | 96.75% | 93.84% | 84.33% | 59.73% |
| 14.5626X | 96.61% | 93.07% | 80.64% | 50.53% |
| 16.7418X | 96.70% | 93.98% | 85.89% | 65.46% |
| 16.9112X | 96.70% | 94.10% | 86.15% | 66.09% |
| 13.7582X | 96.56% | 92.71% | 78.08% | 44.22% |
| 15.4812X | 96.86% | 93.86% | 83.43% | 56.88% |
| 12.3281X | 96.36% | 91.41% | 71.23% | 32.73% |
| 8.3586X | 95.96% | 82.98% | 36.20% | 5.68% |
| 9.9824X | 96.13% | 87.73% | 52.92% | 14.42% |
| 6.6649X | 95.44% | 73.04% | 18.23% | 1.49% |
| 7.2294X | 95.77% | 77.22% | 23.74% | 2.40% |
| 27.9851X | 96.90% | 95.39% | 93.26% | 88.80% |
| 23.9417X | 96.84% | 95.00% | 91.66% | 84.91% |
| 12.2371X | 96.33% | 91.29% | 71.14% | 32.03% |

| | | | | |
|---|---|---|---|---|
| 12.398X | 96.29% | 91.32% | 71.72% | 33.61% |
| 11.7955X | 96.25% | 90.71% | 68.03% | 27.86% |
| 11.4636X | 96.25% | 90.27% | 65.67% | 25.43% |
| 11.1838X | 96.13% | 89.81% | 63.73% | 23.08% |
| 10.6543X | 96.21% | 89.26% | 59.53% | 18.54% |
| 9.849X | 96.03% | 87.63% | 52.39% | 13.08% |
| 10.1187X | 96.07% | 87.96% | 54.86% | 15.48% |
| 10.864X | 96.17% | 89.56% | 61.29% | 20.20% |
| 11.5516X | 96.25% | 90.51% | 66.52% | 25.98% |
| 12.6371X | 96.32% | 91.45% | 72.89% | 35.96% |
| 12.9435X | 96.38% | 91.89% | 74.68% | 38.05% |
| 13.7917X | 96.47% | 92.58% | 78.41% | 45.40% |
| 11.3902X | 96.14% | 89.91% | 65.41% | 25.68% |
| 12.0941X | 96.24% | 90.94% | 70.34% | 31.63% |
| 12.3421X | 96.45% | 91.61% | 71.57% | 32.23% |
| 13.2826X | 96.38% | 92.12% | 76.29% | 41.21% |
| 13.5377X | 96.46% | 92.36% | 77.42% | 43.34% |
| 7.3329X | 95.38% | 77.32% | 26.14% | 2.89% |
| 7.2288X | 95.27% | 76.55% | 25.21% | 2.72% |
| 5.9585X | 94.61% | 65.89% | 12.94% | 0.90% |
| 8.65X | 95.83% | 84.12% | 40.28% | 6.83% |
| 9.1842X | 96.02% | 85.91% | 45.42% | 9.22% |
| 11.0873X | 96.24% | 89.91% | 63.21% | 22.13% |
| 10.0947X | 96.03% | 87.51% | 54.19% | 16.00% |
| 8.1469X | 95.82% | 82.02% | 34.50% | 5.03% |
| 9.939X | 96.10% | 87.88% | 53.47% | 13.91% |
| 17.8231X | 96.65% | 94.14% | 87.44% | 71.46% |
| 19.7124X | 96.79% | 94.71% | 89.54% | 77.92% |
| 17.0052X | 96.56% | 93.81% | 86.09% | 67.19% |
| 15.7052X | 96.54% | 93.41% | 83.80% | 59.66% |
| 13.0743X | 96.38% | 91.80% | 74.80% | 39.28% |
| 14.3089X | 96.44% | 92.75% | 80.01% | 49.39% |
| 14.7997X | 96.56% | 93.05% | 81.21% | 52.63% |
| 11.3825X | 96.16% | 90.02% | 65.41% | 25.11% |
| 12.8341X | 96.25% | 91.43% | 73.95% | 37.98% |
| 14.3953X | 96.56% | 92.92% | 80.29% | 49.65% |
| 13.9496X | 96.50% | 92.66% | 78.90% | 46.24% |
| 14.2993X | 96.63% | 93.09% | 80.17% | 48.57% |
| 14.4997X | 96.57% | 92.99% | 80.67% | 50.67% |
| 13.1861X | 96.48% | 92.14% | 75.81% | 39.94% |
| 13.5152X | 96.45% | 92.32% | 77.22% | 42.89% |
| 10.133X | 96.13% | 88.18% | 55.29% | 15.27% |
| 22.726X | 96.86% | 95.22% | 91.74% | 84.39% |
| 29.8166X | 96.99% | 95.72% | 94.09% | 90.33% |
| 23.9885X | 96.91% | 95.35% | 92.32% | 85.92% |
| 28.7061X | 97.01% | 95.69% | 93.87% | 89.73% |
| 14.2507X | 96.51% | 92.89% | 79.95% | 48.65% |
| 11.6559X | 96.36% | 90.67% | 67.30% | 26.71% |
| 12.1906X | 96.38% | 91.28% | 70.73% | 31.26% |
| 9.554X | 96.05% | 86.83% | 49.65% | 11.53% |
| 23.0847X | 96.83% | 95.19% | 91.82% | 84.83% |
| 29.5806X | 97.03% | 95.76% | 94.07% | 90.22% |

| | | | | |
|---|---|---|---|---|
| 26.7848X | 96.94% | 95.51% | 93.29% | 88.37% |
| 27.5059X | 96.93% | 95.52% | 93.42% | 88.78% |
| 14.72X | 96.43% | 92.94% | 81.12% | 52.36% |
